# Disease modification and symptom relief in osteoarthritis using a mutated GCP‐2/CXCL6 chemokine

**DOI:** 10.15252/emmm.202216218

**Published:** 2022-12-12

**Authors:** Sara Caxaria, Nikolaos Kouvatsos, Suzanne E Eldridge, Mario Alvarez‐Fallas, Anne‐Sophie Thorup, Daniela Cici, Aida Barawi, Ammaarah Arshed, Danielle Strachan, Giulia Carletti, Xinying Huang, Sabah Bharde, Melody Deniz, Jacob Wilson, Bethan L Thomas, Costantino Pitzalis, Francesco Paolo Cantatore, Manasi Sayilekshmy, Shafaq Sikandar, Frank P Luyten, Thomas Pap, Joanna C Sherwood, Anthony J Day, Francesco Dell'Accio

**Affiliations:** ^1^ William Harvey Research Institute, Barts and the London School of Medicine and Dentistry Queen Mary University of London London UK; ^2^ Wellcome Centre for Cell‐Matrix Research, Manchester Academic Health Science Centre University of Manchester Manchester UK; ^3^ Rheumatology Clinic, Department of Medical and Surgical Sciences University of Foggia Foggia Italy; ^4^ Department of Development and Regeneration, Skeletal Biology and Engineering Research Center KU Leuven Leuven Belgium; ^5^ Institute of Musculoskeletal Medicine University Hospital Münster Münster Germany; ^6^ Lydia Becker Institute of Immunology and Inflammation, Faculty of Biology, Medicine & Health, Manchester Academic Health Science Centre University of Manchester Manchester UK

**Keywords:** chemokine, chondrogenesis, CXCL6, GCP‐2, osteoarthritis, Musculoskeletal System

## Abstract

We showed that the chemokine receptor C‐X‐C Motif Chemokine Receptor 2 (CXCR2) is essential for cartilage homeostasis. Here, we reveal that the CXCR2 ligand granulocyte chemotactic protein 2 (GCP‐2) was expressed, during embryonic development, within the prospective permanent articular cartilage, but not in the epiphyseal cartilage destined to be replaced by bone. GCP‐2 expression was retained in adult articular cartilage. GCP‐2 loss‐of‐function inhibited extracellular matrix production. GCP‐2 treatment promoted chondrogenesis *in vitro* and in human cartilage organoids implanted in nude mice *in vivo*. To exploit the chondrogenic activity of GCP‐2, we disrupted its chemotactic activity, by mutagenizing a glycosaminoglycan binding sequence, which we hypothesized to be required for the formation of a GCP‐2 haptotactic gradient on endothelia. This mutated version (GCP‐2‐T) had reduced capacity to induce transendothelial migration *in vitro* and *in vivo*, without affecting downstream receptor signaling through AKT, and chondrogenic activity. Intra‐articular adenoviral overexpression of GCP‐2‐T, but not wild‐type GCP‐2, reduced pain and cartilage loss in instability‐induced osteoarthritis in mice. We suggest that GCP‐2‐T may be used for disease modification in osteoarthritis.

## Graphical abstract

Osteoarthritis, which is the most common cause of disability, leads to breakdown of the articular cartilage and causes joint pain and loss of mobility; however, we do not have a pharmacological treatment for arresting or reverting its course.

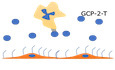

**The paper explained**

**Problem**

Osteoarthritis is a common and disabling joint disease characterized by joint pain and loss of mobility caused by cartilage breakdown often associated with bone changes and low‐grade inflammation. Osteoarthritis affects up to a third of the population over the age of 45 and costs around 1.5–2% of GDP in developed countries. Despite its high prevalence, there are no pharmacological interventions that can arrest or reverse progression of cartilage breakdown and avoid the need for joint replacement surgery. Therefore, new, and more effective drugs are needed that can target not only the disease pathology, but also pain which is the main debilitating symptom for patients.

**Results**

Here, we show that GCP‐2 regulates cartilage homeostasis. By mutating the GAG‐binding site of GCP‐2 we disrupted its pro‐inflammatory properties while preserving its capacity to support cartilage differentiation. Using this mutant in therapeutic regime, we reduced pain and improved cartilage integrity in a murine model of osteoarthritis.

**Impact**

We have developed a novel therapeutic molecule that improves symptoms and protects cartilage in experimental osteoarthritis. While other cartilage‐inducing growth factors, such as BMPs induce hypertrophic differentiation and ectopic bone formation, GCP‐2 promotes cartilage formation but prevents hypertrophic differentiation and mineralization.

## Introduction

Osteoarthritis is a chronically disabling joint disease characterized by cartilage breakdown, often associated with thickening of the subchondral bone, new bone formation (osteophytes), ligament damage, and low‐grade inflammation (Martel‐Pelletier *et al*, 2016). It causes joint pain, loss of mobility, affects up to a third of the population over the age of 45 and costs around 1.5–2% of GDP (Hiligsmann *et al*, 2013). Despite its high prevalence, there are no pharmacological interventions that can arrest or reverse progression of cartilage breakdown and avoid the need for joint replacement surgery. Cartilage extracellular matrix (ECM) is rich in glycosaminoglycans (GAGs) and collagens, which provide load‐bearing and tensile strength, respectively.

During development, long bones are initially made of cartilage. Subsequently, chondrocytes in the central part of the skeletal elements (diaphysis) become hypertrophic—expressing collagen type X and other markers—and are eventually replaced by bone. This process, called endochondral bone formation, spares the last few layers of cells proximal to the joint which are resistant to hypertrophic differentiation, vascular invasion, calcification and bone formation and give rise to the permanent articular cartilage (Thorup *et al*, 2020a). During osteoarthritis, however, ectopic and pathological chondrocyte hypertrophy and calcification take place within the articular cartilage and drive its breakdown (Saito *et al*, 2010; Yang *et al*, 2010; Bertrand *et al*, 2011, 2020).

We showed that the chemokine receptors CXCR1 and CXCR2, and their ligands are expressed in cartilage and contribute to cartilage homeostasis by an autocrine/paracrine mechanism that, through activation of AKT signaling, culminates in upregulation of the cartilage transcription factor SOX9 and of its target genes COL2A1 and ACAN (Sherwood *et al*, 2015). ELR^+^ CXC chemokines are characterized by a glutamic acid‐leucine‐arginine (ELR) motif. These chemokines bind and activate the seven‐transmembrane G protein‐coupled receptors CXCR1 and CXCR2, promoting inflammation through their ability to recruit and activate leukocytes (Bizzarri *et al*, 2006). During inflammation, ELR^+^ CXC chemokines bind to GAG chains present on the blood vessels at inflammatory sites (Handel & Dyer, 2021), thereby forming a haptotactic gradient on the endothelial surface. The engagement of the chemokines bound to the endothelial surface and the chemokine receptor on the membrane of leukocytes initiates the process of transendothelial migration through which leukocytes exit the blood vessel and accumulate in tissues at the site of inflammation (Luster, 1998; Handel & Dyer, 2021). Glycosaminoglycans binding, therefore, is essential for transendothelial migration of leukocytes.

Here, we show that disrupting the capacity of the CXCR2 ligand granulocyte chemotactic protein 2 (GCP‐2, also known as CXCL5 in mice and CXCL6 in humans) to bind GAGs abrogates its pro‐inflammatory effects, while preserving its capacity to support cartilage homeostasis. In an experimental osteoarthritis model in mice, GCP‐2‐T reduced pain and improved cartilage integrity.

## Results

### GCP‐2 is expressed in the prospective permanent articular cartilage in embryonic development and in adult cartilage

**Figure 1:**
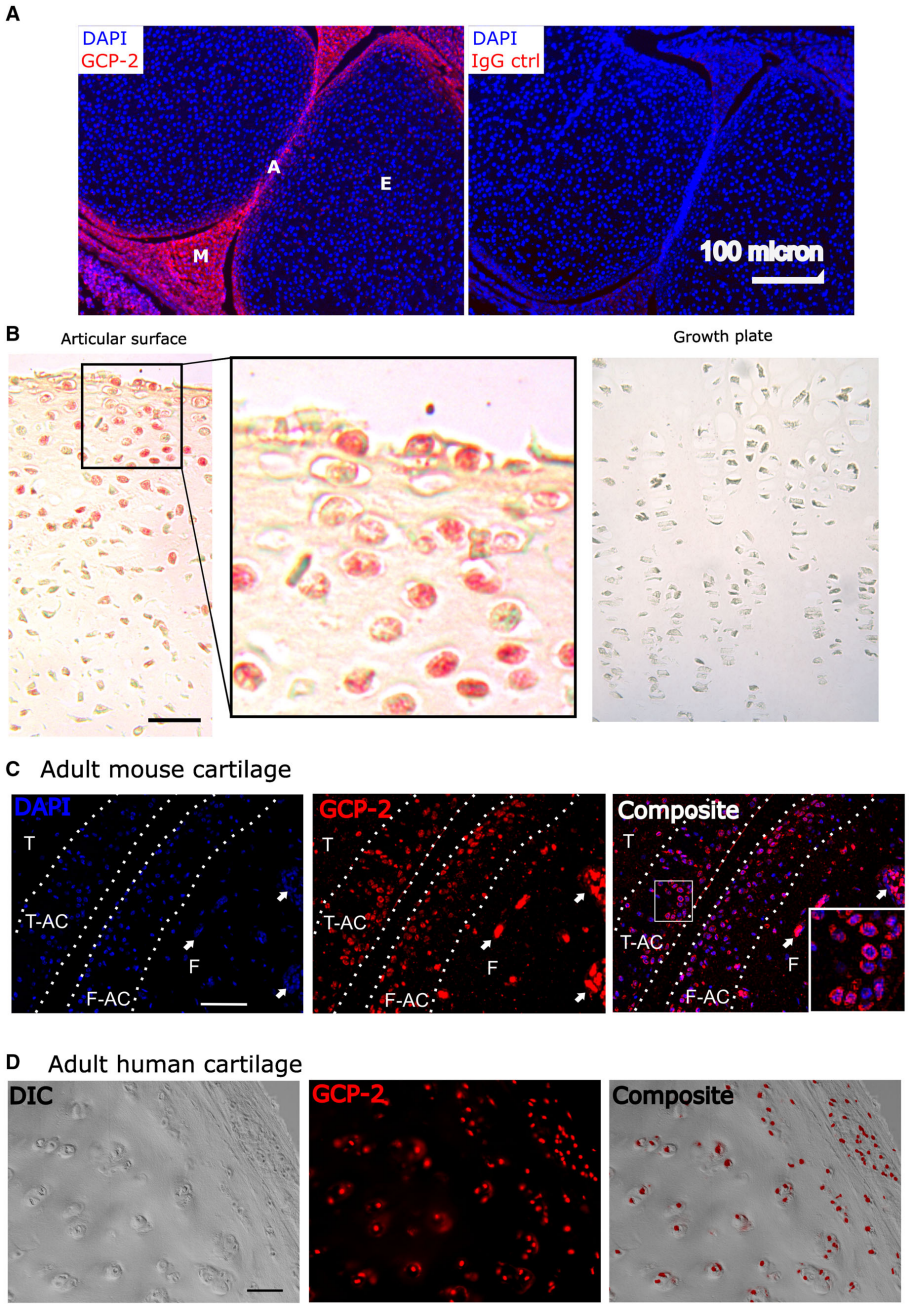
GCP‐2 is expressed in the prospective permanent articular cartilage in embryonic development and in adult cartilage AImmunostaining of a murine knee joint at 18.5 dpc with an anti‐GCP‐2 or IgG control antibody.BImmunohistochemistry for GCP‐2 (red) in the knee of a human 5‐month old fetus. Scale bar = 100 μm.C, DImmunostaining for GCP‐2 in an adult (12‐week‐old) mouse knee (C) or in adult human articular cartilage (D). Scale bar = 50 μm in (C) and 100 μm in (D). Data information: E—epiphyseal cartilage; A—articular cartilage; M—meniscus; T—tibia; F—femur; T‐AC—tibial articular cartilage; F‐AC—femur articular cartilage; in (C), dotted lines indicate the osteochondral boundary and the cartilage surface and the arrow the bone marrow spaces. Source data are available online for this figure.

To explore a possible function of GCP‐2 in cartilage biology, we first investigated its expression pattern during embryonic skeletal development. GCP‐2 was detected within the prospective articular cartilage, but not in the epiphyseal cartilage in mice and humans (Fig 1A and B). GCP‐2 staining was retained in adulthood in both species (Fig 1C and D).

This expression pattern during development is similar to that of the markers of prospective joint interzones, that is, GDF5 (Storm *et al*, 1994) and WNT9A (Hartmann & Tabin, 2001)—with expression well before the immune system is developed, suggesting that GCP‐2 may play a role in cartilage biology independent of its role in inflammation.

### GCP‐2 supports the articular chondrocyte phenotype and chondrogenic differentiation

**Figure 2:**
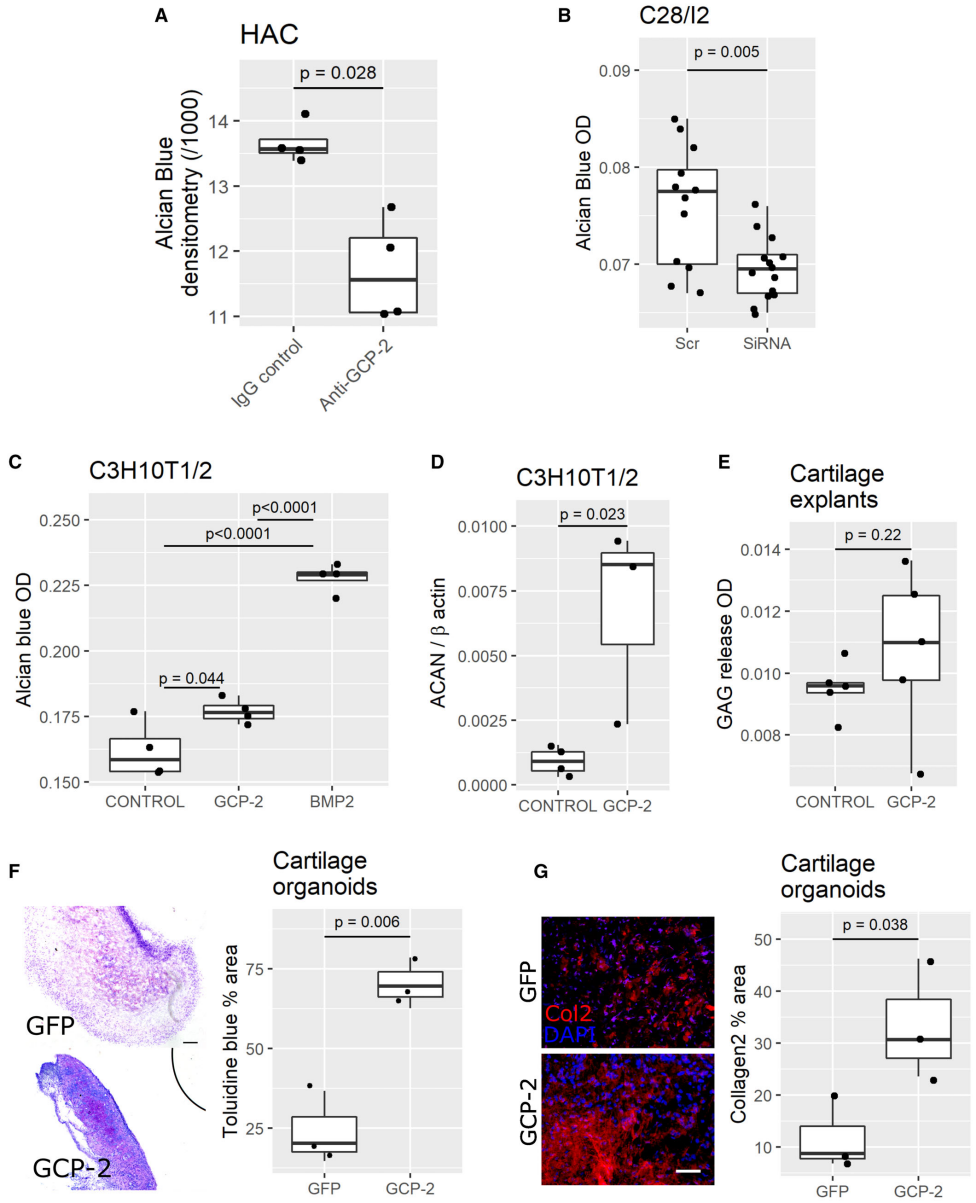
GCP‐2 supports the stable chondrocyte phenotype and chondrogenic differentiation *in vitro* and *in vivo* A–CGAG content as assessed by Alcian blue staining with densitometry (A) or spectrophotometric (B, C) quantitation. A HAC micromasses treated with a GCP‐2 neutralizing antibody (*n* = 4) or IgG control (*n* = 4); *P*‐values were determined by the Mann–Whitney *U*‐test. (B) C28/I2 micromasses treated with GCP‐2 siRNA (*n* = 12) or scrambled control siRNA (Scr; *n* = 14); *P*‐values were determined by unpaired two‐tailed Student's *t*‐test. (C) C3H10T½ micromasses treated for 3 days with recombinant GCP‐2100 ng/ml (*n* = 4) or vehicle (*n* = 4) or BMP‐2 (*n* = 4); *P*‐values were determined by ANOVA followed by Tukey HSD *post hoc* test.DqPCR for *ACAN* (Aggrecan) expression in C3H10T½ micromasses treated for 3 days with recombinant GCP‐2 (100 ng/ml) (*n* = 3) or vehicle (*n* = 4); *ACAN*—Aggrecan; *P*‐values were determined by unpaired two‐tailed Student's *t*‐test.ESpectrophotometric quantification of GAG release (dimethylmethylene blue assay) in supernatant of human cartilage explants treated with recombinant GCP‐2 (*n* = 5) or vehicle (*n* = 5) and incubated for 24 h; *P*‐values were determined by Mann–Whitney *U*‐test.FToluidine blue staining of ectopic cartilage explants collected 2 weeks after subcutaneous co‐implantation of HACs in a 10:1 ratio with growth‐arrested COS7 expressing either GFP (*n* = 3) or GCP‐2 (*n* = 3) and quantification—on the right‐hand side—of the metachromatically stained area as % of total area; scale bar = 200 μm; *P*‐values were determined by unpaired two‐tailed Student's *t*‐test.GCollagen 2 (Col2) immunostaining (left) and quantification (right) as % of total area; scale bar = 100 μm; *n* = 3 per group; *P*‐values were determined by unpaired two‐tailed Student's *t*‐test.
Source data are available online for this figure.

**Figure EV1:**
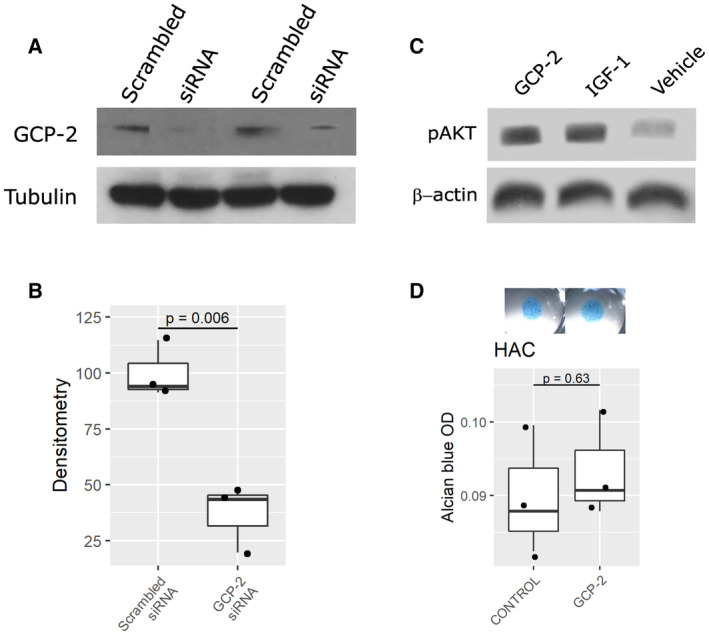
siRNA validation *in vitro*. Effect of GCP‐2 on AKT phosphorylation in chondrocytes and on GAG production in HACs A, BC28/I2 (A) or C3H10T1/2 (B) monolayers transfected with 25 nM of either scrambled or GCP‐2 siRNA for 72 h and analyzed for GCP‐2 levels by Western blotting. (A) A representative western blot; (B) densitometric quantification of three independent experiments. *N* = 3. *P*‐values were determined using the two‐tailed Student's *t*‐test.CC28/I2 micromasses stimulated for 3 days with recombinant GCP‐2, IGF‐1 (positive control) or untreated (vehicle) and assessed for AKT phosphorylation levels by Western blotting; β‐actin as loading control; GCP‐2—WT, GCP‐2‐treated samples, IGF‐1—IGF‐1‐treated samples, V—vehicle‐treated samples, pAKT—phosphorylated AKT.DAlcian blue staining and spectrophotometric quantitation of GAGs in micromasses of HACs treated with recombinant GCP‐2 or vehicle (*n* = 3); *P*‐values were determined by unpaired, two‐tailed Student's *t*‐test. OD—optical density. Source data are available online for this figure.

Articular chondrocytes express both GCP‐2 and its receptors CXCR1 and CXCR2 (Sherwood *et al*, 2015) suggesting autocrine/paracrine signaling. To investigate whether endogenous GCP‐2 is required to maintain the stable chondrocyte phenotype, we blocked endogenous GCP‐2 in primary human articular chondrocytes (HAC) using an anti‐GCP‐2 neutralizing antibody. After 24 h, GCP‐2 blockade resulted in GAG loss as assessed by Alcian blue staining (Fig 2A). Silencing GCP‐2 with siRNA (~75% efficiency—Fig EV1A and B) led to GAG loss in the human chondrogenic cell line C28/I2 (Fig 2B).

Next, we investigated whether exogenous GCP‐2 is sufficient to induce chondrogenic differentiation. Treatment of C3H10T½ micromasses with recombinant GCP‐2 (100 ng/ml for 3 days) resulted in accumulation of GAG‐rich ECM as measured by Alcian blue staining, albeit to a lesser extent than as induced by BMP‐2 (Fig 2C). It also resulted in an increase in AKT phosphorylation (Fig EV1C)—a known signaling event downstream of CXCR1/2 activation in chondrocytes (Sherwood *et al*, 2015).

We then investigated whether the effects of GCP‐2 treatment on ECM proteoglycan content were due to increased synthesis or reduced catabolism of ECM components. Treatment of C3H10T½ cells with GCP‐2 upregulated the expression of aggrecan mRNA (ACAN) (Fig 2D), a major proteoglycan in cartilage suggesting an anabolic function. Furthermore, treatment of human cartilage explants with GCP‐2 did not decrease GAG release in the supernatant as assessed by dimethylmethylene blue assay (Fig 2E), as would have been expected in the case of reduced catabolism. Taken together, these data indicate that the increase in ECM induced by GCP‐2 is due to an anabolic effect.

Although GCP‐2 blockade resulted in reduced ECM formation in HACs (Fig 2A), no significant increase in ECM content was detected when fully mature HACs were treated with exogenous GCP‐2 (Fig EV1D), possibly owing to their high endogenous GCP‐2 expression or ceiling effect.

To test whether GCP‐2 supports stable cartilage formation *in vivo*, we used an adoptive model in which HACs implanted ectopically in immunodeficient mice form stable human cartilage organoids resistant to vascular invasion, calcification, and endochondral bone formation (Dell'Accio *et al*, 2001; Eldridge *et al*, 2016; Thorup *et al*, 2020b, 2022).

To deliver exogenous GCP‐2 to the implanted HACs for the entire duration of the assay, we used a previously validated strategy based on co‐implantation of chondrocytes with growth‐arrested, GCP‐2‐overexpressing COS7 cells, or, as control, with GFP‐overexpressing COS7 cells (Eldridge *et al*, 2016). Two weeks after implantation, GCP‐2‐supplemented HAC implants displayed enhanced ECM formation as determined by the increased area of metachromatic Toluidine Blue staining (Fig 2F), and immunostaining for collagen type II (Fig 2G).

### GCP‐2 inhibits hypertrophic differentiation and calcification of articular chondrocytes

**Figure 3:**
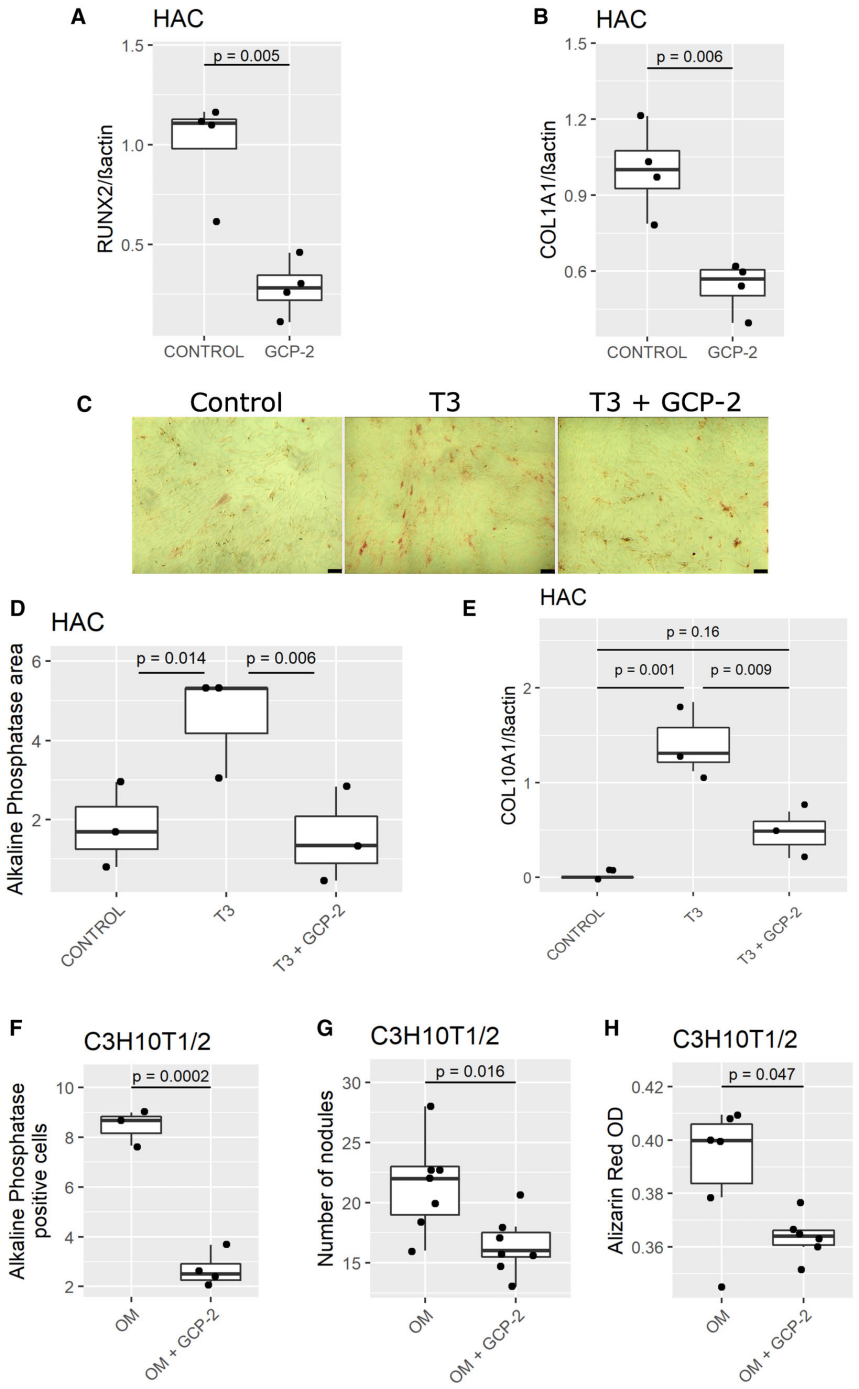
GCP‐2 prevents hypertrophic differentiation and calcification of chondrocytes A, BqPCR for (A) *Runx2* and (B) *Col1A1* expression in HAC micromasses treated for 24 h with recombinant GCP‐2 (100 ng/ml) or vehicle (*n* = 4); *Runx2*—Runt‐related transcription factor 2, *Col1A1—*collagen type 1 alpha 1; *n* = 4; *P*‐values were determined by unpaired two‐tailed Student's *t*‐test.CAlkaline phosphatase staining (red) in HAC monolayer after 3 week treatment with T3 hormone (100 ng/ml), T3 hormone + GCP‐2 (100 ng/ml), or control medium; scale bar 100 μm.DQuantification of alkaline phosphatase stained area (*n* = 3); *P*‐values were determined by fitting a generalized linear model followed by pairwise comparison of the estimated marginal means.EqPCR for *Col10A1* (*n* = 3) of HAC 3 weeks after treatment with vehicle, T3 (100 ng/ml) or T3 + GCP‐2 (100 ng/ml); *n* = 3; *P*‐values were determined by ANOVA with Tukey's HSD *post hoc* test.F–HC3H10T½ monolayers were treated with osteogenic medium (OM) or OM + GCP‐2 (100 ng/ml): (F) number of cells per well positive for alkaline phosphatase staining (*n* = 3 in OM and *n* = 4 in OM + GCP‐2), (G) number of alkaline phosphatase positive nodules per well (*n* = 7), and (H) spectrophotometric quantification of alizarin red staining at 570 nm (*n* = 6); *P*‐values were determined by unpaired, two‐tailed Student's *t*‐test. Source data are available online for this figure.

The expression pattern of GCP‐2 in development led us to hypothesize that GCP‐2 might prevent hypertrophic differentiation and calcification, a phenomenon driving osteoarthritis progression (Saito *et al*, 2010; Bertrand *et al*, 2011; Thorup *et al*, 2020a). Consistent with this hypothesis, exogenous GCP‐2 reduced the expression of the hypertrophy marker *Runx2* and of the osteogenic marker *Col1A1* mRNA in HAC micromasses (Fig 3A and B).

To further investigate this phenomenon, we tested whether GCP‐2 could counteract the capacity of thyroid hormone (T3) to promote hypertrophic chondrocyte differentiation (Robson *et al*, 2000). Consistent with this, we found that, in HAC, GCP‐2 blocked the capacity of T3 to induce alkaline phosphatase activity (Fig 3C and D), and COL10A1 (Fig 3E), two markers of chondrocyte hypertrophy (Thorup *et al*, 2020a).

GCP‐2 also reduced the capacity of osteogenic medium (Dell'Accio *et al*, 2003a) to induce osteogenesis in C3H10T½ cells as measured by a reduction in the number of alkaline phosphatase‐positive cells (Fig 3F) and calcified nodules as measured by Alizarin red staining (Fig 3G: number of nodules; Fig 3H: spectrophotometric quantification). Previous experiments showed that the calcification of chondrocytes or stem cells under standard conditions is negligible (De Bari *et al*, 2001b; Dell'Accio *et al*, 2003a).

### Disrupting GCP‐2 GAG binding dissociates the chemotactic from the chondrogenic effects of GCP‐2

Although GCP‐2 has a beneficial effect on cartilage homeostasis, its pro‐inflammatory properties would make it unsuitable as a therapeutic molecule for osteoarthritis. Therefore, we set out to dissociate the pro‐inflammatory from chondrogenic effects of GCP‐2.

Endothelial cells display GAGs on their surface, to which ELR^+^ CXC chemokines bind, creating a haptotactic gradient (Rot, 1992; Middleton *et al*, 1997). Neutrophils, displaying chemokine receptors, interact with and are activated by the chemokines immobilized on the endothelial surface and this interaction initiates transendothelial migration (Luster, 1998; Bizzarri *et al*, 2006; Proudfoot *et al*, 2017; Handel & Dyer, 2021).

**Figure 4:**
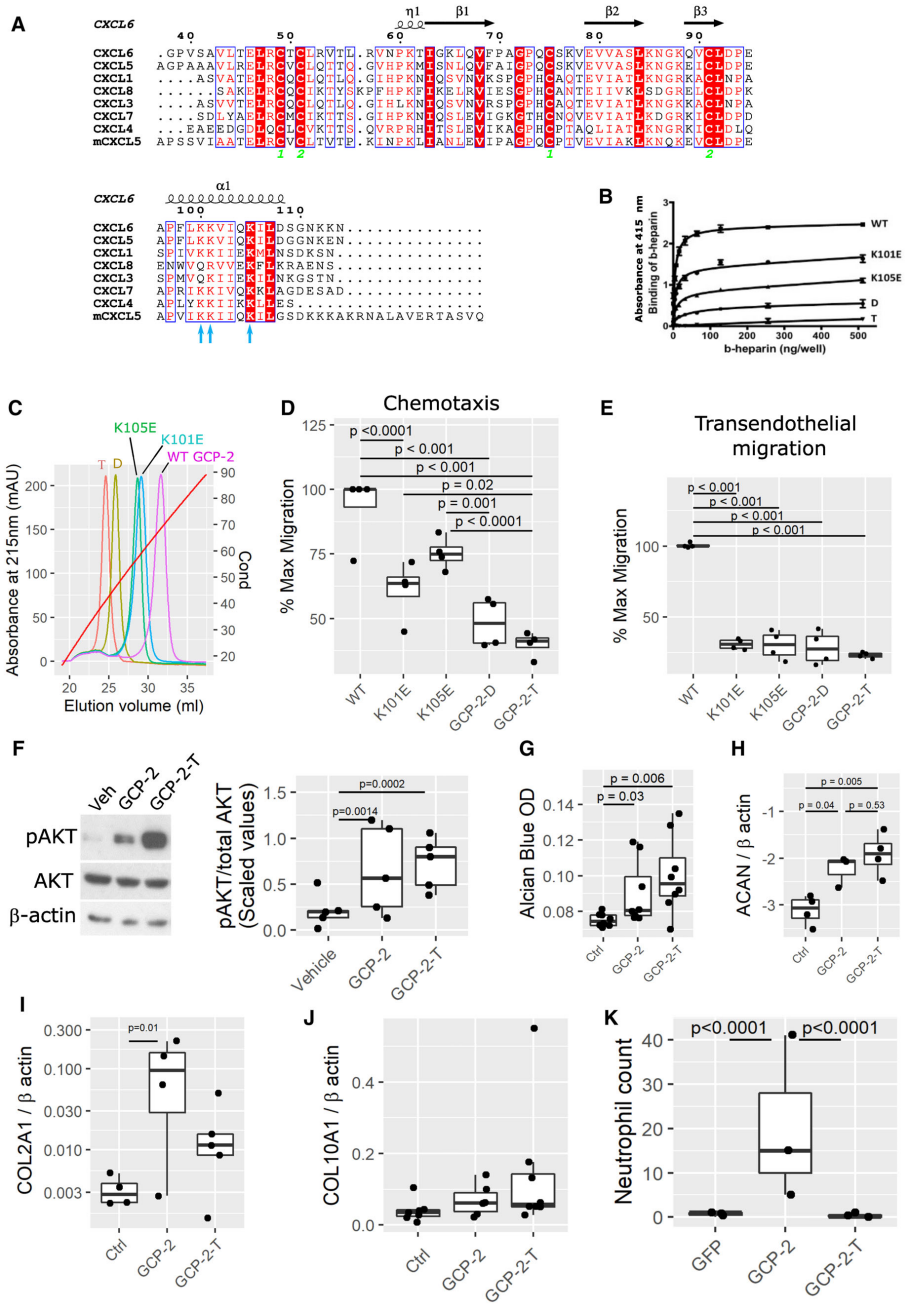
Disrupting GCP‐2 GAG binding dissociates the chemotactic from the chondrogenic effects of GCP‐2 AHuman GCP‐2 sequence aligned with closely related human CXCL proteins and the mouse CXCL5 (mCXCL5). The secondary structure is predicted based on the NMR structure of human CXCL5 (PDB: 2MGS). Conserved residues are highlighted in red boxes (white font) and partially conserved residues with similar chemical properties are shown in red font.B, CGAG binding capacity of wild‐type GCP‐2, and mutants (K101E single mutant, K105E single mutant, GCP‐2‐D = K101E_K105E double mutant and GCP‐2‐T = triple mutant K100E_ K101E_K105E) was assessed by (B) a heparin microtiter plate binding assay measuring the interaction of biotinylated‐heparin (b‐heparin) with immobilized proteins and (C) heparin affinity chromatography equilibrated and run in PBS, pH 7.4. In (C), the proteins are eluted with a salt gradient from 0 to 2 M NaCl monitored by the conductivity (Cond), where 20 and 90 mS/cm correspond to 193 and 1,360 mM NaCl, respectively; GCP‐2‐T, GCP‐2‐D, K101E, K105E and WT proteins elute at 530, 617, 815, 850 and 1,024 mM NaCl, respectively.D, EMaximum migratory activity of WT GCP‐2 (50 nM) was compared with GCP‐2 mutants (50 nM) in (D) chemotaxis and (E) transendothelial migration assays in CXCR2‐expressing 300‐19 pre‐B cells. WT—wild‐type GCP‐2; K101E—GCP‐2_K101E single mutant; K105E—GCP‐2_K105E single mutant; D—GCP‐2_K101E_K105E double mutant; T—GCP‐2_K100E_K101E_K105E triple mutant; *n* = 4; *P*‐values were determined by fitting a generalized linear model followed by pairwise comparison of the estimated marginal means.F–HC28/I2 micromasses stimulated with WT GCP‐2, GCP‐2 triple mutant (GCP‐2‐T) or untreated and assessed for (F) AKT phosphorylation levels by Western blotting; V—vehicle‐treated samples, GCP‐2—WT GCP‐2‐treated samples, GCP‐2‐T—triple mutant GCP‐2‐treated samples, pAKT—phosphorylated AKT, tAKT—total AKT. On the right, quantification of the density of the bands using ImageJ. Five independent experiments were analyzed. The data were scaled and *P*‐values were determined by fitting a generalized linear model followed by pairwise comparison of the estimated marginal means. (G) Spectrophotometric quantification of proteoglycan content by Alcian blue staining. *P*‐values were determined by Kruskal–Wallis test with Dunn's *post hoc* test; (*n* = 8). (H) *ACAN* expression levels by qPCR; *ACAN*—Aggrecan; *P*‐values were determined by ANOVA with Tukey's HSD *post hoc* test; *n* = 4.I, J Real time quantitative PCR of COL2A1 (I) and COL10A1 (J) mRNA normalized to β actin. *P*‐values were determined by fitting a generalized linear model followed by comparison of the estimated marginal means; *n* = 4 control, 5 GCP‐2 and GCP‐2‐T for COL2A1 and *n* = 6 per group for COL10A1.KNumber of neutrophils counted within the intercondylar space on sections from mice killed 4 days after an intra‐articular injection of adenovirus encoding GFP (*n* = 3), GCP‐2 (*n* = 3) and GCP‐2‐T (*n* = 3); *P*‐values were determined by fitting a generalized linear model (family = poisson) followed by pairwise comparison of the estimated marginal means. Source data are available online for this figure.

**Figure EV2:**
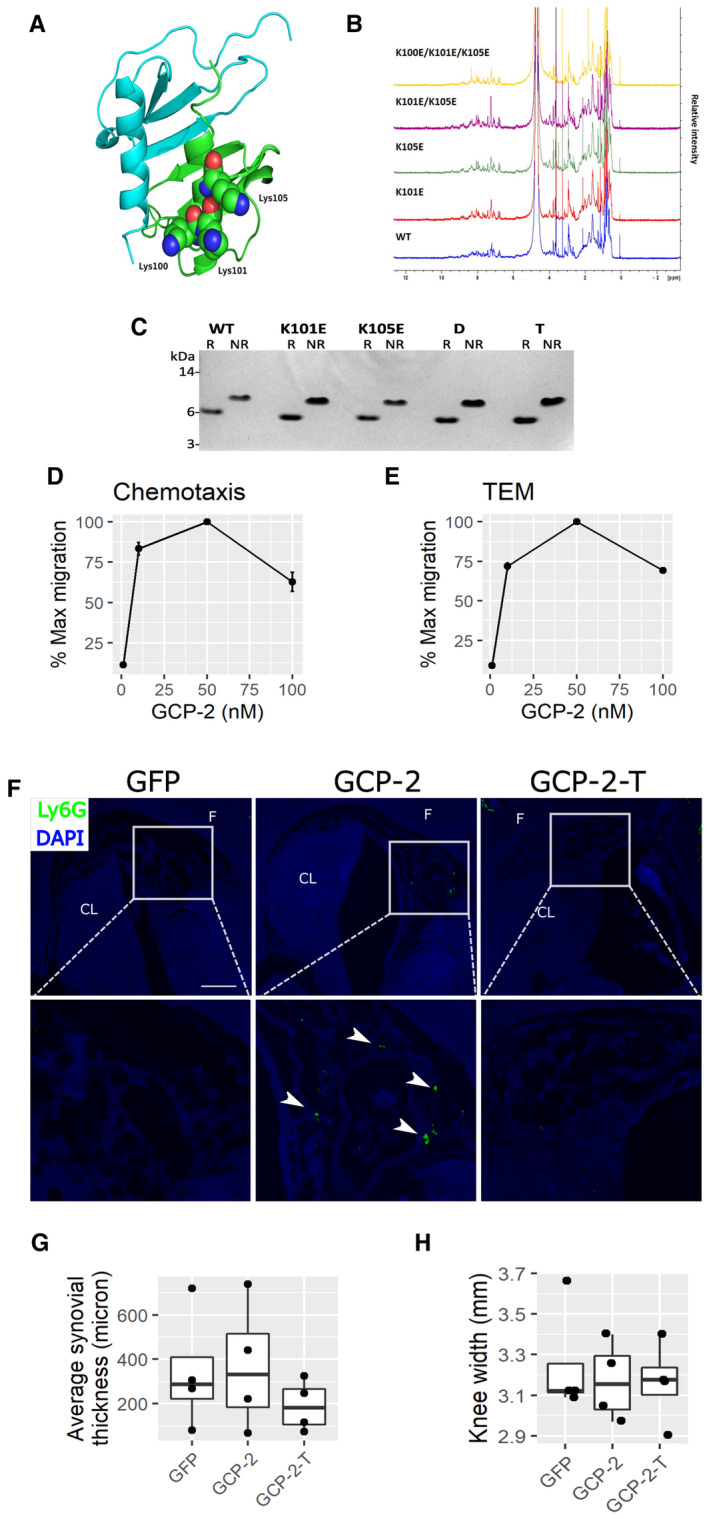
GCP‐2 model and characterization of WT and mutants A3D homology model of human GCP‐2 based on the NMR structure of human CXCL5 dimer (PDB: 2MGS) generated using SWISS‐MODEL. The GCP‐2 monomers are colored blue and green with Lys100, Lys101 and Lys105 shown in space filling representation for the latter.BComparison of 1D NMR spectra of WT and mutant GCP‐2 proteins (all human) made in this study.CSDS–PAGE analysis of GCP‐2 WT and mutants under reducing (R) and nonreducing (NR) conditions. Protein samples (1 μg) were either reduced and alkylated (R) or alkylated (NR). MW size from protein markers is displayed on the left. WT—wild‐type GCP‐2; K101E—K101E GCP‐2_single mutant; K105E—K105E GCP‐2_single mutant; D—K101E_K105E GCP‐2_double mutant; T—K100E_K101E_K105E GCP‐2_triple mutant.D, EDose‐dependency of WT GCP‐2‐induced (D) chemotaxis and (E) and transendothelial migration (TEM) of CXCR2‐expressing 300‐19 pre‐B cell line (mean values ± SEM). *N* = 4.FRepresentative images of immunostaining for the Ly6G neutrophil marker in the intercondylar notch of mice injected intra‐articularly with GFP, GCP‐2, or GCP‐2‐T adenovirus. Time point: 2 days. White arrowheads indicate neutrophils. Scale bar—50 μm. F—femur; CL—cruciate ligament.GAverage thickness of the synovial membrane 4 days after the intra‐articular injection of adenovirus encoding GFP, GCP‐2 or GCP‐2‐T. The thickness of the synovial membrane was assessed by histomorphometry using ImageJ. *P*‐values were determined with ANOVA *n* = 4 per group.HCaliper measurement of knee size 4 days after the injection of GFP, GCP‐2 and GCP‐2‐T adenovirus; *P*‐values were determined by one‐way ANOVA (*n* = 4). Source data are available online for this figure.

We hypothesized that disrupting the capacity of GCP‐2 to bind GAGs on the endothelial surface might render GCP‐2 unable to cause neutrophil chemotaxis while maintaining its capacity to activate its receptors on chondrocytes, and therefore retaining its chondrogenic activity. We predicted the position of the GAG‐binding site based on a multiple sequence alignment of related chemokines (Fig 4A) and identified a cluster of three highly conserved lysine residues (K100, K101 and K105) that could contribute to GAG binding (Vallet *et al*, 2021) (blue arrows in Fig 4A). We generated two single site mutants, K101E and K105E, respectively, a double mutant (GCP‐2‐D) that incorporates both mutations and a triple mutant (GCP‐2‐T) that contains an additional K100E mutation. Importantly, these mutations were not predicted to disrupt the tertiary structure of the protein as assessed by homology modeling of GCP‐2 (Fig EV2A), which was confirmed by the essentially identical 1D NMR spectra (Fig EV2B) and migration patterns on SDS–PAGE under reduced and nonreduced conditions (Fig EV2C) for wild‐type and mutant proteins; in the latter, the difference in apparent molecular weight of the reduced and nonreduced proteins is consistent with that reported for other chemokines, for example (Getschman *et al*, 2017).

The mutations resulted in a progressive decrease in binding to the GAG heparin, with the triple mutant showing the greatest impairment and weakest binding (Fig 4B and C). In dose–response curves, wild‐type (WT) GCP‐2 induced optimal chemotaxis and optimal transendothelial migration of a leukocyte cell line in Boyden chambers at a concentration of 50 nM (Fig EV2D and E). At the same concentration, both chemotaxis and endothelial migration were progressively affected by the mutations, with transendothelial migration reduced to a greater extent (Fig 4D and E).

Despite the loss of its chemoattractive properties, GCP‐2‐T was still able to induce AKT phosphorylation in chondrocytes (Fig 4F), and promoted chondrogenesis, as assessed by Alcian blue staining (Fig 4G) and aggrecan upregulation (Fig 4H). Under these experimental conditions, we could not detect statistically significant upregulation of COL2A1 by GCP‐2‐T (Fig 4I). Although GCP‐2 inhibited COL10A1 expression in osteogenic conditions (Fig 3E), neither GCP‐2 nor GCP‐2‐T affected baseline expression of COL10A1 (Fig 4J). To confirm that GCP‐2‐T reduced chemoattractive activity *in vivo*, within the joint environment, we intra‐articularly injected adenoviruses encoding wild‐type GCP‐2, GCP‐2‐T, or GFP as control. After 4 days, although both GCP‐2 and GCP‐2‐T induced AKT phosphorylation (Fig EV4A), only GCP‐2 induced accumulation of neutrophils within the joint space compared with GFP (Fig 4K—quantification—and Fig EV2F for representative images). No differences between the treatments were detected in overall synovial thickness (Fig EV2G) or knee swelling as measured using a caliper (Fig EV2H). Taken together, these data show that mutations in the GAG‐binding site of GCP‐2 disrupted its capacity to induce chemotaxis and transendothelial migration of leukocytes *in vitro* and *in vivo*, but not the ability to activate downstream signaling in chondrocytes.

### Exogenous GCP‐2‐T improves pain and structural outcomes in osteoarthritis

**Figure 5:**
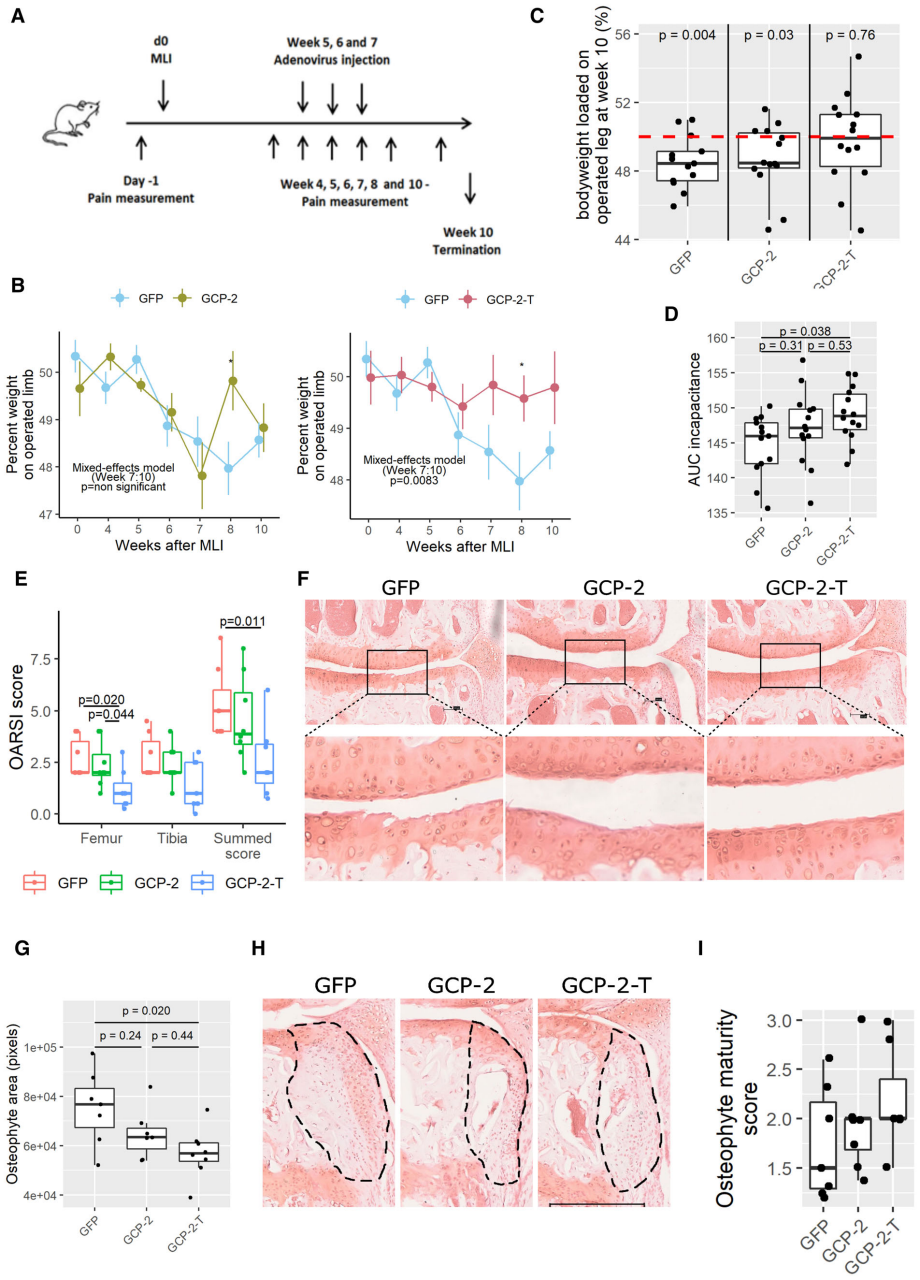
Exogenous GCP‐2‐T improves structural outcomes and pain in osteoarthritis ASchematic of *in vivo* experimental osteoarthritis.BWeekly pain measurement by incapacitance, shown as the percentage of bodyweight loaded on the operated leg in GFP (*n* = 15) versus GCP‐2 (*n* = 14) and GFP versus GCP‐2‐T (*n* = 15) treated mice. Circles show the mean, and error bars show 95% confidence intervals. *P*‐values were determined by building a mixed‐effect linear model. *P < 0.05.CIncapacitance at Week 10 (final time point); red line indicates 50% loading (no pain); *P*‐values were determined by unpaired, two‐tailed one sample Student's *t*‐test testing the hypothesis that mice loaded 50% body weight on the operated limb (*n* = 13 in the GFP group and *n* = 14 in the GCP‐2 and GCP‐2‐T groups).DThe area under the curve (AUC) of incapacitance calculated starting from Week 6; *P*‐values were determined by ANOVA with Tukey's HSD *post hoc* test (*n* = 13 in the GFP group and 14 in the GCP‐2 and GCP‐2‐T group).E, FOsteoarthritis severity assessed using (E) OARSI scoring system 10 weeks after MLI; GFP (*n* = 7), GCP‐2 (*n* = 8) and GCP‐2‐T (*n* = 7); *P*‐values were determined by fitting a generalized linear model followed by comparison of the estimated marginal means. F representative image (Safranin O staining) for each treatment; arrows indicate the tidemark; scale bar 100 μm.GQuantification of osteophytes area; GFP (*n* = 7), GCP‐2 (*n* = 7) and GCP‐2‐T (*n* = 8); *P*‐values were determined by ANOVA with Tukey's HSD *post hoc* test.HRepresentative images with osteophyte highlighted; scale bar 300 μm.IOsteophyte maturity score (*n* = 7); *P*‐values were determined by fitting a generalized linear model followed by pairwise comparison of the estimated marginal means. Source data are available online for this figure.

Having reduced the chemotactic function of GCP‐2 by impairing GAG binding, we investigated whether administration of GCP‐2‐T in a therapeutic regime could improve the outcome of instability‐induced osteoarthritis following menisco‐ligament injury (MLI) (Hamada *et al*, 2014; Thorup *et al*, 2020b). Mice received three weekly intra‐articular injections of adenoviruses encoding wild‐type murine GCP‐2, GCP‐2‐T, or GFP as control, starting 5 weeks after surgery, a time when cartilage lesions are well‐established (Kamekura *et al*, 2005; Fig 5A). All mice were killed 10 weeks after surgery. As expected (Thorup *et al*, 2020b), the control group developed pain on weight bearing (incapacitance) as measured by the percentage of body weight loaded on the operated limb from Week 6 (Fig 5B–D). Mice treated with GCP‐2‐T showed no pain, whereas wild‐type GCP‐2 did not significantly improve pain levels (Fig 5B–D).

**Figure EV3:**
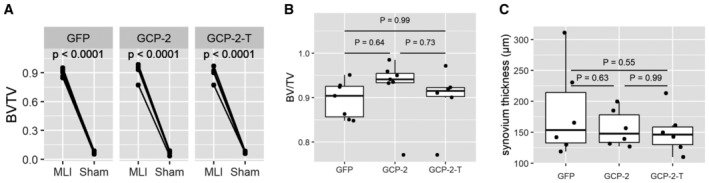
Exogenous GCP‐2‐T does not affect subchondral bone density and synovium thickness in osteoarthritis Bone density of subchondral bone in operated and sham‐operated knees as accessed by microCT as BV/TV; *P*‐values were determined by fitting a mixed‐effect model followed by pairwise comparison of the estimated marginal means (*n* = 14 for the GFP and GCP‐2 group and 12 for GCP‐2‐T).Density of subchondral bone in different treatments as accessed by microCT as BV/TV; GFP (*n* = 7), GCP‐2 (*n* = 7) and GCP‐2‐T (*n* = 6); *P*‐values were determined by ANOVA with Tukey's HSD *post hoc* test.Synovium thickness in μm assessed as average thickness of synovium in six areas of the joint; GFP (*n* = 7), GCP‐2 (*n* = 7) and GCP‐2‐T (*n* = 7); *P*‐values were determined by ANOVA with Tukey's HSD *post hoc* test. Source data are available online for this figure.

**Figure EV4:**
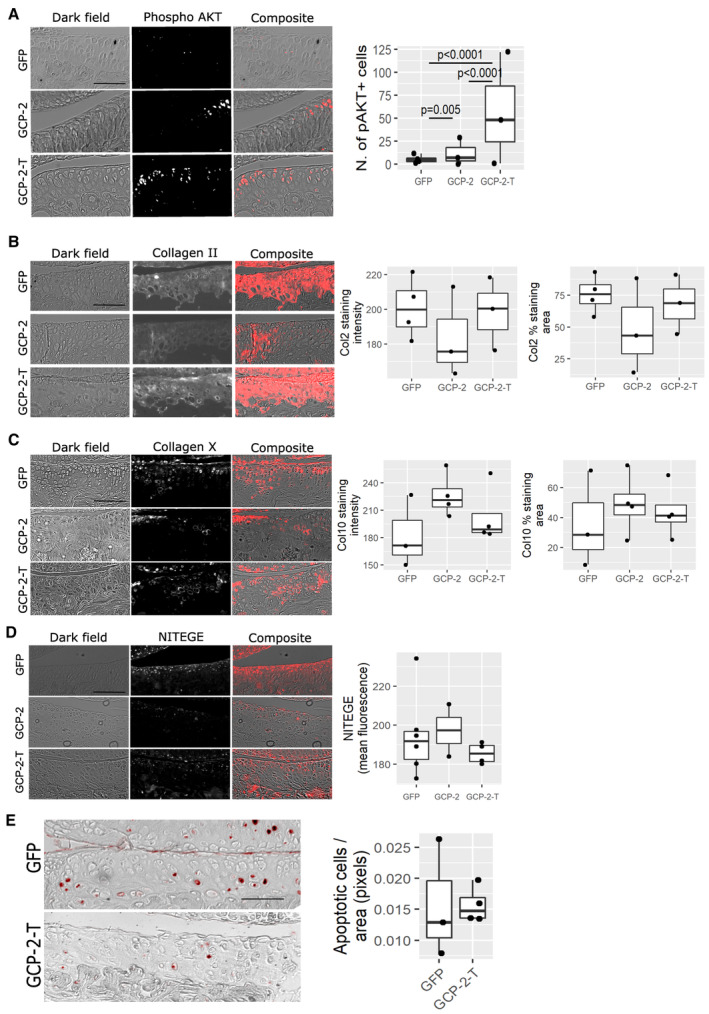
GCP‐2‐T activates AKT phosphorylation *in vivo*—molecular characterization of osteoarthritis in mice ATen‐week‐old female mice were injected intra‐articularly with 6 μl of GFP (*n* = 4), GCP‐2 (*n* = 3) or GCP‐2‐T (*n* = 3) adenovirus and killed 4 days later for immunofluorescence analysis of phospho‐AKT (pAKT). After thresholding, pAKT^+^ cells were counted using ImageJ. *P*‐values were obtained by fitting a generalized linear model (family = Poisson) followed by pairwise comparison of the estimated marginal means.B–ESections from the MLI experiment in Fig 5 were used to assess the expression of (B) collagen type II (Col2) (*n* = 4 for GFP and 3 for GCP‐2 and GCP‐2‐T), (C) collagen type X (Col10) (*n* = 3 for GFP and 4 for GCP‐2 and GCP‐2‐T), (D) NITEGE neo‐epitope (*n* = 6 for GFP, *n* = 2 for GCP‐2 and *n* = 4 for GCP‐2‐T) using immunofluorescence, and (E) apoptosis using the TUNEL assay (*n* = 3 for GFP and *n* = 4 for GCP‐2‐T); *P*‐values were obtained by fitting a generalized linear model. Whenever multiple technical replicates from the same knee were available, individual values were averaged. Scale bar = 50 μm. Source data are available online for this figure.

Reduced pain was accompanied by a reduced degree of structural damage in the medial compartment, with less cartilage loss in the GCP‐2‐T group, as assessed by OARSI score (Fig 5E and F), and reduced osteophyte size although not osteophyte maturity (Fig 5G–I). As anticipated, subchondral bone density increased in the osteoarthritic knees compared with the sham‐operated knees (Fig EV3A); however, no difference was observed between treatment groups (Fig EV3B). Reassuringly, no difference in synovial thickness was detected at this time point (Fig EV3C), suggesting no pro‐inflammatory effect of GCP‐2‐T. No statistically significant difference in the expression of collagen type II, collagen type X, NITEGE neo‐epitope and apoptosis was detected at this late time point (Fig EV4B–E).

## Discussion

Osteoarthritis is a major cause of pain and disability for which there are currently no disease‐modifying treatments available. We showed that by disrupting GAG binding, we could dissociate the anabolic function of GCP‐2 from its capacity to induce chemotaxis of inflammatory cells. Intra‐articular delivery of GAG binding‐deficient mutant GCP‐2‐T in therapeutic regime improved pain and prevented cartilage loss in experimental murine osteoarthritis.

Boosting articular cartilage anabolism has long been advocated as a strategy for treatment for osteoarthritis (Thorup *et al*, 2020a). Transforming Growth Factor (TGF)‐β and bone morphogenetic proteins (BMPs) are potent chondrogenic molecules, yet they have failed largely because they drive maturation of chondrocytes toward hypertrophy. Hypertrophic differentiation is a well‐established driver of osteoarthritis progression (Saito *et al*, 2010; Yang *et al*, 2010; Thorup *et al*, 2020b). Therefore, overactivation of TGF‐β signaling results in exacerbated osteoarthritis (Zhen *et al*, 2013), whereas suppression of TGF‐β protects from osteoarthritis progression (Zhen *et al*, 2013; Cui *et al*, 2016). Equally, BMP2 overexpression leads to ectopic bone formation (Blaney Davidson *et al*, 2014).

Not only were the anabolic properties of GCP‐2 not associated with hypertrophic differentiation, but—in fact—inhibited it. GCP‐2 supported the “stable articular cartilage phenotype,” which was shown to be associated with a favorable outcome in cartilage repair interventions such as autologous chondrocyte implantation (Dell'Accio *et al*, 2003b; Vanlauwe *et al*, 2012).

During embryonic development, GCP‐2 is expressed in the portion of the cartilage forming the skeletal elements, which resists endochondral bone formation and will form the permanent articular cartilage. While outside the scope of this work, it is reasonable to speculate that the embryonic expression of GCP‐2 in the prospective articular cartilage may contribute to its capacity to resist endochondral bone formation during development, whereas, in adult life, contributes to the prevention of mineralization and hypertrophic differentiation which are pathologic features in osteoarthritis (Saito *et al*, 2010).

We dissociated the chemotactic properties of GCP‐2 from its function as a chondrocyte differentiation factor by interfering with the binding to GAGs, which are present ubiquitously in the cartilage ECM (Fig 4). Binding to GAGs is believed to be essential for the formation of a chemokine haptotactic gradient on the surface of blood vessel endothelium, which activates neutrophil transendothelial migration (Rot, 1992; Middleton *et al*, 1997; Luster, 1998; Bizzarri *et al*, 2006; Proudfoot *et al*, 2017; Handel & Dyer, 2021). By disrupting the capacity of GCP‐2 to adhere to the endothelial surface, we disrupted its capacity to attract leukocytes (e.g. neutrophils), while retaining its capacity to activate chemokine receptors on chondrocytes. Additionally, the fact that GCP‐2‐T was more effective than wild‐type GCP‐2 in inducing AKT phosphorylation *in vivo* (Fig EV4A) but not *in vitro* (Fig 4F) suggests that disruption of GCP‐2 GAG binding within the cartilage ECM (Sherwood *et al*, 2015) might have increased its bioavailability for chondrocytes and therefore contributed to its efficacy. Within cartilage, ECM sequesters growth factors including FGF2 (Vincent *et al*, 2007), BMPs (Kawashima *et al*, 2020; Gerstner *et al*, 2021), and TGF‐β/CTGF (Tang *et al*, 2018). Loss of GAGs or loss of the interaction between the GAGs and growth factors resulted in growth factor release and in activation of the respective signaling pathways (Tang *et al*, 2018; Kawashima *et al*, 2020). As adenoviral GCP‐2 was not tagged, it was not possible to distinguish it from endogenous GCP‐2 and verify that adenoviral overexpression in murine osteoarthritis was at the same level for GCP‐2 and GCP‐2‐T; however, apart from the three mutated lysine residues, the adenoviral vectors were identical, produced and titrated at the same time, and the treatment was randomized within each cage. In addition, the outcomes of these *in vivo* studies paralleled that of our *in vitro* studies which were made with recombinant proteins at known concentration.

In our osteoarthritis model, we do not have an arm in which mice were killed before starting treatment, and therefore it is not possible to discriminate whether GCP‐2‐T caused cartilage regeneration or merely decreased breakdown. However, the fact that GCP‐2 induced extracellular matrix accumulation in human cartilage organoids implanted in nude mice (Fig 2F) suggests an anabolic function.

Despite the chondrogenic activity of GCP‐2‐T seen *in vitro*, 8 weeks after MLI we did not detect an increase in collagen type II staining. This may be because, in OA, the anabolic effect of GCP‐2‐T on collagen type II might have been masked by several confounders including increased collagen type II *de novo* synthesis associated with OA (Ijiri *et al*, 2008) or increased degradation by matrix metalloproteinases. Nevertheless, we believe that the anabolic activity of GCP‐2 on cartilage *in vivo* is demonstrated by its overexpression causing increased GAG and collagen type II content in the human cartilage organoids implanted in nude mice (Fig 2F and G). Notably, this adoptive model of cartilage formation is highly predictive of outcomes in clinical trials: For instance, chondrocyte biomarkers associated with this assay (Dell'Accio *et al*, 2001) predicted the clinical outcome of patients treated with autologous chondrocyte implantation at 3‐year follow‐up (Saris *et al*, 2009).

From a translational point of view, our data suggest that GCP‐2‐T can represent a first‐in‐kind disease‐modifying osteoarthritis drug. As osteoarthritis is a leading cause of permanent disability for which we do not yet have an effective pharmacological treatment, the use of GCP‐2‐T may represent a game changer in a high‐priority area of modern medicine.

Compared with other molecules which have shown some efficacy in osteoarthritis, GCP‐2‐T has the highly desirable feature of coupling disease modification (cartilage integrity) with rapid pain relief. This is important because cartilage integrity without pain relief is of little benefit to patients: For instance, FGF18 was effective in terms of cartilage integrity but failed to improve pain even after 5‐year follow‐up (Hochberg *et al*, 2019). Conversely, pain relief without cartilage protection is also unhelpful: tanezumab induced pain relief but resulted in dose‐dependent accelerated OA in some patients (Hochberg, 2015; Schnitzer *et al*, 2019), likely due to the increased use of the joint in the absence of chondroprotection.

It is still unclear how GCP‐2‐T mediates pain relief. It is possible that it may control local inflammation by competing with other inflammatory chemokines for the binding to CXCR2; however, at this stage, we cannot exclude that it may directly signal to local nociceptors within the joints. These studies are currently ongoing in our laboratory.

One limitation of this study is that we used adenoviral overexpression rather than a recombinant molecule to provide proof of concept of efficacy in osteoarthritis. The generation and validation of a recombinant GCP‐2‐T with a pharmacokinetic profile suitable for not‐too‐frequent intra‐articular injections is currently being pursued in our laboratory.

For its anabolic and possibly anti‐inflammatory properties, we anticipate that GCP‐2‐T may represent a novel therapeutic tool not only in osteoarthritis, but perhaps also in inflammatory arthritis where cartilage damage is the final disabling outcome.

## Materials and Methods

### Ethics

All animal procedures were subjected to and complied with local ethical approval and Home Office Licensing. Mouse experiments were regulated by Procedure Project License (PPL) nos. 70/7986 and 60/4528. Human samples were approved and obtained under the East London and the City Research Ethics Commitee3 (Ethics approval Rec N. 07/Q0605/29) and by the KULeuven Ethics Committee (OG032), application ML3356 and ML3359, by decision of September 8, 2004. The experiments conformed to the principles set out in the WMA Declaration of Helsinki and the Department of Health and Human Services Belmont Report. Adult human articular cartilage was obtained from patients undergoing joint replacement for knee OA after obtaining informed consent.

### Cells, cell culture, and Alcian blue staining

The murine pre‐B‐cell line 300‐19 expressing the CXCR2 receptor (clone 1D5, kind gift of Bernhard Moser; University of Cardiff, Cardiff, UK) (Loetscher *et al*, 1996) was expanded in RPMI‐1640 containing 10% heat inactivated fetal bovine serum (FBS), 2 mM glutamine, 1× penicillin–streptomycin, 50 μM ß‐mercaptoethanol, and 1.5 μg/ml of puromycin (Sigma). All other cells were expanded in Dulbecco Modified Eagle's Medium (DMEM) in the presence of antibiotics and antimycotics and 10% FBS at 37°C in a humidified atmosphere with 5% CO_2_. Mycoplasma tests were performed periodically. C28I/2 immortalized human chondrocytes were a kind gift of Professor Mary Goldring (HSSResearch Institute, Hospital for Special Surgery, New York, NY). C3H10T1/2 cells were purchased from ATCC. Primary human articular chondrocyte isolation and expansion was performed as previously described (Dell'Accio *et al*, 2001). Briefly, after obtaining informed consent, cartilage chips were dissected from the tibial plateau or femoral condyle discarded as surgical waste from patients undergoing knee replacement for osteoarthritis, minced and washed in phosphate buffered saline (PBS). Cartilage was then digested at 37°C in 0.2% crude type IV collagenase overnight, washed in PBS, and cultured as described above (Dell'Accio *et al*, 2001). Micromasses were plated as previously described (Eldridge *et al*, 2016; Thorup *et al*, 2020b); briefly, 20 μl of a 20 × 10^6^ cells/ml suspension was plated carefully in the center of an empty 24‐well cell culture plates and allowed to rest for 3 h in a humidified atmosphere with 5% CO_2_. The micromasses were then gently covered with 2 ml of culture medium for an additional 24 h. Following this, micromasses were switched to serum‐free medium and exposed to treatments after an additional 24 h. The detection of highly sulphated GAGs in the extracellular matrix was then assessed by Alcian blue staining at pH 0.2 as previously described (Eldridge *et al*, 2016; Thorup *et al*, 2020b). Alcian blue staining was quantified using densitometry with ImageJ (Rasband, 2011; Schneider *et al*, 2012), as previously described (De Bari *et al*, 2001a; Nalesso *et al*, 2011; Eldridge *et al*, 2020).

For gain‐of‐function experiments, GCP‐2 or vehicle control was added at a final concentration of 100 ng/ml, and the micromasses were cultured for an additional 3 days unless stated otherwise. For loss‐of‐function experiments, densitometry quantification of Alcian blue was made for: HAC micromasses stimulated with 12.5 μg/ml GCP‐2 blocking antibody (Bio‐Techne MAB333) or IgG control (MAB002); C3H10T½ cells (ATCC CCL‐226) and C28/I2 cells were transfected with 25 nM of GCP‐2 siRNA (Thermofisher Silencer™ Select Pre‐Designed siRNA Catalog# 4392420) for 24 h and cultured in micromass. siRNA sequences: sense CUAUUGUAUUUCUAUCAUATT; antisense UAUGAUAGAAAUACAAUAGTT.

### Hypertrophic and osteogenic differentiation

Hypertrophy was induced in HACs as previously described (Robson *et al*, 2000) using thyroid hormone. Osteogenic differentiation was induced in C3H10T½ cells as previously described using an osteogenic medium containing β‐glycerophosphate, dexamethasone, and ascorbic acid (Dell'Accio *et al*, 2003a). After 3 or 4 weeks (hypertophic or osteogenic differentiation, respectively), cells were harvested for real‐time PCR gene expression analysis or fixed, washed in water, and stained for alkaline phosphatase (Vector Red) or alizarin red S solution (2% aqueous solution [pH 4.2]; Sigma) for 3 min, as previously described (De Bari *et al*, 2001b; Eldridge *et al*, 2020).

### Total RNA extraction and real‐time PCR

RNA extraction was performed using Trizol® (Invitrogen) according to the manufacturer's instruction. Reverse transcription and real‐time PCR were performed according to standard protocols as previously described (Eldridge *et al*, 2016; Thorup *et al*, 2020b). Primers are listed in Table 1.

**Table 1:** List of primers.

Primer name	Primer sequence
Human and Mouse B Actin Forward	TGACGGGGTCACCCACACTGTGCCCATCTA
Human and Mouse B Actin Reverse	CTAGAAGCATTTGCGGTGGACGATGGAGGG
Mouse Aggrecan Forward	GTTGTCATCAGCACCAGCATC
Mouse Aggrecan Reverse	ACCACACAGTCCTCTCCAGC
Human RUNX2 Forward	CGGACATACCGAGGGACATG
Human RUNX2 Reverse	CCAACCCACGAATGCACTATC
Human Col10A1 Forward	AATCCCTGGACCGGCTGGAATTTC
Human Col10A1 Reverse	TTGATGCCTGGCTGTCCTGGAACC
Human Col1A1 Forward	CGTGGTGACAAGGGTGAGAC
Human Col1A1 Reverse	TAGGTGATGTTCTGGGAGGC

### Western blot analysis and immunostaining

Western blots were carried out as described previously (Sherwood *et al*, 2015). Membranes were blocked and antibodies diluted in 1% (w/v) BSA + 0.01% (v/v) Tween 20 in TBS. Primary antibodies are listed in Table 2. Immunofluorescence staining was carried out according to standard protocols as previously described (Nalesso *et al*, 2017). Antigen retrieval methods for each staining are listed in Table 2. For Ly6G immunostaining Tyramide signal amplification (TSA) was used using the ABC kit (Vector Laboratories).

**Table 2:** Antibodies and working concentrations.

Antibody	Dilution	Supplier	Antigen retrieval (used for immunohistochemistry)
β Actin	1:10,000	Abcam	
Akt	1:1,000	CST	
pAkt	1:1,000	CST	
Collagen II	1:500	Millipore	
GFP	1:100	CST	
GCP‐2	1:100	R&D Systems	
GCP‐2	1:100	Byorbit	
Lix	1:100	R&D Systems	
His	1:400	CST	
Collagen II	1:500	Millipore	Pepsin digestion
Collagen X	1:100	Ebioscience	Pepsin digestion
MMP‐13	1:100	Abcam	Citrate‐EDTA buffer (pH 6.2)
GCP‐2	1:200	Byorbit	Pepsin digestion
NITEGE	1:50	MD Bioproducts	Proteinase K
pAkt	1:200	CST	Pepsin digestion
Ly6G	1:100	Biolegend	Citrate Buffer
TUNEL assay		Sigma‐Aldrich	Proteinase K

### Generation of recombinant GCP‐2 and mutants

The cDNA of human WT GCP‐2 (accession number NM_002993 bases 185‐415) or the GCP‐2 mutants were cloned into the pRK172 T7 expression vector (by GenScript, USA); an N‐terminal 6xHis tag was included followed by a factor Xa cleavage site in order to allow production of the native N‐terminus. K101E and K105E single mutants and a double mutant (GCP‐2‐D) incorporating both mutations, and a triple mutant (GCP‐2‐T) containing an additional mutation K100E were generated.

Wild‐type and mutant plasmids were transformed into SHuffle T7 Express lysY Competent *Escherichia coli* cells (NEB, USA) and recombinant protein expressed at 16°C and purified using standard methodology. Briefly, following IPTG induction, cells were harvested and resuspended in IMAC buffer (20 mM sodium phosphate, 500 mM NaCl, pH 7.4) supplemented with protease inhibitor cocktail (Sigma‐Aldrich) and lysed by sonication. Supernatants were collected by centrifugation and loaded onto a 1‐ml HisTrap HP column (GE Healthcare) and protein eluted with a 0–500 mM gradient of imidazole in IMAC buffer; GCP‐2 proteins were pooled and dialyzed against 20 mM Tris–HCl pH7.4, 100 mM NaCl. CaCl_2_ was added (2 mM final concentration) and the 6xHis tag cleaved by factor Xa (overnight) according to the manufacturer's instructions (Millipore, USA) and removed by retention on the HisTrap HP column (run in 20 mM Tris–HCl pH 7.0, 100 mM NaCl). The GCP‐2 proteins were loaded onto a 1 ml HiTrap Heparin HP column equilibrated with 20 mM Tris–HCl pH 7.0, 100 mM NaCl and eluted with a gradient 0.1–1.5 M NaCl in 20 mM Tris–HCl pH 7.0. Fractions were pooled, and the protein was snap‐frozen and stored at −20°C. The WT and mutant GCP‐2 proteins (0.3 mg/ml) were analyzed by one‐dimensional ^1^H nuclear magnetic resonance (NMR) spectroscopy on a 500 MHz Bruker NMR spectrometer in 20 mM sodium phosphate, 100 mM NaCl, pH 7.0, 10% (v/v) D_2_O. This confirmed that the mutations did not have any deleterious effects on the GCP‐2 structure, and all mutants had a WT fold. For SDS–PAGE analysis under reducing conditions, protein samples (1 μg) were incubated in 100 mM Tris–HCl, pH 8.0, 20 mM DTT, 4 M Urea and 1% (w/v) SDS for 30 min at 37°C and then alkylated by the addition of iodoacetamide to 40 mM and incubated at 37°C for 5 min, or under nonreducing conditions, samples were incubated in 100 mM Tris–HCl, pH 8.0, 40 mM iodoacetamide, 4 M Urea, and 1% (w/v) SDS for 5 min at 37°C. The protein samples were run on a 4–12% Bis‐Tris SDS–PAGE gel (Invitrogen, UK) and then stained with Ready Blue Protein Gel Stain (Merck, UK).

### Heparin‐binding assays

The heparin‐binding activities of WT and mutant GCP‐2 were compared using a microtiter plate assay, carried out as described previously (Dyer *et al*, 2014).

The relative heparin‐binding affinities of GCP‐2 WT and mutants were also assessed by affinity chromatography on a HiTrap Heparin HP 1 ml column (GE Healthcare) as described previously (Clark *et al*, 2013). Briefly, GCP‐2 or mutants (50 μg) were loaded onto the column equilibrated in PBS, pH 7.3, and the proteins were eluted using a linear gradient of 0–2 M NaCl in the same buffer and absorbance monitored at 215 nm.

### Chemotaxis and transendothelial migration assay

The chemotaxis and transendothelial migration assays were performed as described (Dyer *et al*, 2014). Chemotaxis was assessed using 6.5 mm Transwell permeable supports in 24‐well plates (5 μm pore polycarbonate membrane; Corning) with CXCR2‐expressing 300‐19 pre‐B cells. For transendothelial migration assays, 5 × 10^4^ EA.hy 926 cells were seeded on 6.5 mm Transwell inserts. Monolayer formation was confirmed by microscopy.

To assess *in vivo* neutrophil migration, 8‐week‐old female C57BL/6 mice (purchased from Charles River UK) received intra‐articular injections of 10 μl 10^9^ PFU of adenoviral constructs and culled 48 h later. Mice were maintained in standard housing in groups of six and fed *ad libitum*, and the investigator was blinded to the treatment being injected. The infiltration of neutrophils into the knee joint was evaluated using immunofluorescence staining for Ly6G marker. The number of neutrophils was counted in intercondylar area.

### *In vivo* ectopic cartilage formation assay

The ectopic cartilage (EC) formation assay was modified from previously described protocols (Dell'Accio *et al*, 2001; Eldridge *et al*, 2016; Thorup *et al*, 2020b, 2022). For each injection, 1 × 10^6^ chondrocytes were mixed with 1 × 10^5^ COS7 cells (a gift from Michael Ferns [UC Davis Healthsystem, USA]) transfected with GFP or GCP‐2 plasmids and growth arrested with mitomycin C, as described in (Eldridge *et al*, 2016). The cell mixture was resuspended in 100 μl rat type I collagen (Corning) at pH 7.2–7.6 and injected subcutaneously in the back of 3‐week‐old female CD1‐Foxn1^nu^ mice. Mice were maintained in standard housing in groups of six and fed *ad libitum*. After 2 weeks, mice were sacrificed, and cartilage organoids were retrieved. Harvested organoids were processed for toluidine blue staining and the area stained metachromatically was analyzed using ImageJ as previously described (Eldridge *et al*, 2016; Thorup *et al*, 2020b).

### Adenoviruses

The cDNA encoding the mature region of mouse GCP2 (genebank NM_009141.3) or the triple mutant version or GFP were cloned downstream of a Igk signal peptide and followed by a stop codon into Ad5 adenovirus backbone. All adenoviruses were constructed and packaged at Vectorbuilder.

### Experimental osteoarthritis in mice

Osteoarthritis was induced in 10‐week‐old male C57BL/6 mice (purchased from Charles River UK) by meniscus‐ligament injury (MLI) as previously described (Hamada *et al*, 2014; Thorup *et al*, 2020b)—45 animals in total. Briefly, under general anesthesia, after medial arthrotomy, the medial collateral ligament was resected, the anterior portion of the medial meniscus removed, and the joint capsule and skin were sutured in separate layers. Contralateral knees were sham‐operated: The joint capsule was opened, but the medial collateral ligament and the medial meniscus were left intact. Within each cage, animals were block‐randomized to receive three weekly intra‐articular injections of 10 μl 10^9^ PFU of adenovirus encoding mouse GFP or GCP‐2 or GCP‐2‐T starting from Week 5 (Fig 5A) within the operated knee. *n* = 15 animals were used per treatment group, where sample size was based on power calculation of a previous study using this model (Thorup *et al*, 2020b). All mice were maintained in standard housing in groups of six and fed *ad libitum*. Investigators performing the surgery, the injection, the analysis of pain and the histological scoring were blinded to the treatment. Two mice in the GCP‐2‐T group were excluded from downstream analysis due to an accidental breach of protocol.

### Incapacitance

Incapacitance was measured throughout the study using a Linton incapacitance meter (Thorup *et al*, 2020b). Measurements were taken over 3 s, and 10 readings per mouse were taken at each time point, and mean incapacitance was calculated. The area under the curve for each mouse was calculated from weeks 6 to 10 after surgery.

### Osteoarthritis scoring

OARSI scoring (Glasson *et al*, 2010) was performed as previously described (Thorup *et al*, 2020b) on Safranin O stained sections. Sections within the load‐bearing area (200 μm from the section through the middle of the tibial plateau) were collected at an 80 μm interval and stained with 0.1% Safranin‐O pH 5. Sections with cutting or staining artifacts were discarded. All images were taken using the same settings on NanoZoomer S60 slide scanner microscope and osteoarthritis severity in the medial compartment was assessed using the OARSI score (Glasson *et al*, 2010). Sections were scored independently by two investigators (FD and SC) who were blind to the treatment and the individual scores were averaged. The area and density of osteophytes were measured in a similar way, by manually selecting the region of interest using ImageJ. The size and morphology of osteophytes were scored using the scoring system from (Little *et al*, 2009). Knees with <3 optimal sections for assessment of osteoarthritis severity were excluded from analysis.

### Micro‐CT

Micro‐CT analyses were performed using a Skyscan 1,176 microtomograph (Bruker) at 9 μm resolution through a 0.5 mm aluminum filter. Images were analyzed with Image J using Bone J software (Doube *et al*, 2010) for ratio of bone volume to total volume (BV/TV) in regions of interest (ROI) of the medial tibial epiphyseal bone fraction.

### Image treatment

Within each experiment, images were obtained using the same setting and without autogain. Where needed, images have been edited for best rendering (contrast, brightness, and hue) using the same parameters within each experiment so not to alter the differences between samples and experimental groups.

### Statistical analysis

Boxplots indicate the minimum value (lower whisker), the 1st quartile (lower hinge), median (middle line), 3rd quartile (upper hinge), and maximum value (upper whisker). Data shown in line graphs represent mean values ± standard error of the means. Technical replicates such as PCR runs of the same samples or staining of sections from the same knee were averaged before analysis if interval in nature, or the median was used instead for ordinal or interval data such as the OARSI score. All individual data shown represent biological replicates. Samples with artifacts (cutting/imaging or staining artifacts for images or PCR data with suboptimal melting curves) were removed from analysis. Parametric data were compared with Student's *t*‐test or analysis of variance (ANOVA) followed by Tukey's honest significant difference (HSD) *post hoc* test for multiple comparisons. Data transformation was applied to satisfy the assumption of the *t*‐test and ANOVA, when applicable as described (Chambers *et al*, 1983). When the assumptions for ANOVA were not fulfilled, means were compared by fitting generalized linear models followed by pairwise comparison of the estimated marginal means in the treatment groups. Tests were two‐sided and *P*‐values < 0.05 were considered significant. Individual tests used and specific *P*‐values are indicated in figure legends. Raw data and R scripts to reproduce statistical analyses and graphs are downloadable as supplementary materials.

## Author contributions

**Sara Caxaria:** Data curation; formal analysis; supervision; validation; methodology; writing – original draft. **Nikolaos Kouvatsos:** Data curation; formal analysis; validation; investigation; visualization; methodology; writing – original draft; writing – review and editing. **Suzanne E Eldridge:** Conceptualization; data curation; formal analysis; supervision; funding acquisition; validation; investigation; visualization; methodology; writing – original draft; writing – review and editing. **Mario Alvarez-Fallas:** Investigation. **Anne‐Sophie Thorup:** Resources; data curation; formal analysis; supervision; methodology; writing – original draft; writing – review and editing. **Daniela Cici:** Data curation; validation; investigation; visualization; writing – review and editing. **Aida Barawi:** Investigation. **Ammaarah Arshed:** Investigation. **Danielle Strachan:** Data curation; formal analysis; investigation. **Giulia Carletti:** Resources; data curation; formal analysis; supervision; investigation; visualization; writing – original draft; writing – review and editing. **Xinying Huang:** Investigation. **Sabah Bharde:** Investigation. **Melody Deniz:** Resources; formal analysis; investigation. **Jacob Wilson:** Data curation; investigation. **Bethan L Thomas:** Data curation; formal analysis; supervision; investigation. **Costantino Pitzalis:** Conceptualization; resources; supervision. **Francesco Paolo Cantatore:** Conceptualization; supervision. **Manasi Sayilekshmy:** Supervision; investigation. **Shafaq Sikandar:** Supervision; methodology; writing – review and editing. **Frank P Luyten:** Conceptualization; supervision; funding acquisition; writing – original draft. **Thomas Pap:** Supervision; funding acquisition. **Joanna C Sherwood:** Conceptualization; supervision; funding acquisition; investigation; methodology; writing – original draft; writing – review and editing. **Anthony J Day:** Conceptualization; resources; data curation; formal analysis; supervision; funding acquisition; methodology; writing – original draft; writing – review and editing. **Francesco Dell'Accio:** Conceptualization; resources; data curation; formal analysis; supervision; funding acquisition; investigation; methodology; writing – original draft; project administration; writing – review and editing.

## Disclosure and competing interests statement

FD, SC, JCS, NK and AJD are co‐inventors on a patent application submitted by QMUL that covers the subject of this work. AJD and NK are shareholders of Link Biologics Ltd (of which AJD is a cofounder) that is developing a therapy for osteoarthritis unconnected with the work described here.

## Supporting information



Expanded View Figures PDF

Source Data for Expanded View

PDF+

Source Data for Figure 1

Source Data for Figure 2

Source Data for Figure 3

Source Data for Figure 4

Source Data for Figure 5

## Data Availability

This study includes no data deposited in external repositories.
