# Supplementary material for: Disease modification and symptom relief in osteoarthritis using a mutated GCP‐2/CXCL6 chemokine
Source: EMBO Mol Med. 2022 Dec 12;15(1):e16218. doi: 10.15252/emmm.202216218 (PMC9832835; doi:10.15252/emmm.202216218)
Supplement: Supplementary file 1 — Expanded View Figures PDF [file EMMM-15-e16218-s002.pdf]

Expanded View Figures

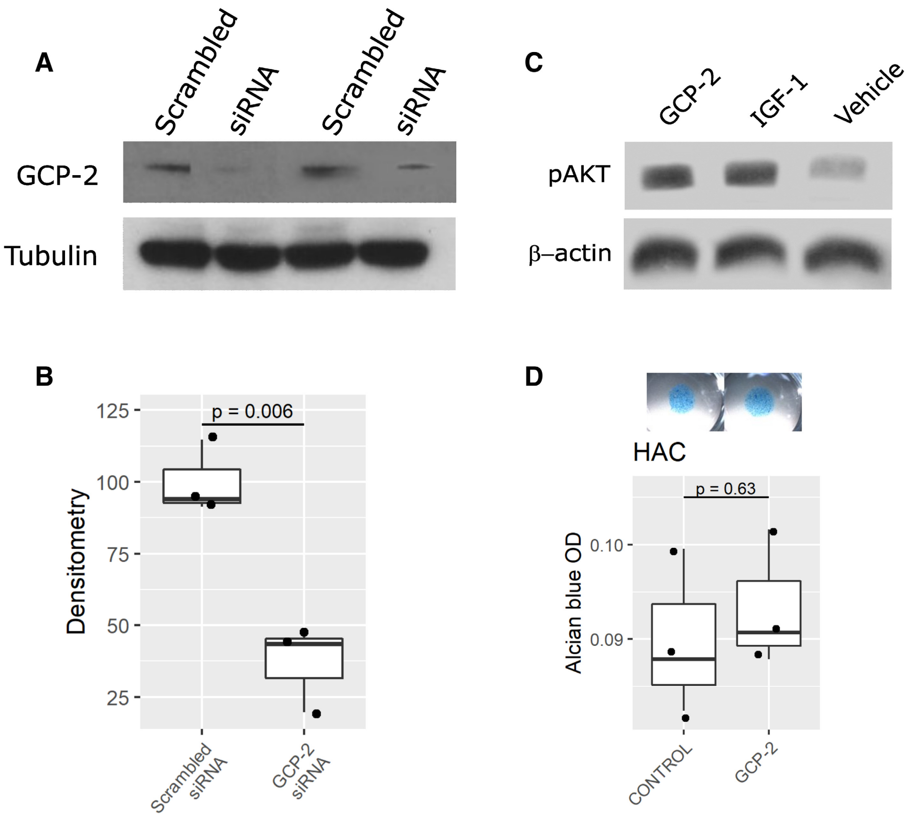

Figure EV1. siRNA validation *in vitro*. Effect of GCP-2 on AKT phosphorylation in chondrocytes and on GAG production in HACs.

A, B C28/I2 (A) or C3H10T1/2 (B) monolayers transfected with 25 nM of either scrambled or GCP-2 siRNA for 72 h and analyzed for GCP-2 levels by Western blotting. (A) A representative western blot; (B) densitometric quantification of three independent experiments. $N = 3$. *P*-values were determined using the two-tailed Student's *t*-test.

C C28/I2 micromasses stimulated for 3 days with recombinant GCP-2, IGF-1 (positive control) or untreated (vehicle) and assessed for AKT phosphorylation levels by Western blotting; β-actin as loading control; GCP-2—WT, GCP-2-treated samples, IGF-1—IGF-1-treated samples, V—vehicle-treated samples, pAKT—phosphorylated AKT.

D Alcian blue staining and spectrophotometric quantitation of GAGs in micromasses of HACs treated with recombinant GCP-2 or vehicle ($n = 3$); *P*-values were determined by unpaired, two-tailed Student's *t*-test. OD—optical density.

Source data are available online for this figure.

© 2022 The Authors

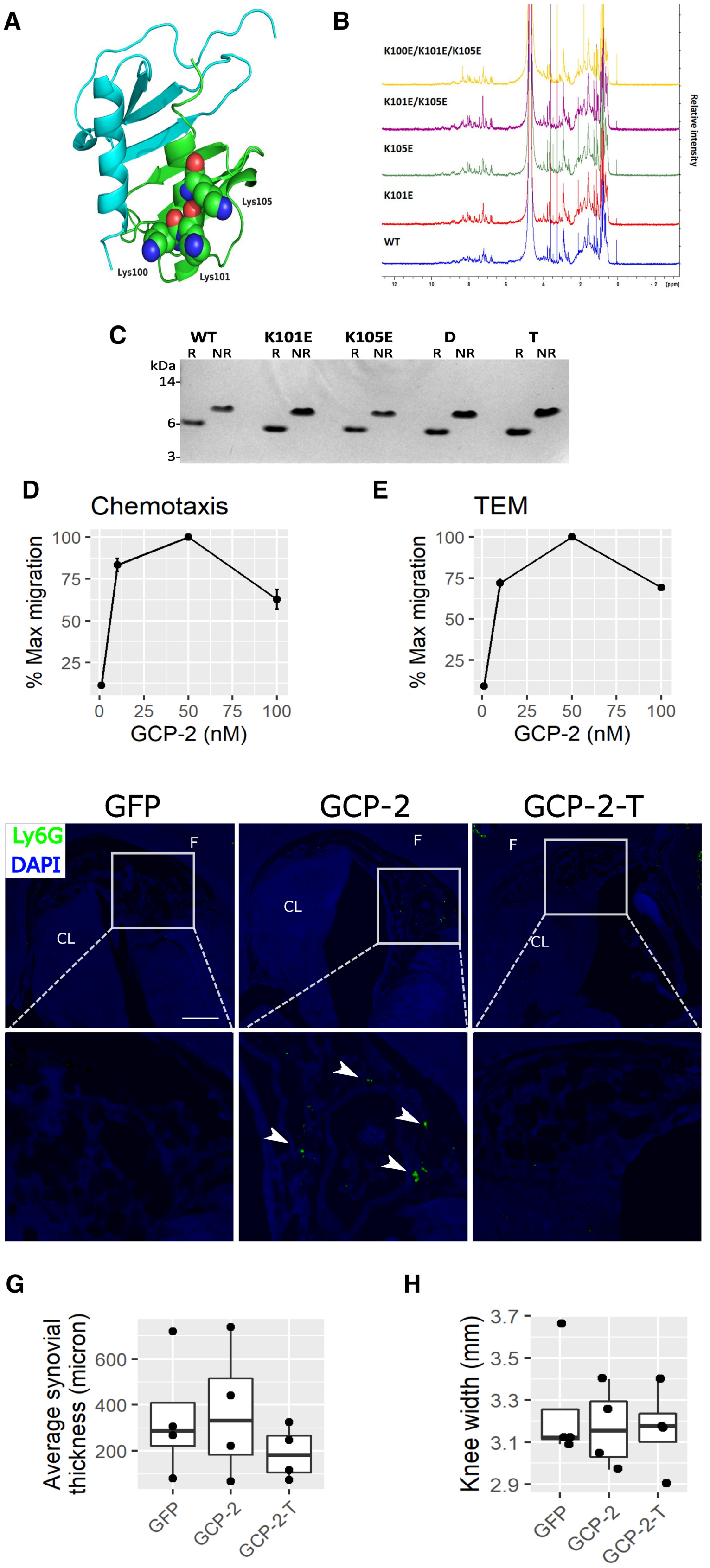

Figure EV2. GCP-2 model and characterization of WT and mutants.

A 3D homology model of human GCP-2 based on the NMR structure of human CXCL5 dimer (PDB: 2MGS) generated using SWISS-MODEL. The GCP-2 monomers are colored blue and green with Lys100, Lys101 and Lys105 shown in space filling representation for the latter.

B Comparison of 1D NMR spectra of WT and mutant GCP-2 proteins (all human) made in this study.

C SDS–PAGE analysis of GCP-2 WT and mutants under reducing (R) and nonreducing (NR) conditions. Protein samples (1 μg) were either reduced and alkylated (R) or alkylated (NR). MW size from protein markers is displayed on the left. WT—wild-type GCP-2; K101E—K101E GCP-2_single mutant; K105E—K105E GCP-2_single mutant; D—K101E_K105E GCP-2_double mutant; T—K100E_K101E_K105E GCP-2_triple mutant.

D, E Dose-dependency of WT GCP-2-induced (D) chemotaxis and (E) and transendothelial migration (TEM) of CXCR2-expressing 300-19 pre-B cell line (mean values ± SEM). $N = 4$.

F Representative images of immunostaining for the Ly6G neutrophil marker in the intercondylar notch of mice injected intra-articularly with GFP, GCP-2, or GCP-2-T adenovirus. Time point: 2 days. White arrowheads indicate neutrophils. Scale bar—50 μm. F—femur; CL—cruciate ligament.

G Average thickness of the synovial membrane 4 days after the intra-articular injection of adenovirus encoding GFP, GCP-2 or GCP-2-T. The thickness of the synovial membrane was assessed by histomorphometry using ImageJ. *P*-values were determined with ANOVA $n = 4$ per group.

H Caliper measurement of knee size 4 days after the injection of GFP, GCP-2 and GCP-2-T adenovirus; *P*-values were determined by one-way ANOVA ($n = 4$).

Source data are available online for this figure.

 © 2022 The Authors

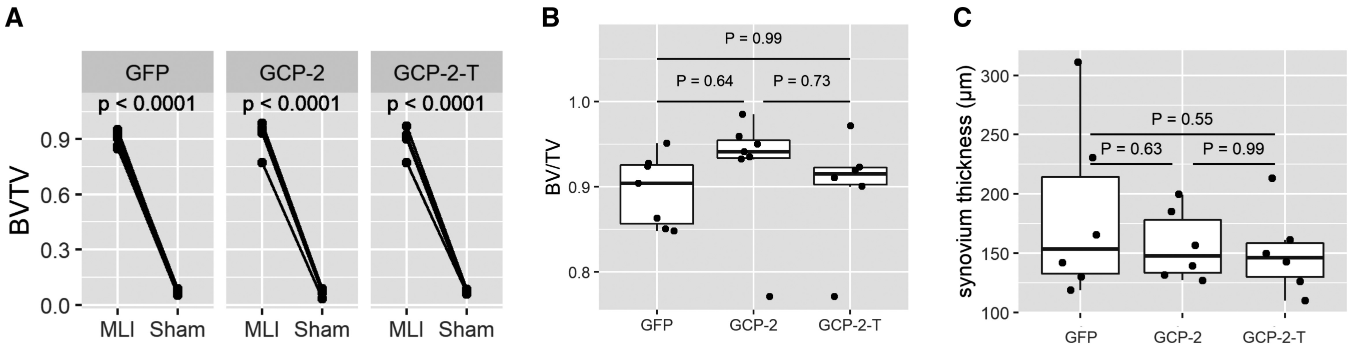

Figure EV3. Exogenous GCP-2-T does not affect subchondral bone density and synovium thickness in osteoarthritis.

A Bone density of subchondral bone in operated and sham-operated knees as accessed by microCT as BV/TV; *P*-values were determined by fitting a mixed-effect model followed by pairwise comparison of the estimated marginal means (n = 14 for the GFP and GCP-2 group and 12 for GCP-2-T).

B Density of subchondral bone in different treatments as accessed by microCT as BV/TV; GFP (n = 7), GCP-2 (n = 7) and GCP-2-T (n = 6); *P*-values were determined by ANOVA with Tukey's HSD *post hoc* test.

C Synovium thickness in µm assessed as average thickness of synovium in six areas of the joint; GFP (n = 7), GCP-2 (n = 7) and GCP-2-T (n = 7); *P*-values were determined by ANOVA with Tukey's HSD *post hoc* test.

Source data are available online for this figure.

Figure EV4. GCP-2-T activates AKT phosphorylation *in vivo*—molecular characterization of osteoarthritis in mice.

A Ten-week-old female mice were injected intra-articularly with 6 µl of GFP (n = 4), GCP-2 (n = 3) or GCP-2-T (n = 3) adenovirus and killed 4 days later for immunofluorescence analysis of phospho-AKT (pAKT). After thresholding, $pAKT^+$ cells were counted using ImageJ. *P*-values were obtained by fitting a generalized linear model (family = Poisson) followed by pairwise comparison of the estimated marginal means.

B–E Sections from the MLI experiment in Fig 5 were used to assess the expression of (B) collagen type II (Col2) (n = 4 for GFP and 3 for GCP-2 and GCP-2-T), (C) collagen type X (Col10) (n = 3 for GFP and 4 for GCP-2 and GCP-2-T), (D) NITEGE neo-epitope (n = 6 for GFP, n = 2 for GCP-2 and n = 4 for GCP-2-T) using immunofluorescence, and (E) apoptosis using the TUNEL assay (n = 3 for GFP and n = 4 for GCP-2-T); *P*-values were obtained by fitting a generalized linear model. Whenever multiple technical replicates from the same knee were available, individual values were averaged. Scale bar = 50 µm.

Source data are available online for this figure.

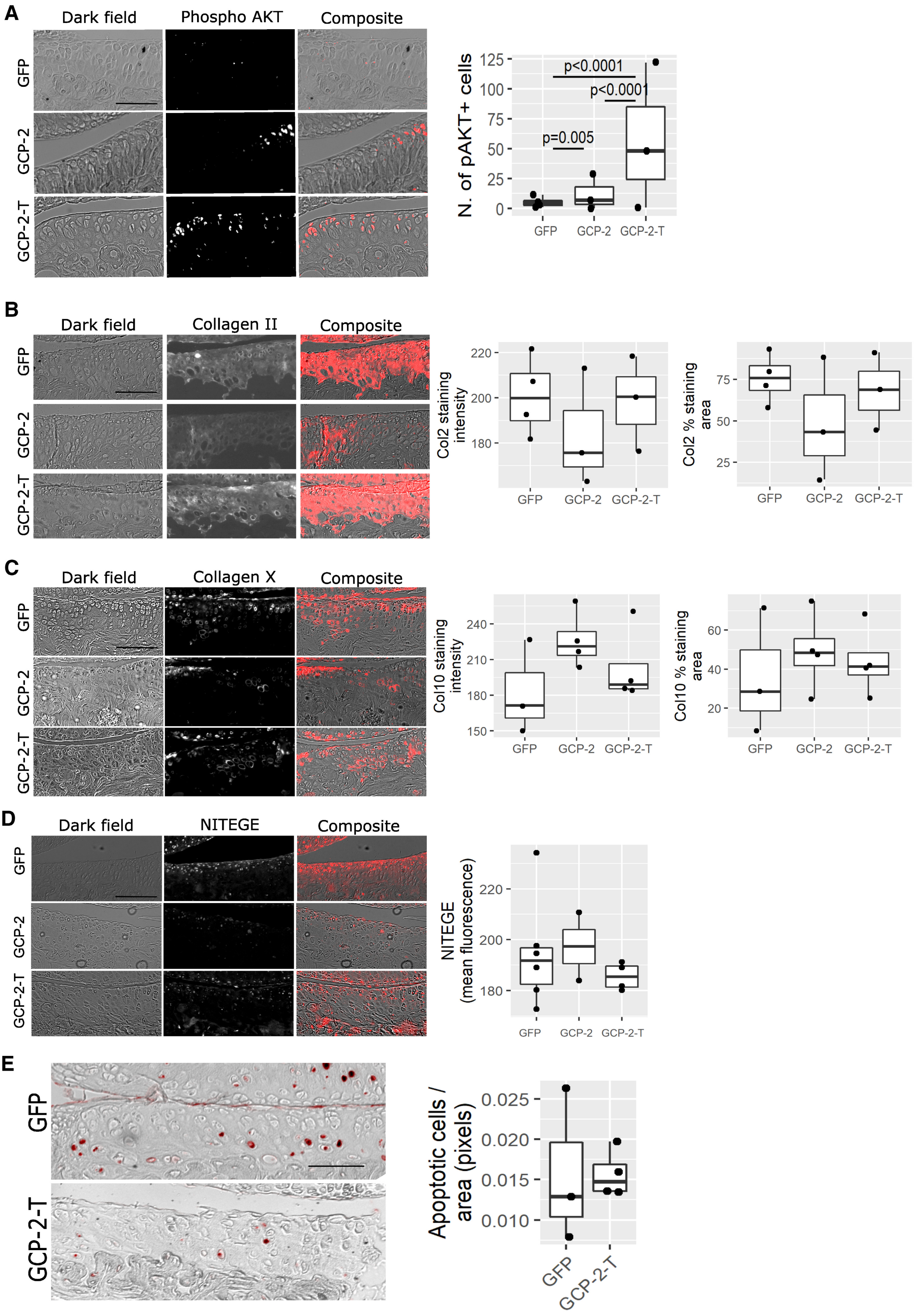

Figure EV4.

 © 2022 The Authors
