# Supplementary figures and images for: Disease modification and symptom relief in osteoarthritis using a mutated GCP‐2/CXCL6 chemokine
Source: EMBO Mol Med. 2022 Dec 12;15(1):e16218. doi: 10.15252/emmm.202216218 (PMC9832835; doi:10.15252/emmm.202216218)

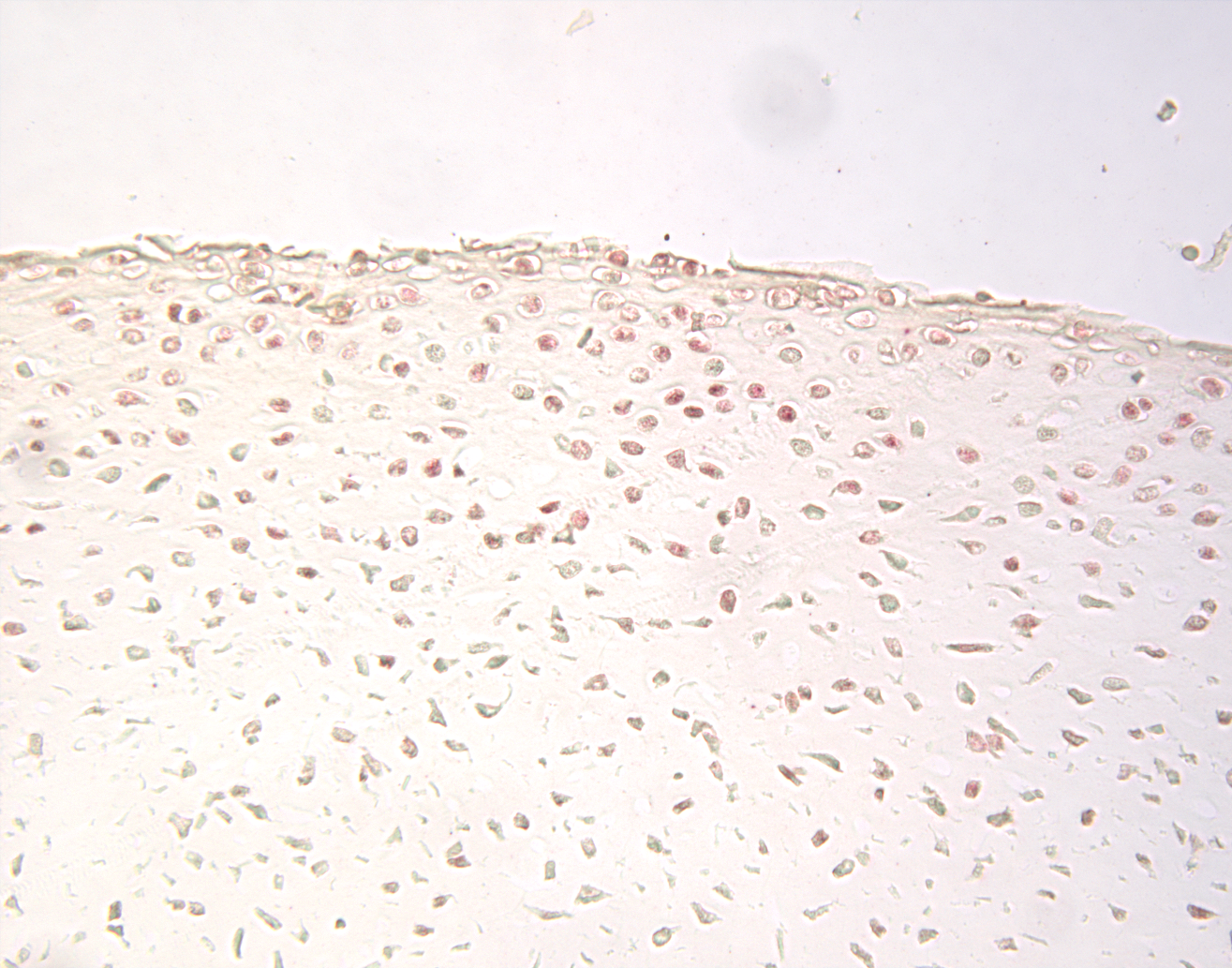

Supplement: Supplementary file 4 — Source Data for Figure 1 [file EMMM-15-e16218-s008.zip › F1/021015c.tif]

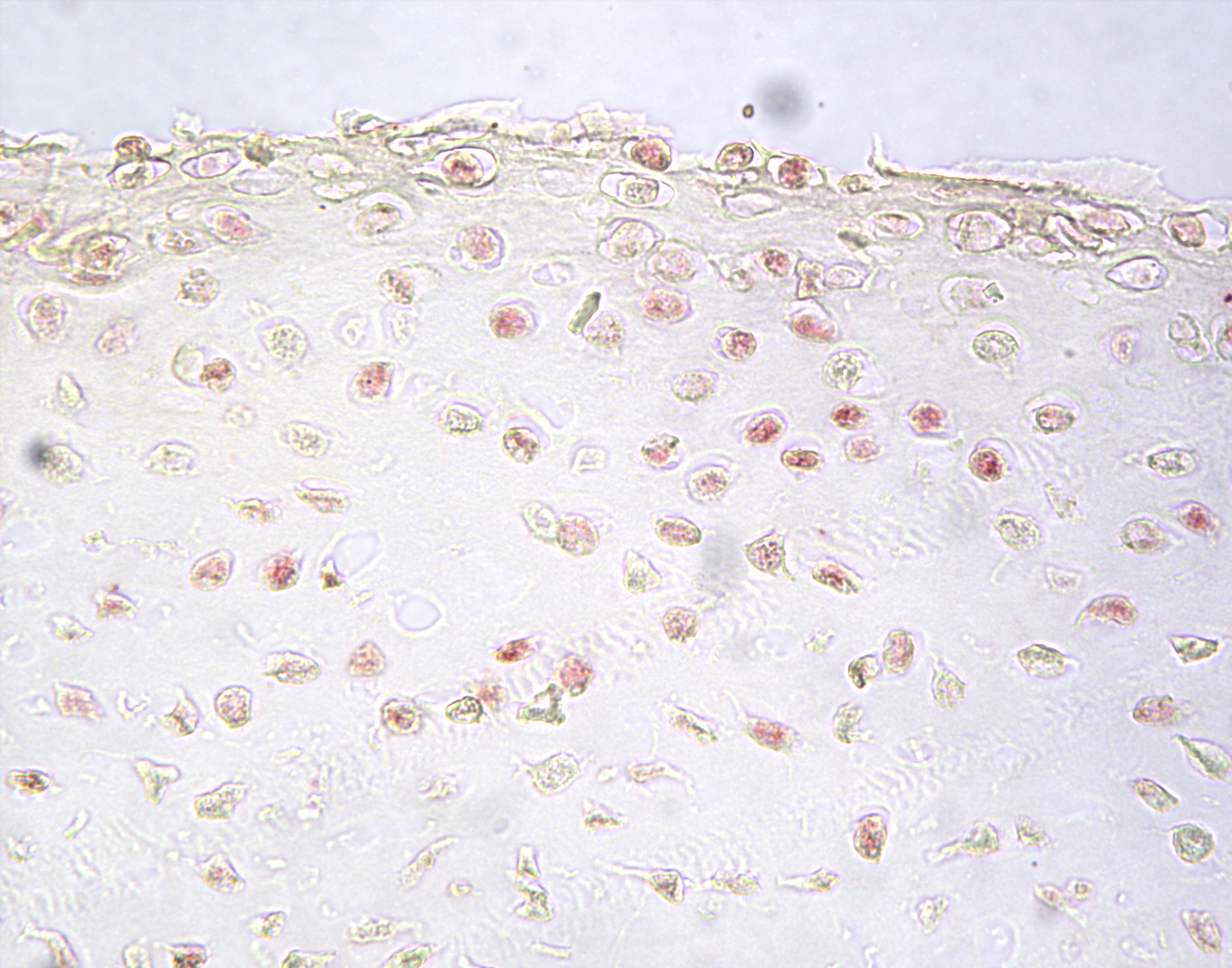

Supplement: Supplementary file 4 — Source Data for Figure 1 [file EMMM-15-e16218-s008.zip › F1/021015d.tif]

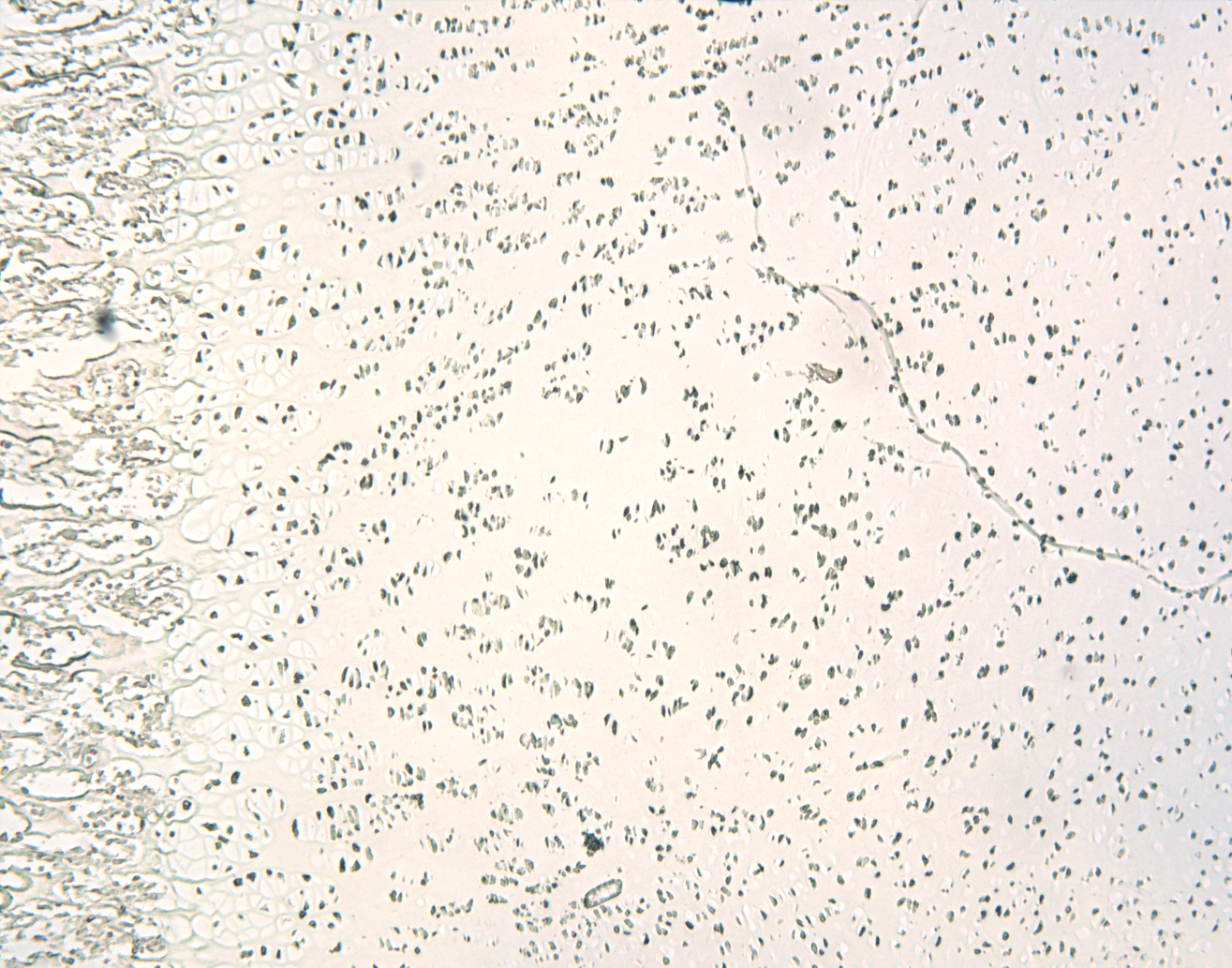

Supplement: Supplementary file 4 — Source Data for Figure 1 [file EMMM-15-e16218-s008.zip › F1/021015j growth plate.tif]

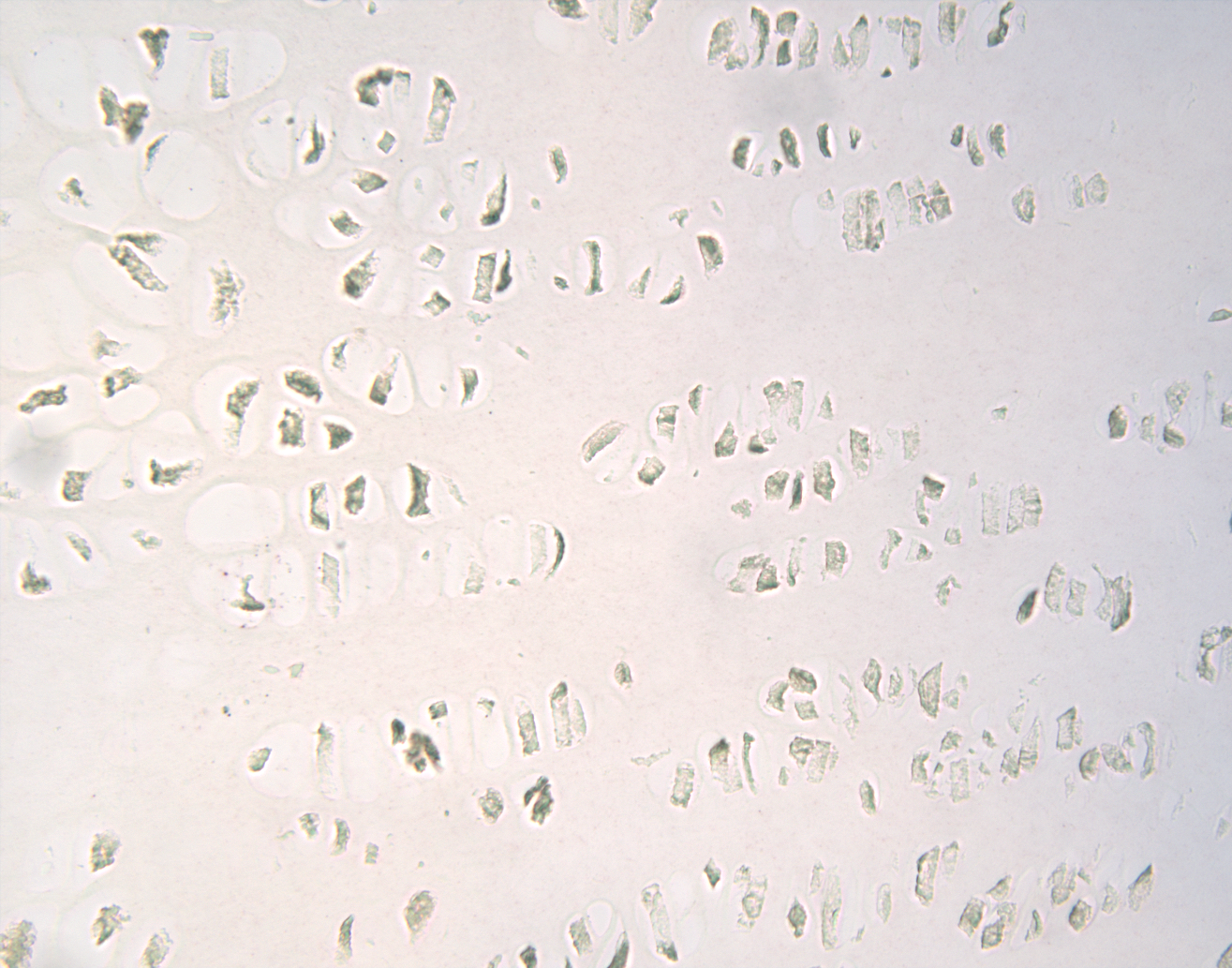

Supplement: Supplementary file 4 — Source Data for Figure 1 [file EMMM-15-e16218-s008.zip › F1/021015l growth plate.tif]

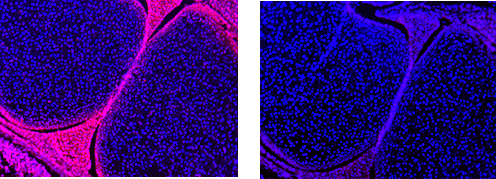

Supplement: Supplementary file 4 — Source Data for Figure 1 [file EMMM-15-e16218-s008.zip › F1/1a.png]

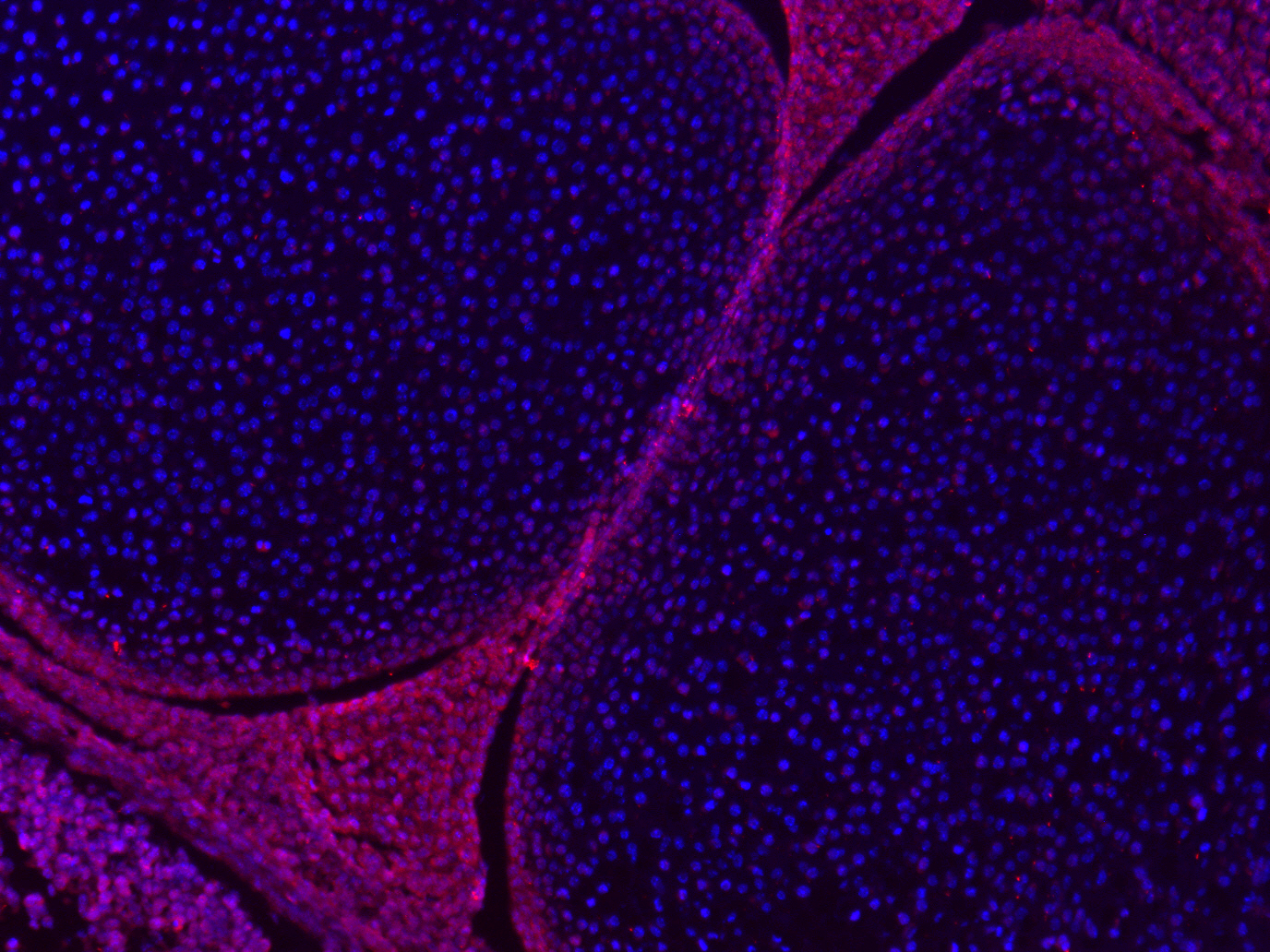

Supplement: Supplementary file 4 — Source Data for Figure 1 [file EMMM-15-e16218-s008.zip › F1/1a-1 x10.tif]

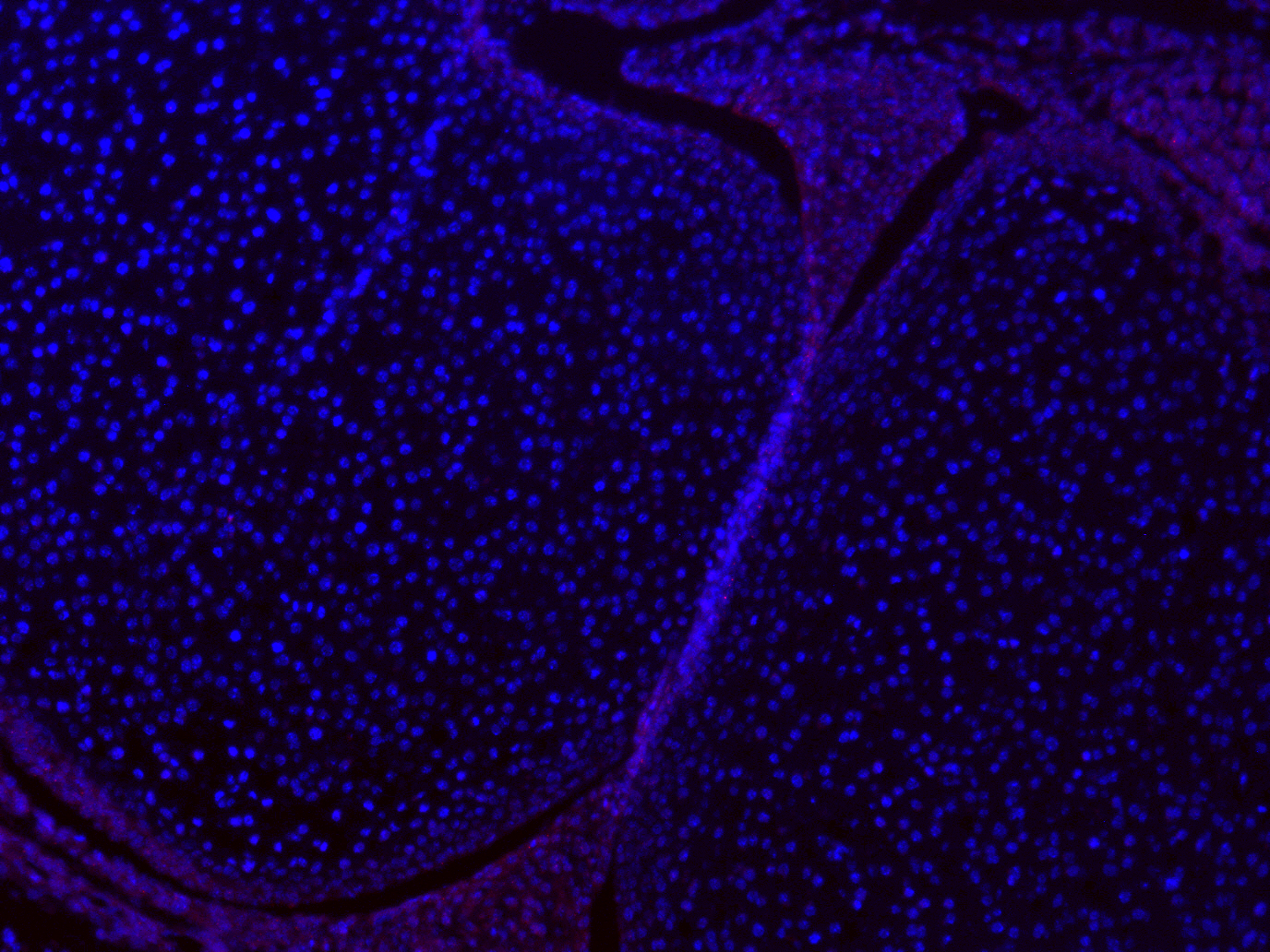

Supplement: Supplementary file 4 — Source Data for Figure 1 [file EMMM-15-e16218-s008.zip › F1/1a-2 IgG x10.tif]

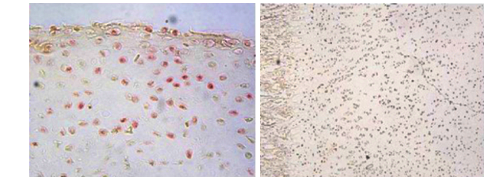

Supplement: Supplementary file 4 — Source Data for Figure 1 [file EMMM-15-e16218-s008.zip › F1/1b.png]

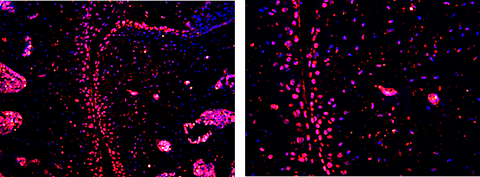

Supplement: Supplementary file 4 — Source Data for Figure 1 [file EMMM-15-e16218-s008.zip › F1/1c.png]

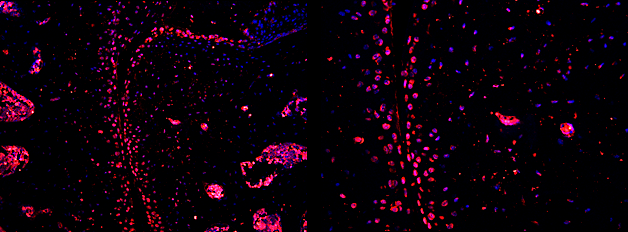

Supplement: Supplementary file 4 — Source Data for Figure 1 [file EMMM-15-e16218-s008.zip › F1/1c.tif]

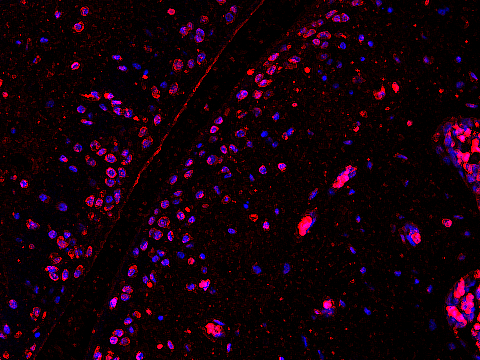

Supplement: Supplementary file 4 — Source Data for Figure 1 [file EMMM-15-e16218-s008.zip › F1/1C/K16/Composite flat.tif (RGB).tif]

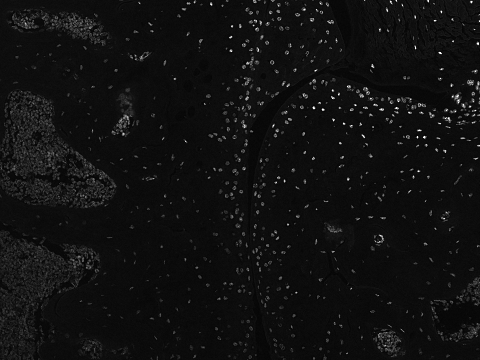

Supplement: Supplementary file 4 — Source Data for Figure 1 [file EMMM-15-e16218-s008.zip › F1/1C/K16/Image004_ch00.tif]

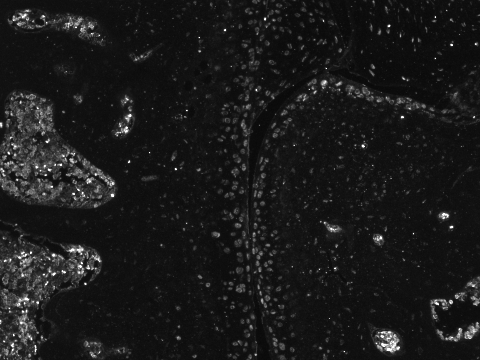

Supplement: Supplementary file 4 — Source Data for Figure 1 [file EMMM-15-e16218-s008.zip › F1/1C/K16/Image005_ch00.tif]

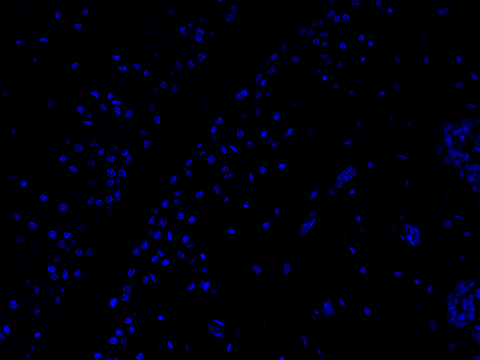

Supplement: Supplementary file 4 — Source Data for Figure 1 [file EMMM-15-e16218-s008.zip › F1/1C/K16/Image013_ch00 edited.tif]

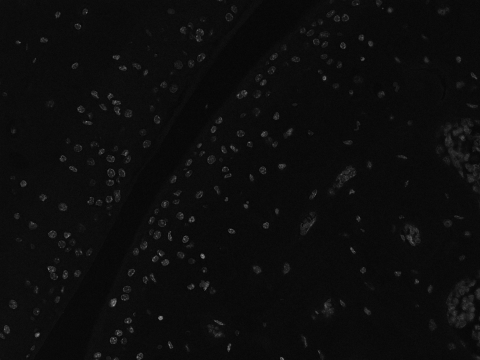

Supplement: Supplementary file 4 — Source Data for Figure 1 [file EMMM-15-e16218-s008.zip › F1/1C/K16/Image013_ch00.tif]

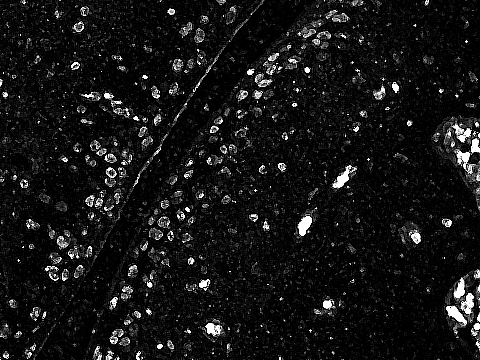

Supplement: Supplementary file 4 — Source Data for Figure 1 [file EMMM-15-e16218-s008.zip › F1/1C/K16/Image015_ch00 edited.tif (red).tif]

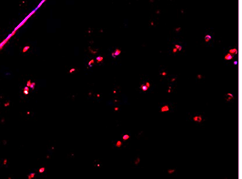

Supplement: Supplementary file 4 — Source Data for Figure 1 [file EMMM-15-e16218-s008.zip › F1/1d.png]

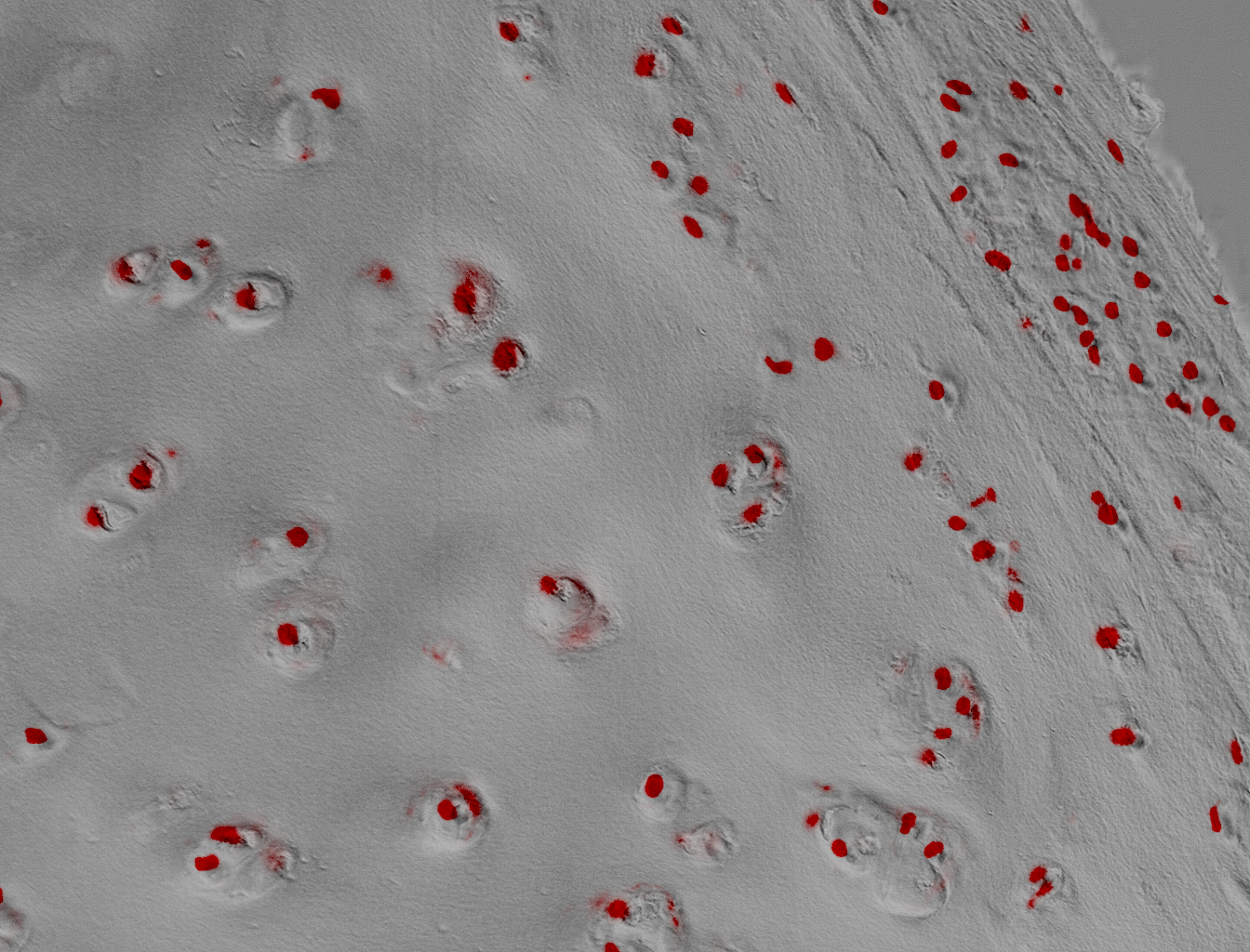

Supplement: Supplementary file 4 — Source Data for Figure 1 [file EMMM-15-e16218-s008.zip › F1/1D/human cartilage 1/GCP2-DAPI DIC.tif]

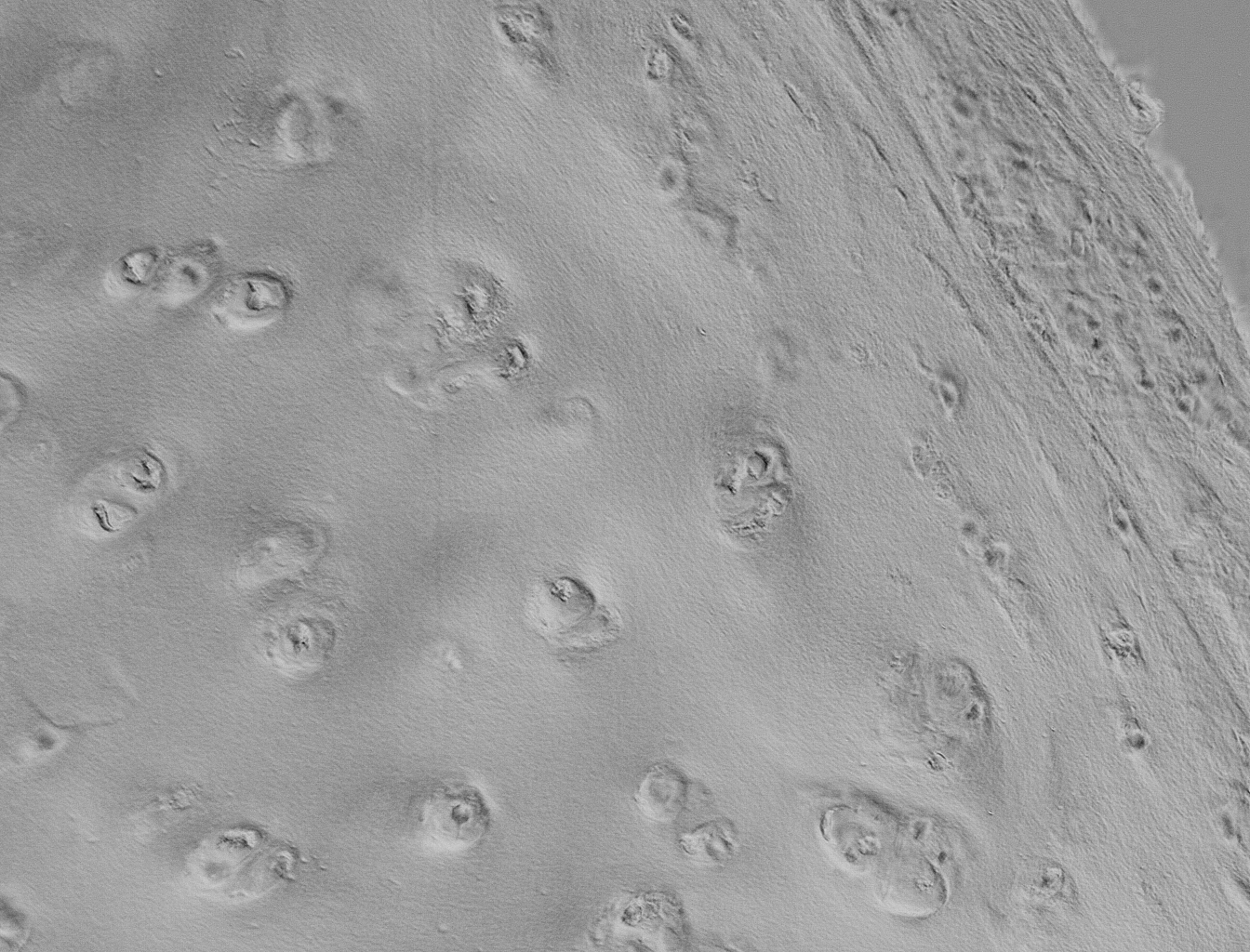

Supplement: Supplementary file 4 — Source Data for Figure 1 [file EMMM-15-e16218-s008.zip › F1/1D/human cartilage 1/Image_2124.tif]

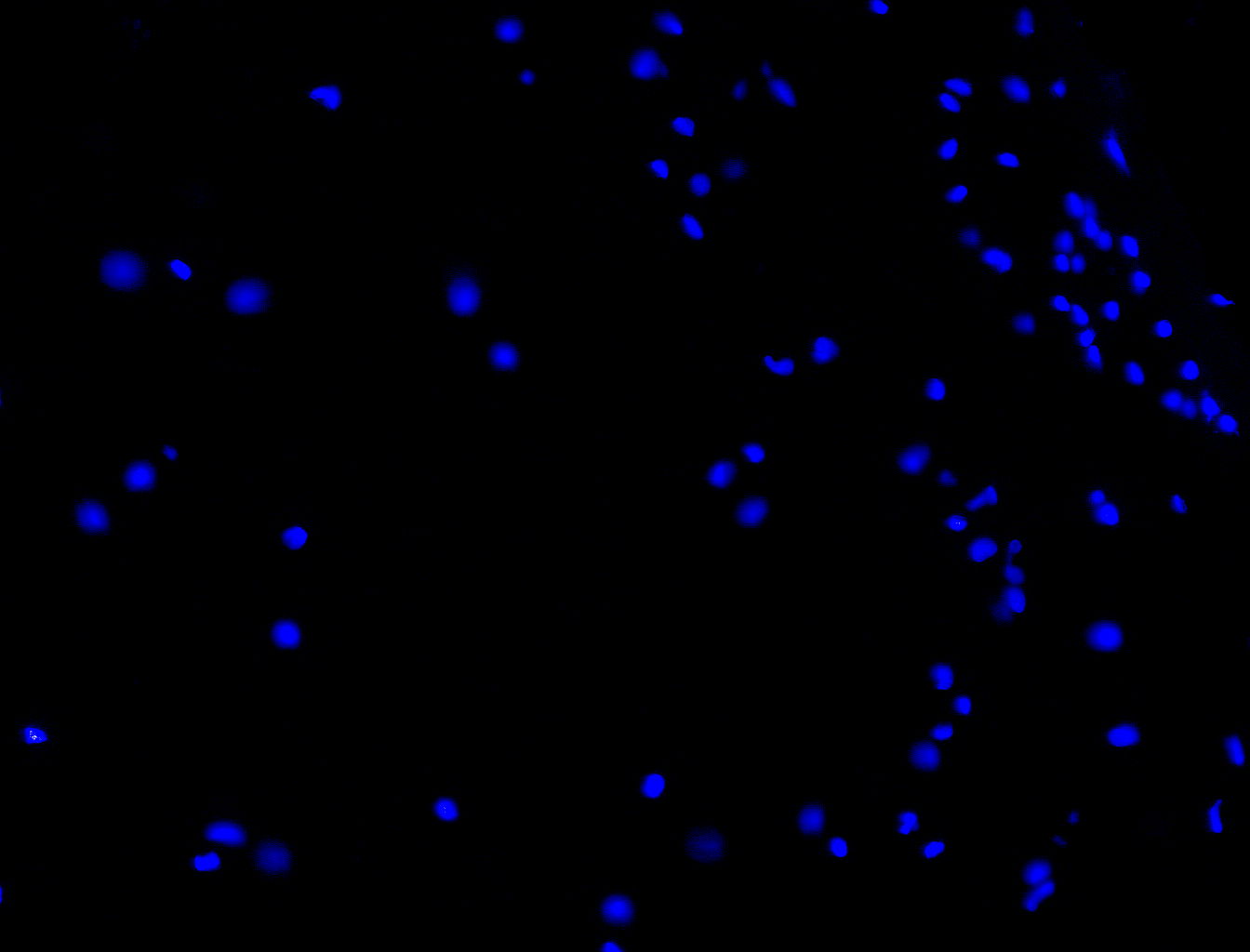

Supplement: Supplementary file 4 — Source Data for Figure 1 [file EMMM-15-e16218-s008.zip › F1/1D/human cartilage 1/Image_2125.tif]

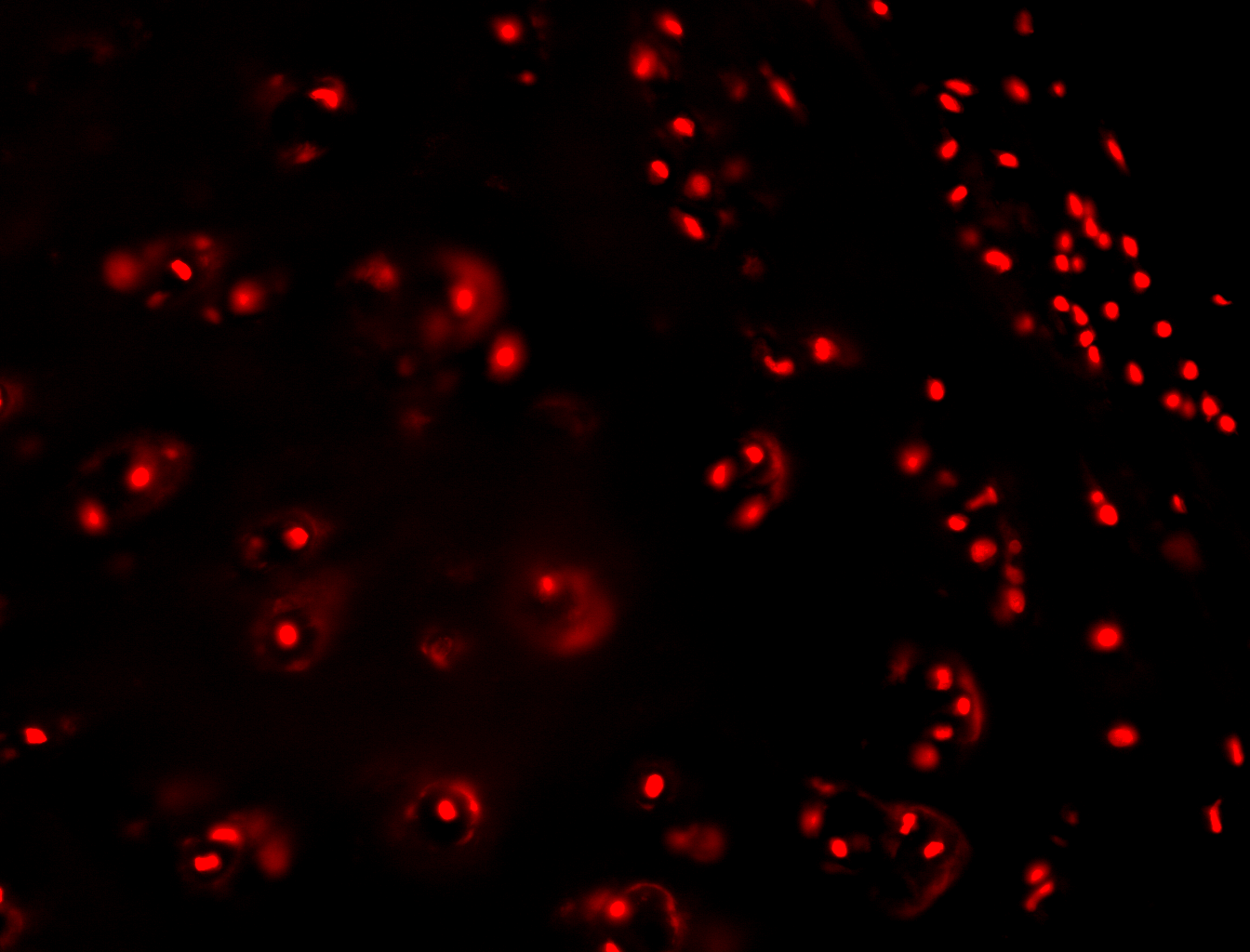

Supplement: Supplementary file 4 — Source Data for Figure 1 [file EMMM-15-e16218-s008.zip › F1/1D/human cartilage 1/Image_2126.tif]

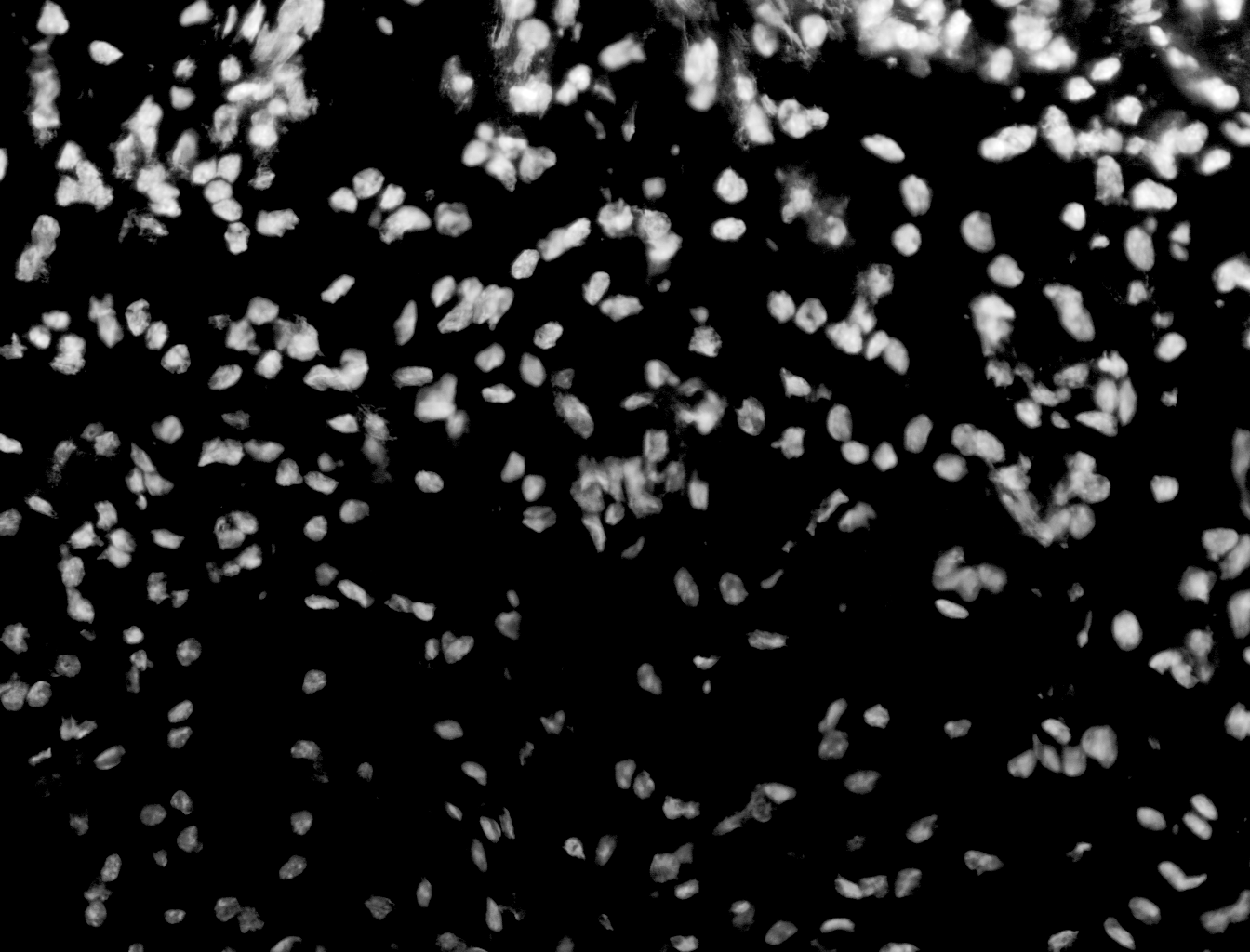

Supplement: Supplementary file 5 — Source Data for Figure 2 [file EMMM-15-e16218-s007.zip › F2/2g GCP-2 blue.tif]

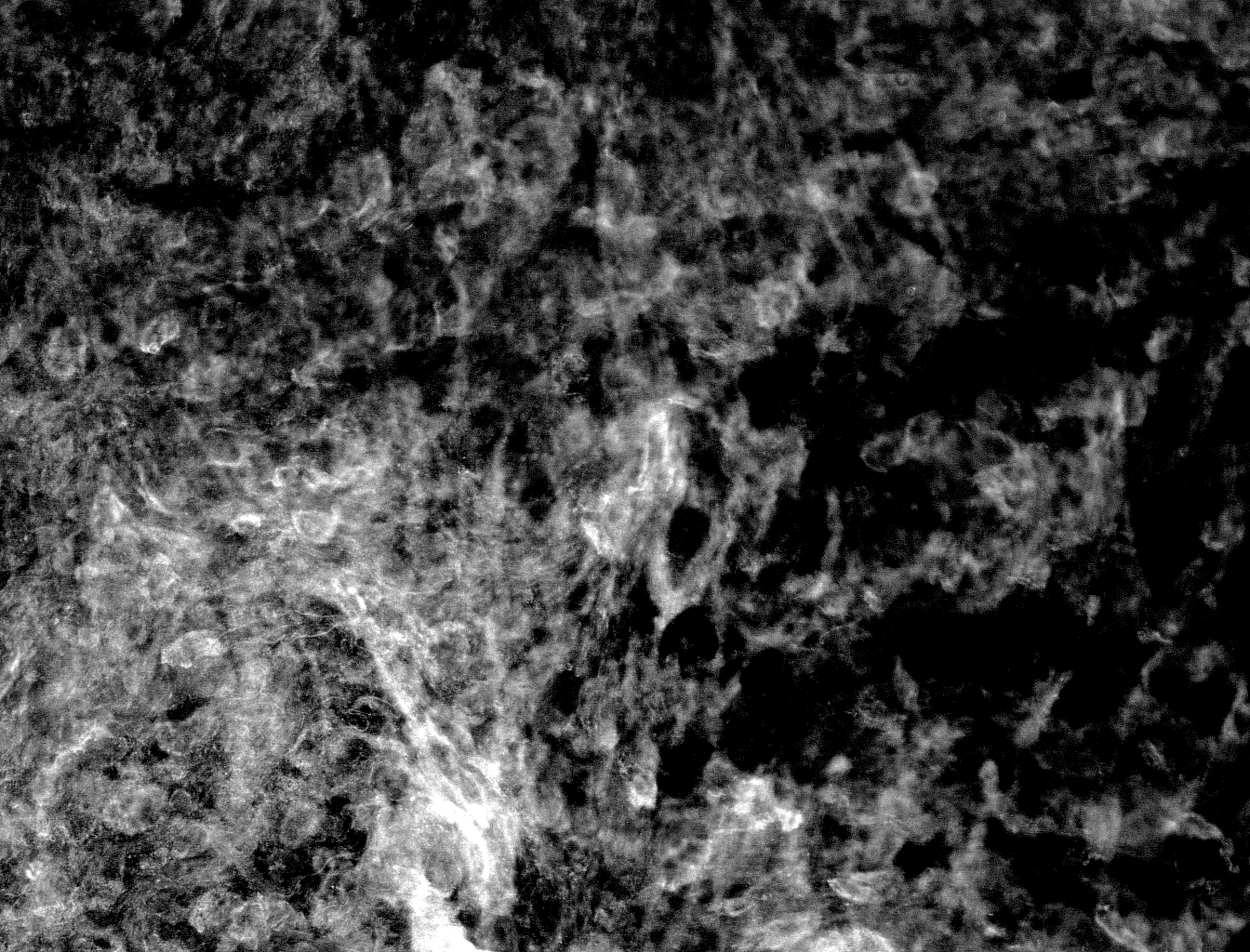

Supplement: Supplementary file 5 — Source Data for Figure 2 [file EMMM-15-e16218-s007.zip › F2/2g GCP-2 composite.tif]

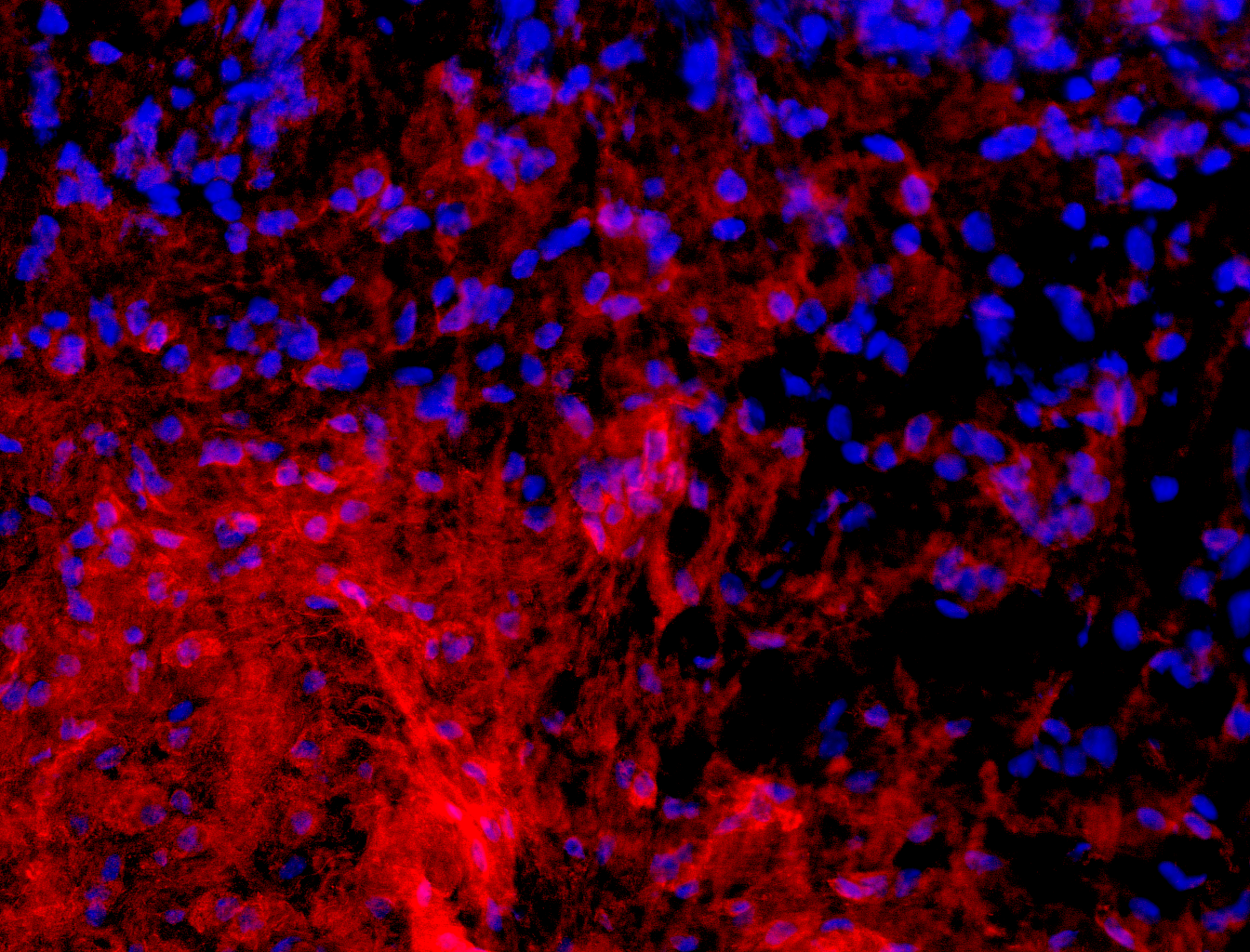

Supplement: Supplementary file 5 — Source Data for Figure 2 [file EMMM-15-e16218-s007.zip › F2/2g GCP-2 composite.tif (RGB) flat.tif (RGB).tif]

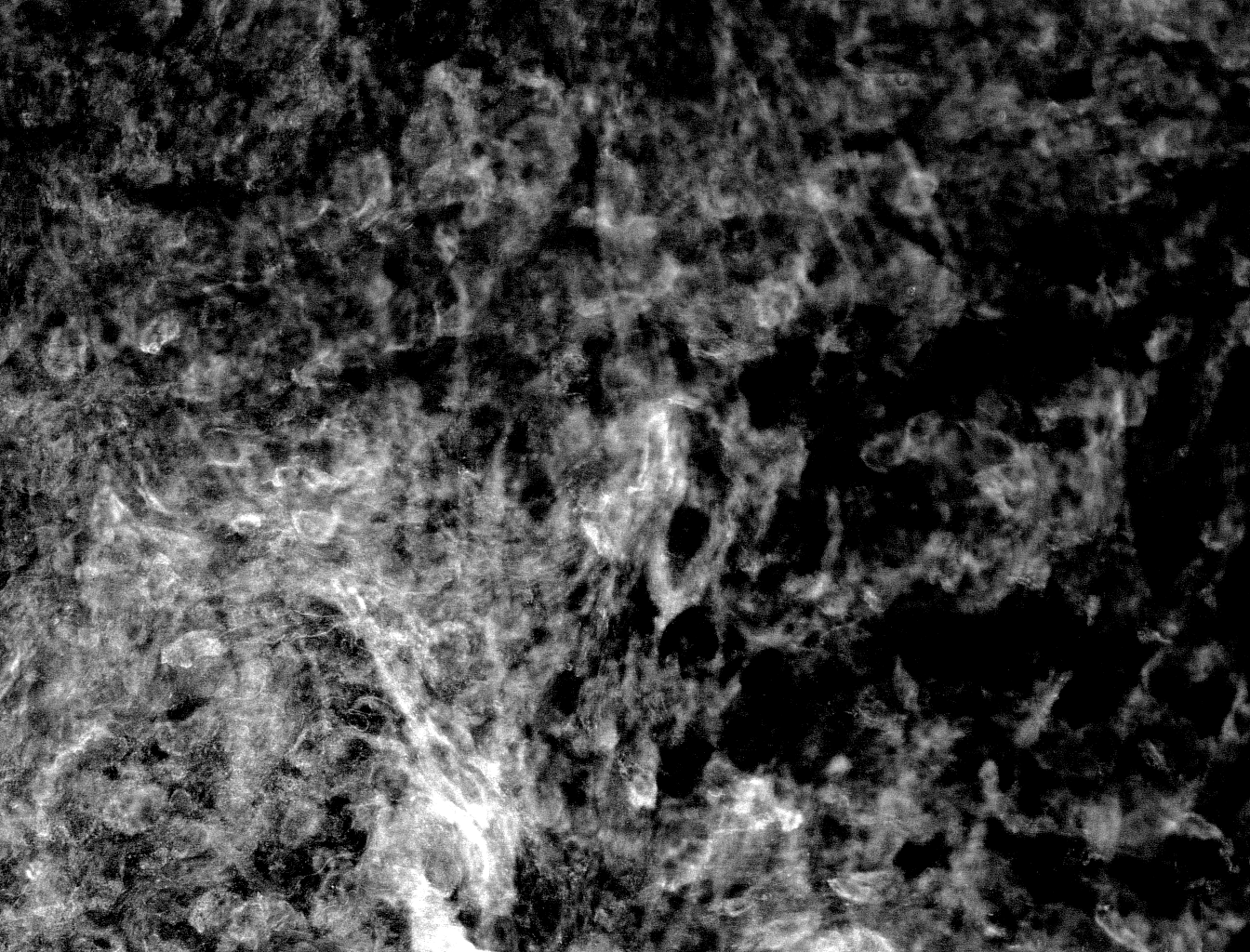

Supplement: Supplementary file 5 — Source Data for Figure 2 [file EMMM-15-e16218-s007.zip › F2/2g GCP-2 red.tif]

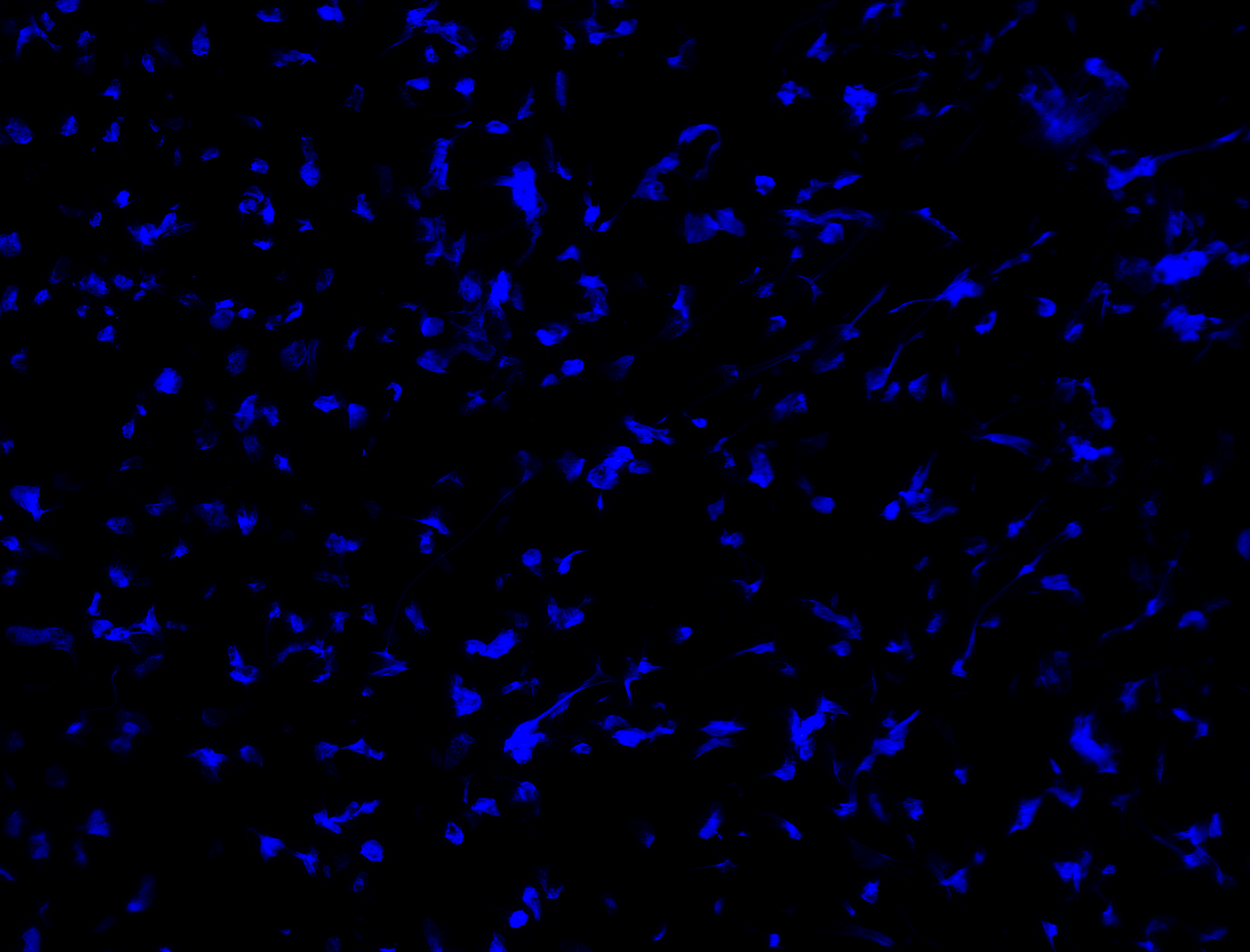

Supplement: Supplementary file 5 — Source Data for Figure 2 [file EMMM-15-e16218-s007.zip › F2/2g GFP blue.tif]

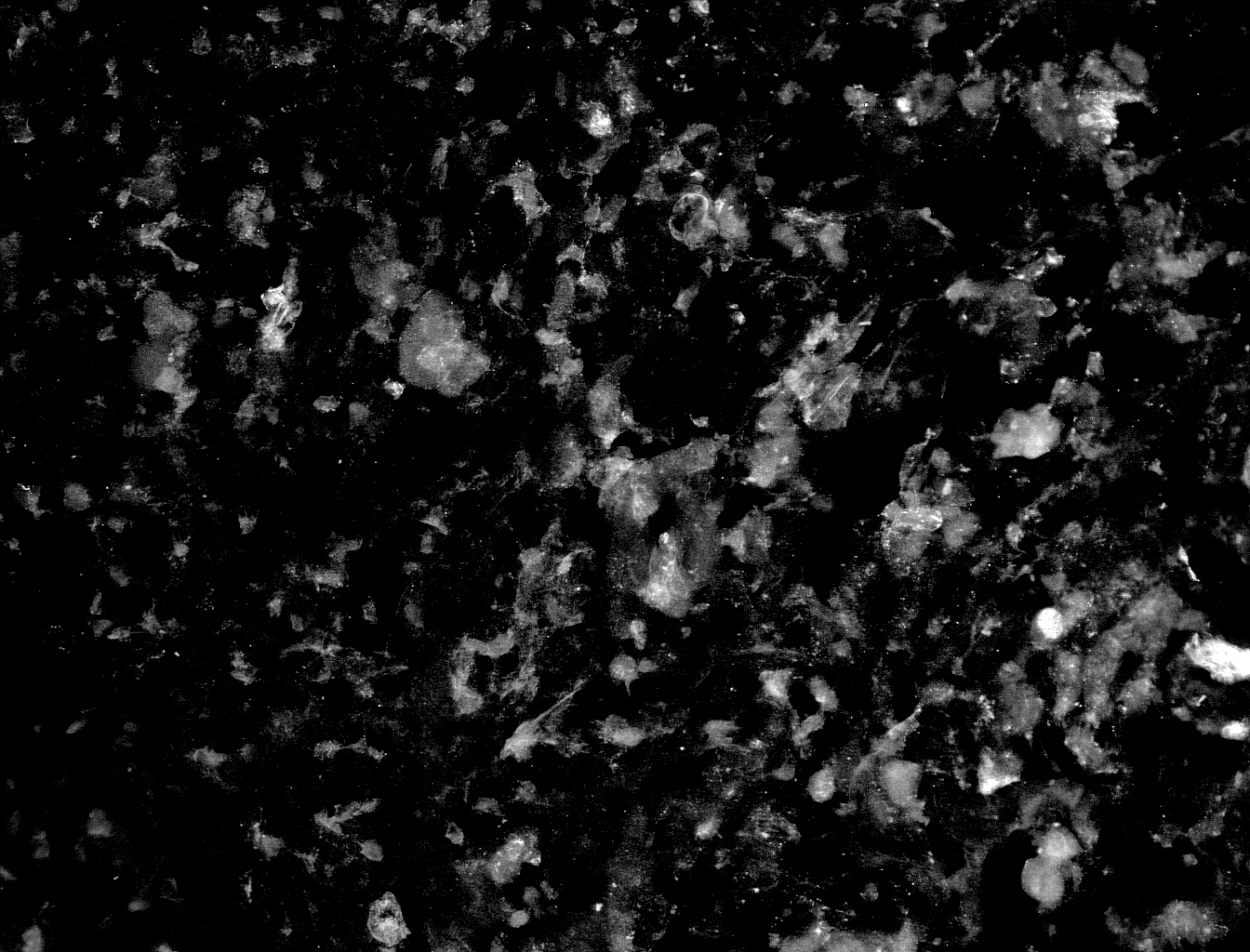

Supplement: Supplementary file 5 — Source Data for Figure 2 [file EMMM-15-e16218-s007.zip › F2/2g gfp composite.tif]

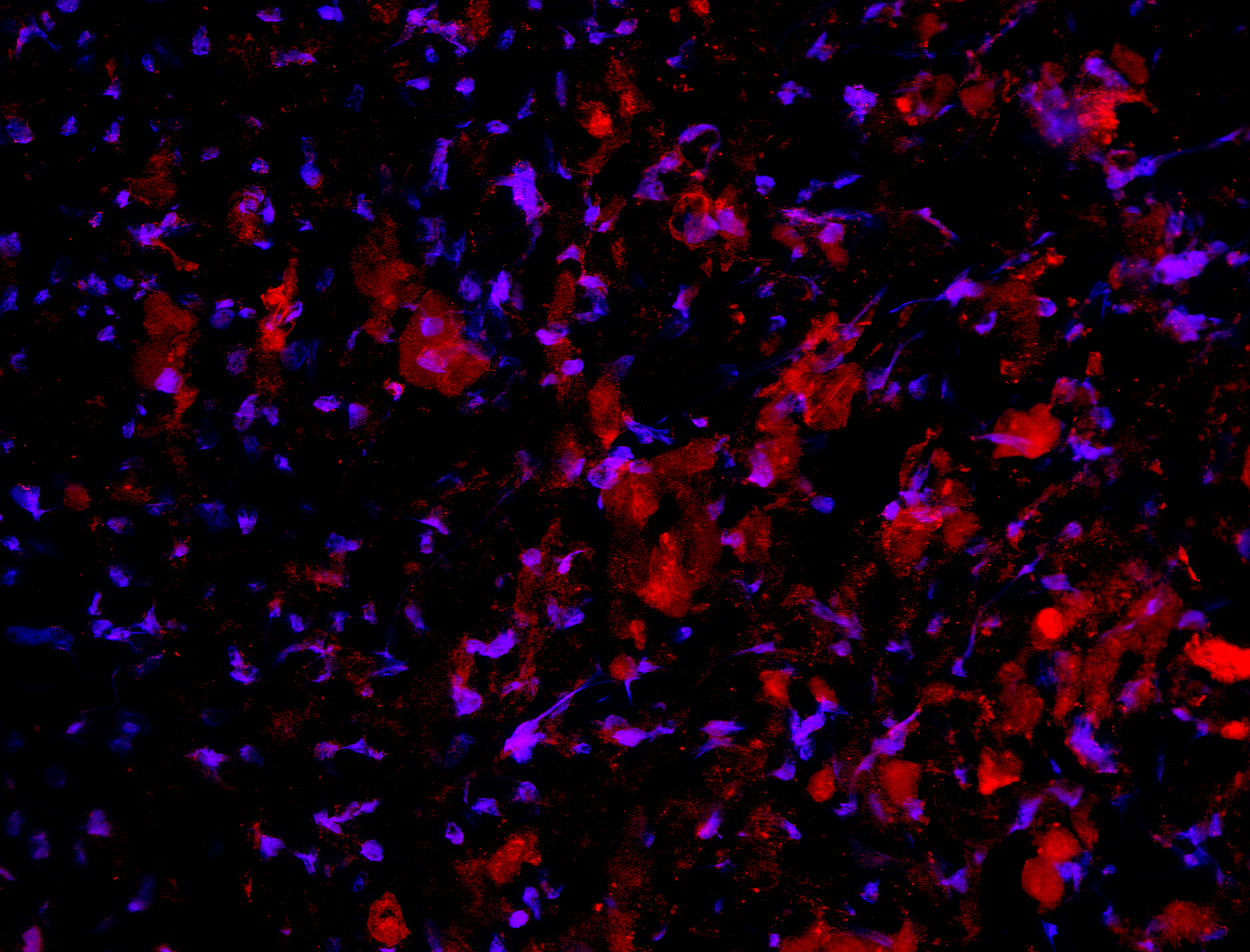

Supplement: Supplementary file 5 — Source Data for Figure 2 [file EMMM-15-e16218-s007.zip › F2/2g gfp flat.tif (RGB).tif]

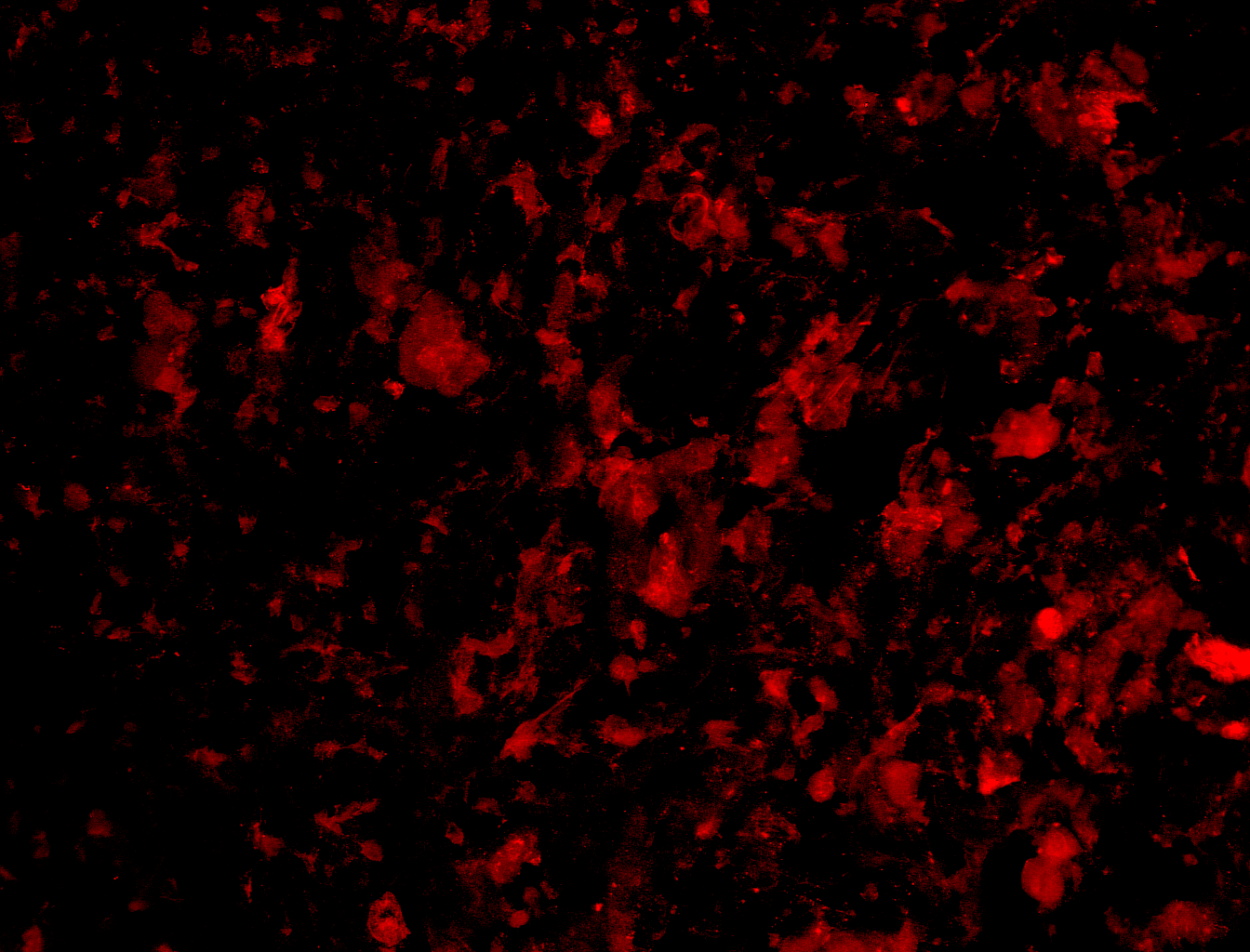

Supplement: Supplementary file 5 — Source Data for Figure 2 [file EMMM-15-e16218-s007.zip › F2/2g GFP red.tif]

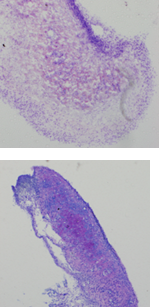

Supplement: Supplementary file 5 — Source Data for Figure 2 [file EMMM-15-e16218-s007.zip › F2/2g.png]

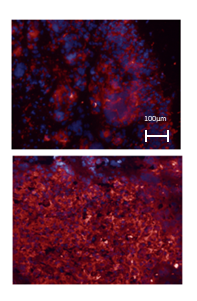

Supplement: Supplementary file 5 — Source Data for Figure 2 [file EMMM-15-e16218-s007.zip › F2/2h.png]

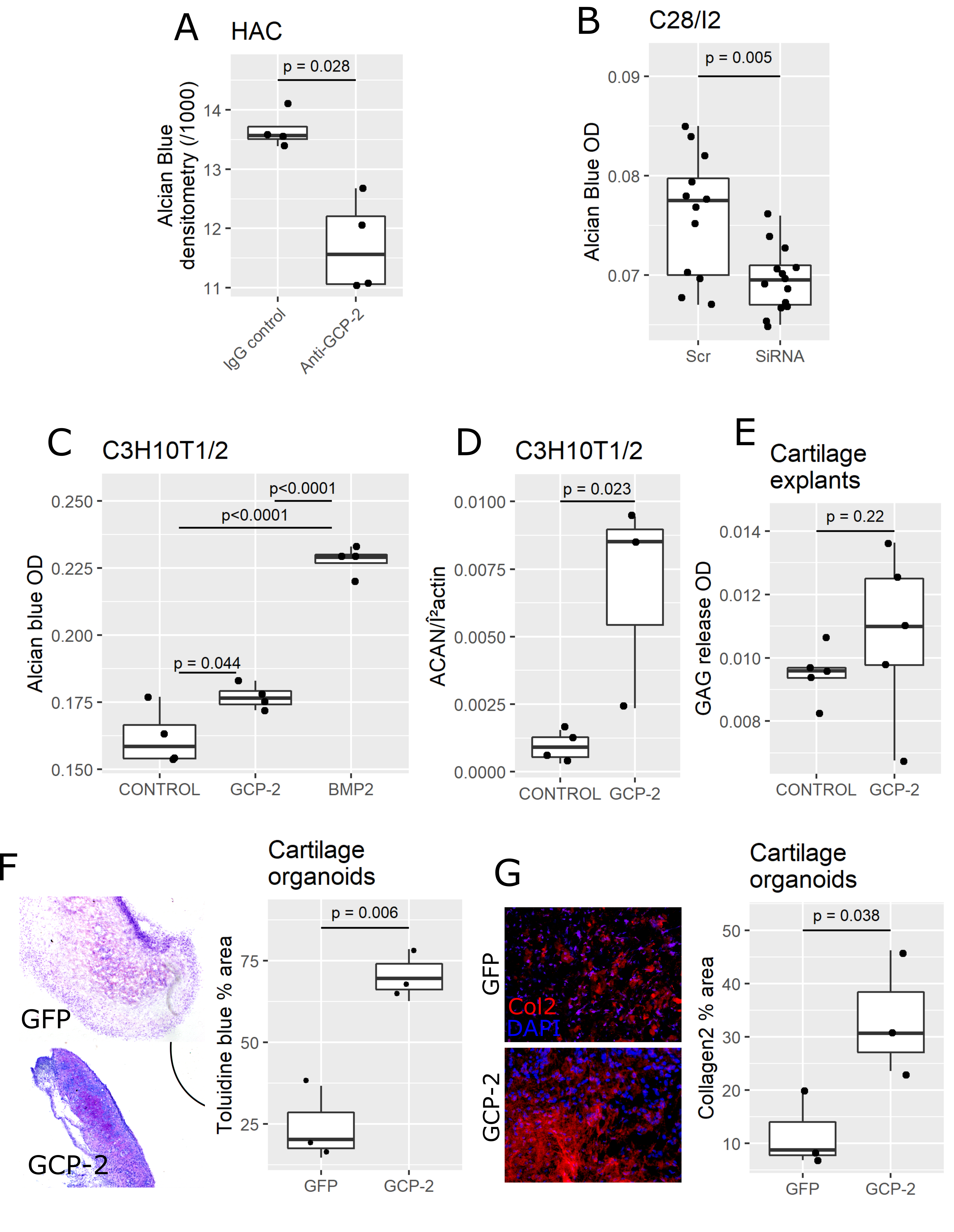

Supplement: Supplementary file 5 — Source Data for Figure 2 [file EMMM-15-e16218-s007.zip › F2/F2.png]

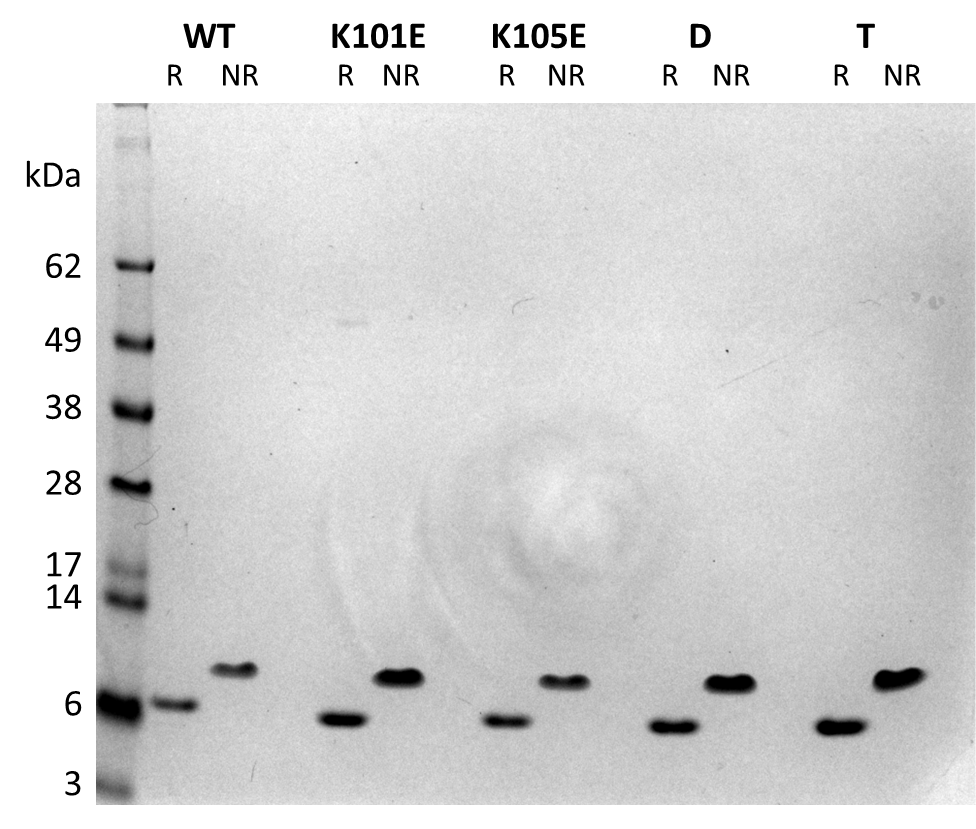

Supplement: Supplementary file 5 — Source Data for Figure 2 [file EMMM-15-e16218-s007.zip › F2/S2F.tif]

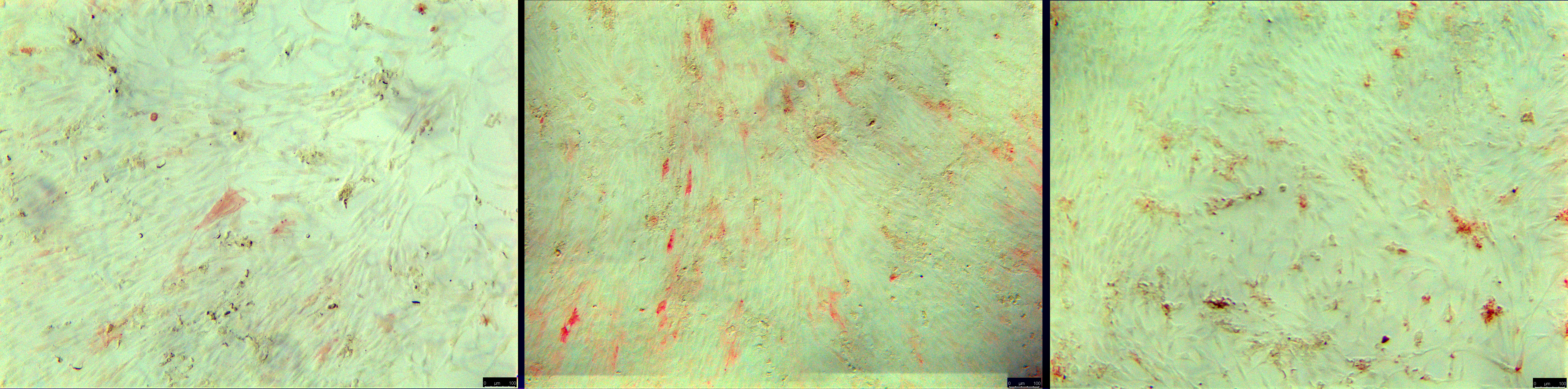

Supplement: Supplementary file 6 — Source Data for Figure 3 [file EMMM-15-e16218-s004.zip › F3/3c edited.png]

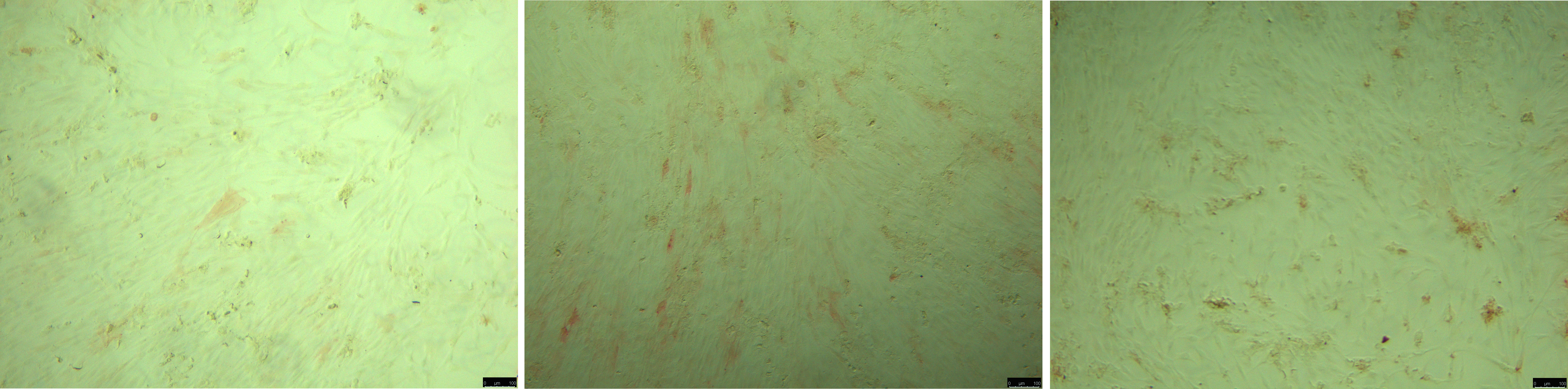

Supplement: Supplementary file 6 — Source Data for Figure 3 [file EMMM-15-e16218-s004.zip › F3/3c original.png]

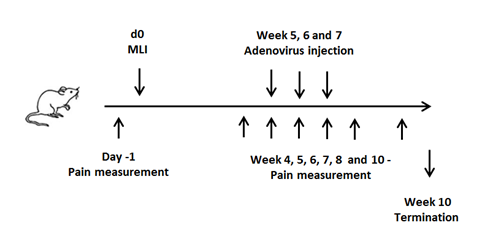

Supplement: Supplementary file 7 — Source Data for Figure 4 [file EMMM-15-e16218-s005.zip › F4/1a.png]

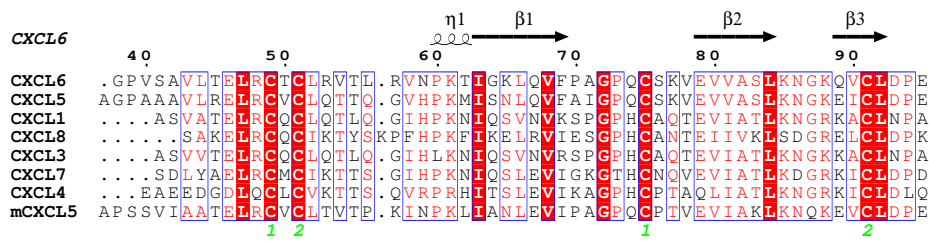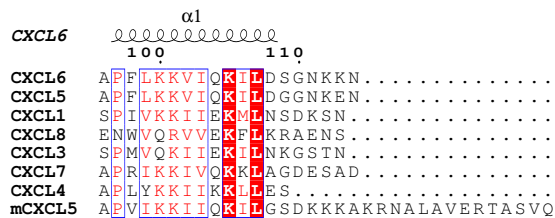

Supplement: Supplementary file 7 — Source Data for Figure 4 [file EMMM-15-e16218-s005.zip › F4/4A.pdf]

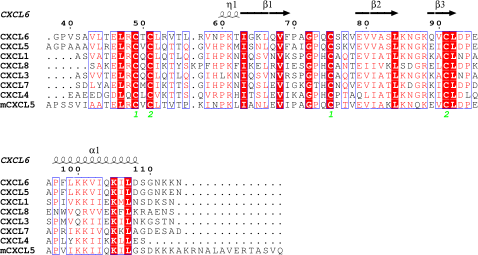

Supplement: Supplementary file 7 — Source Data for Figure 4 [file EMMM-15-e16218-s005.zip › F4/4a.png]

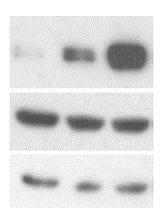

Supplement: Supplementary file 7 — Source Data for Figure 4 [file EMMM-15-e16218-s005.zip › F4/4f.png]

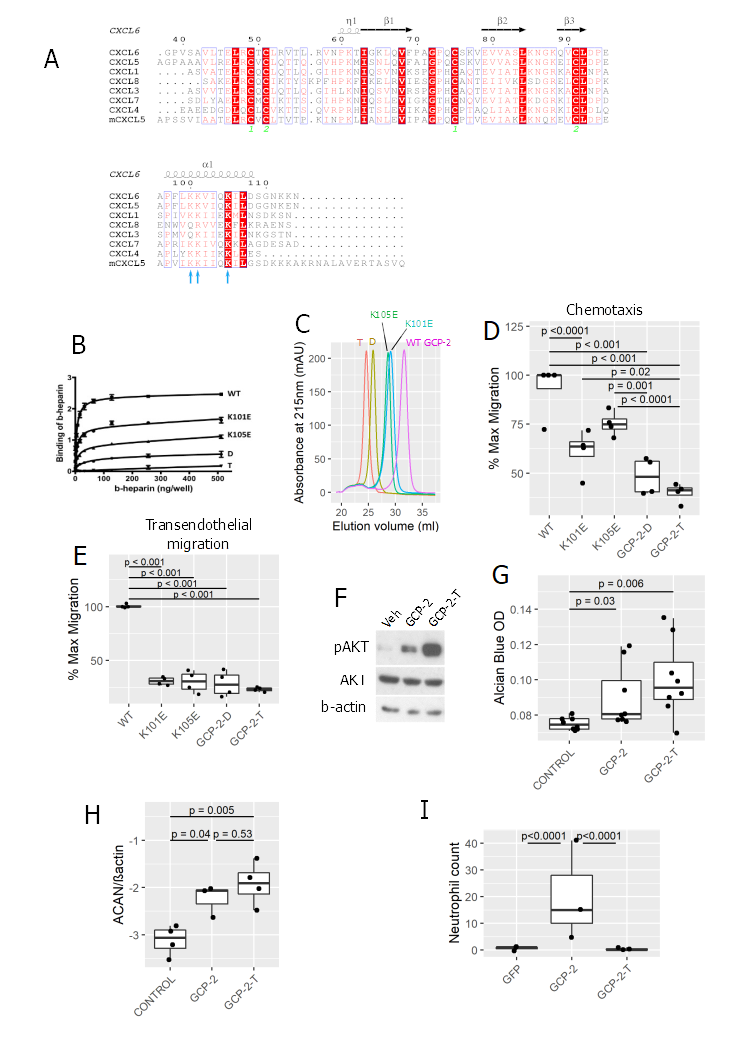

Supplement: Supplementary file 7 — Source Data for Figure 4 [file EMMM-15-e16218-s005.zip › F4/F4.tif]

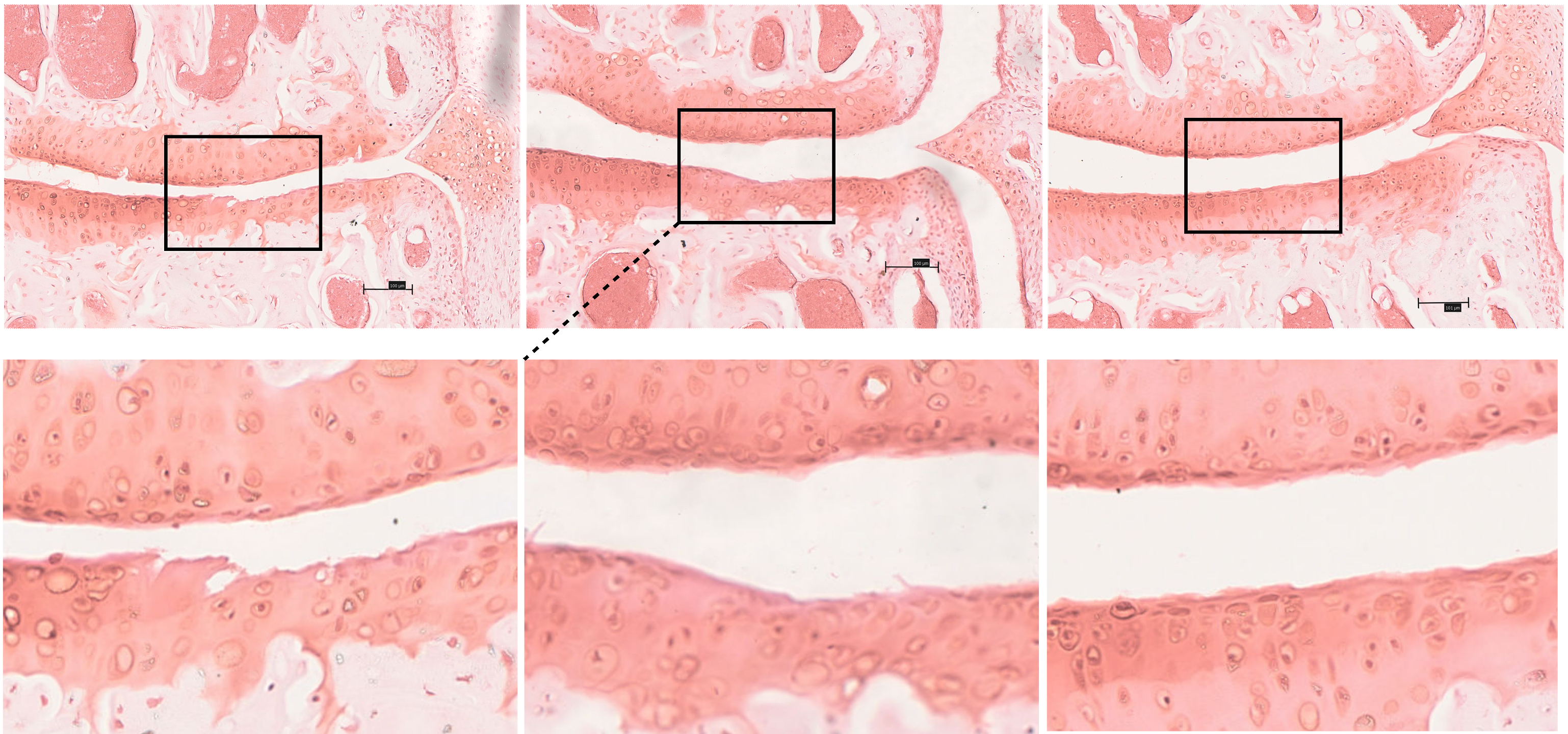

Supplement: Supplementary file 8 — Source Data for Figure 5 [file EMMM-15-e16218-s009.zip › F5/5f.png]

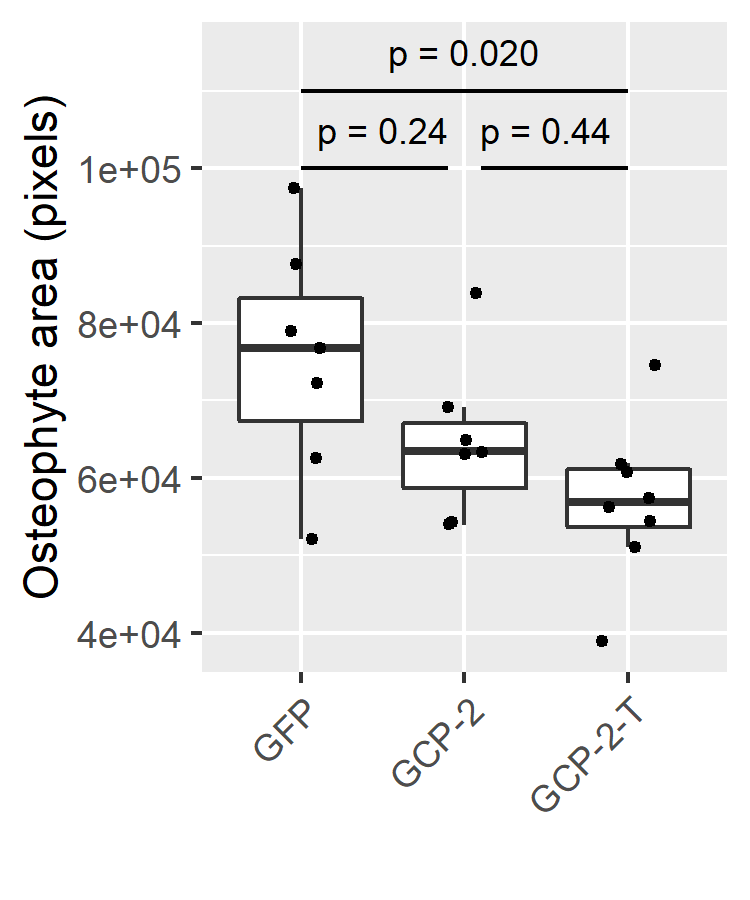

Supplement: Supplementary file 8 — Source Data for Figure 5 [file EMMM-15-e16218-s009.zip › F5/Scripts Fig5/5g.png]

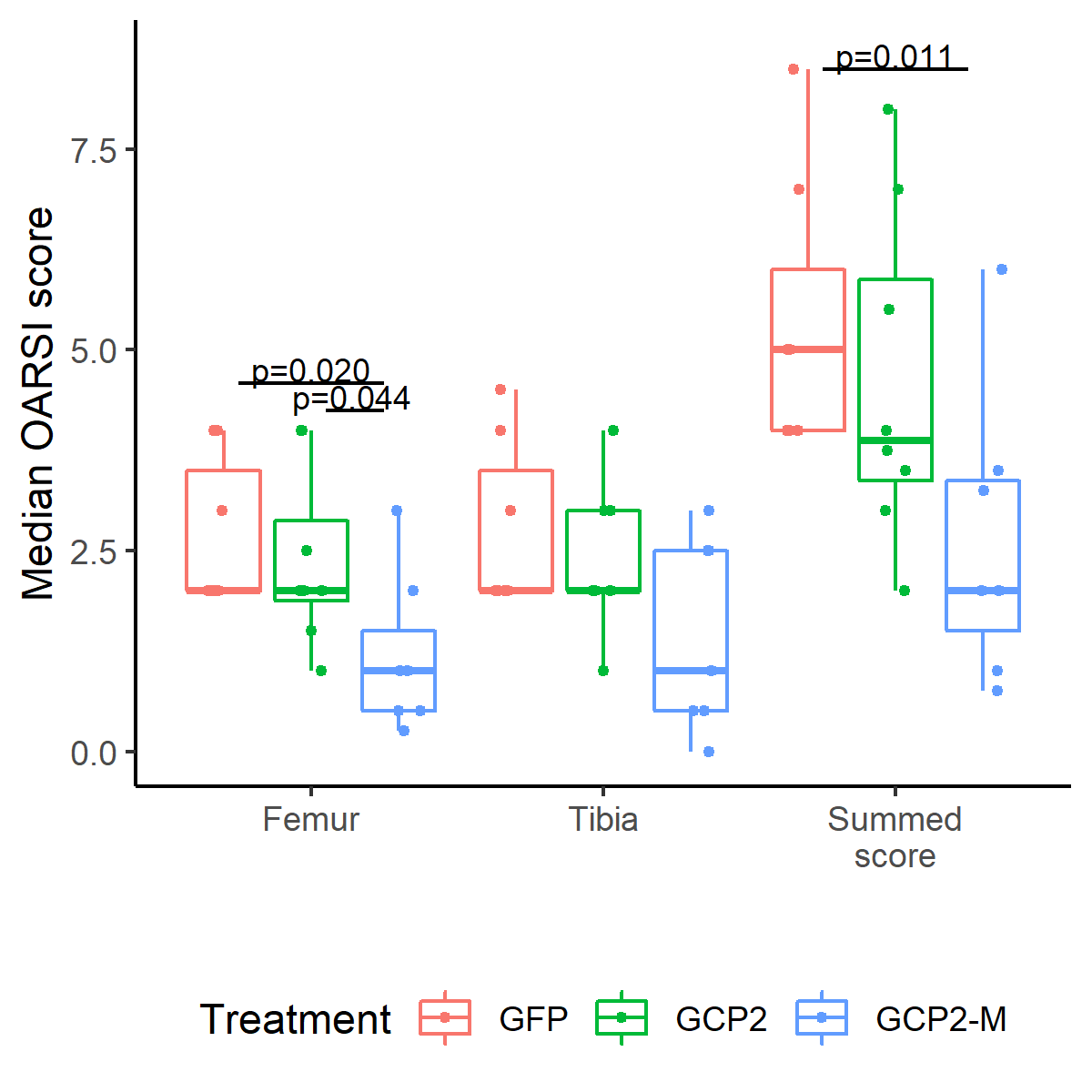

Supplement: Supplementary file 8 — Source Data for Figure 5 [file EMMM-15-e16218-s009.zip › F5/Scripts Fig5/oarsi_medians.png]
