# Supplementary material for: Disease modification and symptom relief in osteoarthritis using a mutated GCP‐2/CXCL6 chemokine
Source: EMBO Mol Med. 2022 Dec 12;15(1):e16218. doi: 10.15252/emmm.202216218 (PMC9832835; doi:10.15252/emmm.202216218)
Supplement: Supplementary file 9 [file EMMM-15-e16218-s001.pdf]

EMBO Molecular Medicine

Disease modification and symptom relief in osteoarthritis using a mutated GCP-2/CXCL6 chemokine

Sara Caxaria, Nikolaos Kouvatsos, Suzanne Eldridge, Mario Alvarez-Fellas, Anne-Sophie Thorup, Daniela Cici, Aida Barawi, Ammaarah Arshed, Danielle Strachan, Giulia Carletti, Xinying Huang, Sabah Bharde, Melody Deniz, Jacob Wilson, Bethan Thomas, Costantino Pitzalis, Francesco Cantatore, Manasi Sayilekshmy, Shafaq Sikandar, Frank Luyten, Thomas Pap, Joanna Sherwood, Anthony Day, and Francesco Dell'Accio
DOI: 10.15252/emmm.202216218

Corresponding authors: Francesco Dell'Accio (fdellaccio@gmail.com) , Anthony Day (anthony.day@manchester.ac.uk)

Review Timeline:

Submission Date:	27th Apr 22
Editorial Decision:	17th May 22
Revision Received:	23rd Aug 22
Editorial Decision:	23rd Sep 22
Revision Received:	12th Oct 22
Editorial Decision:	26th Oct 22
Revision Received:	9th Nov 22
Accepted:	11th Nov 22

Editor: Lise Roth

Transaction Report:

(Note: With the exception of the correction of typographical or spelling errors that could be a source of ambiguity, letters and reports are not edited. Depending on transfer agreements, referee reports obtained elsewhere may or may not be included in this compilation. Referee reports are anonymous unless the Referee chooses to sign their reports.)

17th May 2022

Dear Dr. Dell'Accio,

Thank you for the submission of your manuscript to EMBO Molecular Medicine. We have now received feedback from the three reviewers who agreed to evaluate your manuscript. As you will see from the reports below, the referees acknowledge the translational interest of the study, but also raise a number of concerns that should be adequately addressed in a revised version of this manuscript.

In particular, a better characterization of the mouse model of osteoarthritis in absence or presence of GCP-2-T should be provided (including evaluation of cartilage anabolism, catabolism, chondrocyte apoptosis, AKT signaling, inflammation).
On the other hand, the referees agreed that systemic administration of GCP-2-T would not be required, provided an adequate discussion on the clinical relevance of GCP-2-T.

Addressing the other reviewers' concerns in full will be necessary for further considering the manuscript in our journal, and acceptance of the manuscript will entail a second round of review. EMBO Molecular Medicine encourages a single round of revision only and therefore, acceptance or rejection of the manuscript will depend on the completeness of your responses included in the next, final version of the manuscript. For this reason, and to save you from any frustrations in the end, I would strongly advise against returning an incomplete revision.

Revised manuscripts should be submitted within three months of a request for revision; they will otherwise be treated as new submissions, except under exceptional circumstances in which a short extension is obtained from the editor.

When submitting your revised manuscript, please carefully review the instructions that follow below. We perform an initial quality control of all revised manuscripts before re-review; failure to include requested items will delay the evaluation of your revision.

We require:

1) A .docx formatted version of the manuscript text (including legends for main figures, EV figures and tables). Please make sure that the changes are highlighted to be clearly visible.

2) Individual production quality figure files as .eps, .tif, .jpg (one file per figure). For guidance, download the 'Figure Guide PDF' (https://www.embopress.org/page/journal/17574684/authorguide#figureformat).

3) A .docx formatted letter INCLUDING the reviewers' reports and your detailed point-by-point responses to their comments. As part of the EMBO Press transparent editorial process, the point-by-point response is part of the Review Process File (RPF), which will be published alongside your paper.

4) A complete author checklist, which you can download from our author guidelines (https://www.embopress.org/page/journal/17574684/authorguide#submissionofrevisions). Please insert information in the checklist that is also reflected in the manuscript. The completed author checklist will also be part of the RPF.

5) It is mandatory to include a 'Data Availability' section after the Materials and Methods. Before submitting your revision, primary datasets produced in this study need to be deposited in an appropriate public database, and the accession numbers and database listed under 'Data Availability'. Please remember to provide a reviewer password if the datasets are not yet public (see https://www.embopress.org/page/journal/17574684/authorguide#dataavailability).
In case you have no data that requires deposition in a public database, please state so in this section. Note that the Data Availability Section is restricted to new primary data that are part of this study.

6) For data quantification: please specify the name of the statistical test used to generate error bars and P values, the number (n) of independent experiments (specify technical or biological replicates) underlying each data point and the test used to calculate p-values in each figure legend. The figure legends should contain a basic description of n, P and the test applied. Graphs must include a description of the bars and the error bars (s.d., s.e.m.). Exact p values must be provided.

7) We would also encourage you to include the source data for figure panels that show essential data. Numerical data should be provided as individual .xls or .csv files (including a tab describing the data). For blots or microscopy, uncropped images should be submitted (using a zip archive if multiple images need to be supplied for one panel). Additional information on source data and instruction on how to label the files are available at
.

8) Our journal encourages inclusion of *data citations in the reference list* to directly cite datasets that were re-used and

obtained from public databases. Data citations in the article text are distinct from normal bibliographical citations and should directly link to the database records from which the data can be accessed. In the main text, data citations are formatted as follows: "Data ref: Smith et al, 2001" or "Data ref: NCBI Sequence Read Archive PRJNA342805, 2017". In the Reference list, data citations must be labeled with "[DATASET]". A data reference must provide the database name, accession number/identifiers and a resolvable link to the landing page from which the data can be accessed at the end of the reference. Further instructions are available at .

9) We replaced Supplementary Information with Expanded View (EV) Figures and Tables that are collapsible/expandable online. A maximum of 5 EV Figures can be typeset. EV Figures should be cited as 'Figure EV1, Figure EV2" etc... in the text and their respective legends should be included in the main text after the legends of regular figures.
- For the figures that you do NOT wish to display as Expanded View figures, they should be bundled together with their legends in a single PDF file called *Appendix*, which should start with a short Table of Content. Appendix figures should be referred to in the main text as: "Appendix Figure S1, Appendix Figure S2" etc.
- Additional Tables/Datasets should be labeled and referred to as Table EV1, Dataset EV1, etc. Legends have to be provided in a separate tab in case of .xls files. Alternatively, the legend can be supplied as a separate text file (README) and zipped together with the Table/Dataset file.
See detailed instructions here:
.

10) For more information: There is space at the end of each article to list relevant web links for further consultation by our readers. Could you identify some relevant ones and provide such information as well? Some examples are patient associations, relevant databases, OMIM/proteins/genes links, author's websites, etc...

11) Conflict of interest: We updated our journal's competing interests policy in January 2022 and request authors to consider both actual and perceived competing interests. Please review the policy https://www.embopress.org/competing-interests and update your competing interests if necessary.

12) Every published paper now includes a 'Synopsis' to further enhance discoverability. Synopses are displayed on the journal webpage and are freely accessible to all readers. They include a short stand first (maximum of 300 characters, including space) as well as 2-5 one-sentences bullet points that summarizes the paper. Please write the bullet points to summarize the key NEW findings. They should be designed to be complementary to the abstract - i.e. not repeat the same text. We encourage inclusion of key acronyms and quantitative information (maximum of 30 words / bullet point). Please use the passive voice. Please attach these in a separate file or send them by email, we will incorporate them accordingly.
Please also suggest a striking image or visual abstract to illustrate your article as a PNG file 550 px wide x 300-600 px high.

13) As part of the EMBO Publications transparent editorial process initiative (see our Editorial at http://embomolmed.embopress.org/content/2/9/329), EMBO Molecular Medicine will publish online a Review Process File (RPF) to accompany accepted manuscripts.
In the event of acceptance, this file will be published in conjunction with your paper and will include the anonymous referee reports, your point-by-point response and all pertinent correspondence relating to the manuscript. Let us know whether you agree with the publication of the RPF and as here, if you want to remove or not any figures from it prior to publication.
Please note that the Authors checklist will be published at the end of the RPF.

EMBO Molecular Medicine has a "scooping protection" policy, whereby similar findings that are published by others during review or revision are not a criterion for rejection. Should you decide to submit a revised version, I do ask that you get in touch after three months if you have not completed it, to update us on the status.

I look forward to receiving your revised manuscript.

Yours sincerely,

Lise Roth

Lise Roth, PhD
Senior Editor
EMBO Molecular Medicine

***** Reviewer's comments *****

Referee #1 (Comments on Novelty/Model System for Author):

This manuscript is of good quality. The only limitation concerns the use of adenoviral vectors to deliver the cytokine that prevents OA. It would be interesting to test a different way to administer this molecule, a way that could/should be more clinically relevant.

Referee #1 (Remarks for Author):

Caxaria and colleagues have generated a mutated version of GCP-2 (Granulocyte chemotactic protein 2 also known as CXCL6 in humans and CXCL5 in mice), the ligand of CXCR2. This mutated version (GCP-2-T) does not induce inflammation (unlike its wild type counterpart) and promotes cartilage anabolism, while preventing chondrocyte hypertrophy in vitro. Intra-articular administration of adenovirus encoding GCP-2-T ameliorates cartilage destruction and incapacitance in mice after menisco-ligament injury.

This manuscript is well-written and present novel and important data. There a only a few points that should be addressed to further improve the strength of this work.

1- The relative expression of additional anabolic markers (e.g. COL2) and catabolic markers (e.g. MMP13, ADAMTS) should be evaluated in all relevant experiments performed in this study (Fig. 1 to 4). ColX mRNA expression should be evaluated in addition to Runx2 as marker of hypertrophy (Fig. 3A). Additional representative photographic illustrations could be added to the figures wherever the data are represented only as graphs.
2- Figure 5, hypertrophic (COLX), anabolic (ACAN or COL2) and catabolic (MMP13) markers should be evaluated by immunostaining on sections. It would be also interesting to assess neutrophil infiltration, apoptosis and AKT activation (Phospho-AKT) in vivo after GPC-2 and GPC-2-T "treatment". It is important to show and quantify cartilage degradation at week 5, prior adenoviral injections. Does GPC-2-T really revert the defects or does it only limit/stop further destructions taking place between week 5 and week 10 in the control GFP group? This point should be better discussed in the manuscript. Would inoculating GPC-2-T adenovirus since the beginning (Day 1) totally prevent cartilage destruction and increased subchondral bone volume?
3- It would be interesting to evaluate whether systemic administration of GPC-2-T could be used to achieve the same effects that are observed after intra-articular injections of adenovirus. What is the half-life of this molecule? Could it be encapsulated in extracellular vesicles? The possibility of using this molecule in clinic should be better discussed in the manuscript.

Referee #2 (Remarks for Author):

General: This manuscript reports interesting and novel findings addressing not only the mechanism by which GCP-2, a ligand chemokine receptor CXCR2, promotes cartilage homeostasis in the developing embryo and in adult articular cartilage, but also shows how designing a triple-mutated GCP-2 to remove its chemotactic/inflammatory properties while maintaining its anabolic properties provides a novel protective agent that reduces pain and structural damage when introduced to joints in a mouse model of post-traumatic OA. This laboratory has made important discoveries in the field and has made good use of their established in vitro and in vivo models in this work. The medical impact is high because there is no approved pharmacological invention that can address both pain and joint damage. Comments below need to be addressed to improve the manuscript.

Specific comments:
1. Abstract:
a. In the last line, it is unclear what is meant by "without inducing synovitis". Did you mean "without reducing synovitis" in the case that the model induced synovitis and GCP-2-T had no effect? In the Results section, it is stated that there was "no difference in synovial thickness, but it is unclear without looking at the Fig. S3C whether or not the model increased synovial thickness. In fact, Fig. S3C is not help in this regard because there is no comparison between the MLI joints and the sham controls.
b. In the last sentence, it would be more accurate to state that GCP-2 reduced "cartilage loss", rather than "cartilage breakdown", since its effects are shown here to be anabolic.
c. Is there a concluding take-home message that could be stated here?

2. Introduction: In the last sentence, the capacity of GCP-2 to "improve the outcome of experimental osteoarthritis in mice" is vague. Could it be more specifically stated as "abrogate pain, as well as cartilage loss, osteophyte formation and bone changes experimental osteoarthritis in mice"?

3. Results:
a. Figure 1: It is unclear why dark field images are not shown for immunolocalization of GCP- in all panels in 1B and 1D. Except

In the high-power magnification in 1B, it is difficult to see the cellular localization in relation to DAPi. It looks more nuclear in the low-power images.
b. The authors might consider making more succinct the introductory sentences and concluding interpretations in each section of the Results, as this information may be better used to enhance the Discussion (unless this is the journal style).
c. Figure 2: The text indicates that human C28/I2 cells were used in Fig. 2B, but the figure is labeled with murine C3H10T1/2 cells and the legend indicates that they were cultured in micromass. Please correct and clarify.
d. In the section of the Results describing Figure 3, there are two numbered references cited, (24) and (29), which must have been left from a previous version, since the reference list is by Author and Date.
e. Please indicate that Safranin O-stained sections were used for OARSI scoring and determination of osteophyte size in Figure 5. Was osteophyte maturation also examined? This would provide evidence of effects on ossification in the osteophytes. Note that the differences in the representative sections in 5F and 5H are difficult to see. Why was Fast green not included in the staining protocol?
f. For the mouse OA model, there is a scheme in panel 5A showing the time course, but please indicate in the text that OARSI scoring was done 10 weeks after surgical induction of the OA. Why was this time point chosen? Were time course experiments performed previously to determine that this was the best time to look at structural changes.
g. Please indicate that bone density was measured by microCT as BV/TV in Fig. 3SA and 3SB.
h. In the sentence describing results in Fig. 5E and 5F, would it be more accurate to state that there was "less cartilage loss", rather than "less cartilage breakdown", because the latter implies an active degradation mechanism?
i. See note above about better explaining the meaning of "no difference in synovial thickness was detected" with regard what is being compared.

4. The paper explained: At end of RESULTS, it would be more accurate to state that "we reduced pain and improved cartilage integrity".

5. Editing: Please check carefully throughout the manuscript for several instances of missing words.

Referee #3 (Comments on Novelty/Model System for Author):

Human OA (structural changes as well as pain) are well modelled in the meniscus-ligament injury surgical model.

Referee #3 (Remarks for Author):

This manuscript describes a novel and innovative approach to potentially treating osteoarthritis. The results are thus of considerable importance as there are currently no approved disease-modifying therapies for this highly prevalent and disabling disease. While most researchers are focusing on inhibiting catabolic proteinases or enhancing the activity of anabolic growth factors, Caxaria et al. have taken the novel approach of employing the anabolic properties of the chemokine GCP-2. This is based on the considerable previous experience of Dell'Accio in osteoarthritis pathogenesis research generally and in CXCR1/2 in this context in particular. The added expertise of Day in the biochemistry of GAG/protein interactions enabled development of novel GCP-2 mutants that reduce both joint damage and pain. As the manuscript argues, this has the potential to lead to game-changing therapies for osteoarthritis.

My main questions are as follows:
1. Is expression of GCP-2 and GCP-2-T the same in vivo? This is critical for comparing their in vivo effects.

2. A central conclusion is that while GCP-2 causes inflammation, GCP-2-T does not. This is supported by in vitro data (Fig 4), but not by any in vivo data. Fig S3C shows no difference in synovial thickening between treated groups - is there no thickening relative to unoperated animals or do all groups show increased but indistinguishable synovitis? Differential effects on inflammation in vivo (specifically in the OA model) should be further validated i.e. show that GCP-2 but not GCP-2-T is able to increase cellular invasion into the synovium or cytokine expression in joints (for example by ELISA, qPCR, histology, or RNAScope).

3. Another central conclusion is that GCP-2-T reduces pain but GCP-2 does not. However, the extent of pain in the murine OA model seems minimal, with a change in weightbearing of 2% between "painful" and "non-painful" (Fig 5B). In comparison, Von Loga et al. (DOI: 10.1136/annrheumdis-2018-214489) show a delta of 20% in a similar meniscal surgery model.

4. Figure 4G-H confirms that GCP-2-T retains GCP-2's ability to stimulate matrix anabolism and aggrecan expression in C28/I2 micromass cultures, but effects on hypertrophic differentiation are not shown. Experiments such as those shown in Fig 3 for wild-type GCP-2 would address this.

5. GCP-2-T activation of Akt phosphorylation should be shown in more detail i.e. qualify n>3 and show effects on target genes.

While the text (p10, first line of paragraph 3) states that "GCP-2-T was still able to induce AKT phosphorylation in chondrocytes", Fig 4F shows Akt phosphorylation in C28/I2 cells. It would be good to verify these effects in primary HAC or murine chondrocytes, since this central to the mechanism proposed.

I also have following minor suggestions to strengthen the manuscript:

1. page 4: Is it fair to say "cartilage has a very low turnover'? Some elements, such as aggrecan, can be turned over quite rapidly, especially in OA. I think the sentence reads fine without this statement.

2. page 5: Define abbreviation of GCP-2.

3. page 7 and Figure 2: The text on Figure 2 indicates that C3H10T1/2 were used for this experiment, while the text and figure legend indicates that C28/I2 cells were used - which were used? If C28/I2 cells were used in Fig 2B, why did the authors switch to using C3H10T1/2 from Fig 2C?
How long were micromasses cultured before analysis?
How much GCP-2 was added in Fig 2C-D?
Fig 2F should read "toluidine blue" not "toluiding blue".

4. page 7: The data support a role for GCP-2 in promoting cartilage anabolism, but effects on catabolism are not shown, so it would be better to rephrase the conclusion to "Taken together these data suggest that the increase in ECM induced by GCP-2 is due to an anabolic effect".

5. page 8: How much GCP-2 was added in Fig 3A-H, and how much thyroid hormone in Fig 3C-E?
Fig 3F-H: Include control (non-osteogenic) medium as well, to enable assessment of the effect size for inhibition of osteogenesis. Thus is especially important for G and H where Y axis doesn't start near 0.

7. In Fig 4C, please add NaCl concentration to the elution profile of GPC-2 proteins from heparin Sepharose.

8. page 10: Please justify why pre B cells (300-19) were selected to analyse effects on chemotaxis (Fig 4D) and transendothelial migration (Fig 4E) in vitro. I appreciate that neutrophils are not a tractable in vitro model, but in the context of OA, migration of CXCR2-expressing monocytes into the synovium would have a clearer mechanistic relevance.

9. In Fig 4F-I, which Akt antibody was used? How much GCP-2 (WT and mutant) was added and for how long? It is interesting that GCP-2-T looks to activate pAkt more than the WT - is this statistically significant?

10. page 17: Folding of recombinant GCP-2 was assessed by 1D NMR. Given that CXCL6 has 2 disulfide bonds (Uniprot annotation), was disulfide bond formation also confirmed on reducing/non-reducing SDS-PAGE?

11. In Fig S2: Please add units (nM?) to the X-axis of chemotaxis and TEM figures.

Summary for the editor and reviewers:

We thank the reviewers and the editorial office for appraising our manuscript and for the constructive criticism.

In this revised version, we have generated 12 new experimental datasets:

1. pAKT western quantification (Fig 4F)
2. PCR COL2A1 in non-osteogenic medium (Fig 4I)
3. PCR Col10 in non-osteogenic medium (Fig 4J)
4. Assessment of osteophyte maturity at end point in osteoarthritis and quantification (Fig 5I)
5. SDS-PAGE GCP-2 and GCP-2-T in reducing and non-reducing conditions (EV 2C)
6. Average synovial thickness 4 days after injection of GFP, GCP-2 or GCP-2-T adenovirus (EV 2G)
7. Knee swelling 4 days after injection of GFP, GCP-2 or GCP-2-T adenovirus (EV 2H)
8. pAKT staining and quantification 4 days after injection of GFP, GCP-2 or GCP-2-T adenovirus (EV 4A)
9. Collagen type II staining and quantification at end point in osteoarthritis and quantification (EV 4B)
10. Collagen type X staining and quantification at end point in osteoarthritis and quantification (EV 4C)
11. Aggrecan neo-epitope NITEGE quantification at end point in osteoarthritis and quantification (EV 4D)
12. TUNEL assay at end point in osteoarthritis and quantification (EV 4E)

As advised by the editor, we have prioritised "a better characterization of the mouse model of osteoarthritis in absence or presence of GCP-2-T […] including evaluation of cartilage anabolism, catabolism, chondrocyte apoptosis, AKT signaling, and inflammation".

Referee #1 (Comments on Novelty/Model System for Author):

***This manuscript is of good quality. The only limitation concerns the use of adenoviral vectors to deliver the cytokine that prevents OA. It would be interesting to test a different way to administer this molecule, a way that could/should be more clinically relevant*.**

We thank the reviewer for praising the quality of the manuscript. Of course, we acknowledge that the delivery of a suitably stabilized recombinant molecule would be more clinically relevant. Delivering such data, however, may entail improving the PK profile and establishing the appropriate dose and regime for the in vivo experiments. This is not doable within the time frame allowed for revisions. We have added a discussion point to acknowledge this limitation.

Referee #1 (Remarks for Author):

1- The relative expression of additional anabolic markers (e.g. COL2) and catabolic markers (e.g. MMP13, ADAMTS) should be evaluated in all relevant experiments performed in this study (Fig. 1 to 4). ColX mRNA expression should be evaluated in addition to Runx2 as marker of hypertrophy (Fig. 3A). Additional representative

photographic illustrations could be added to the figures wherever the data are represented only as graphs.

We thank the reviewer for the suggestion. While some of the data were already present in the old version of the manuscript (COL10A1 was already in figure 3E) we have now added COL2A1 and COL10A1 in the absence of an osteogenic stimulus (Fig 4I-J). These new important data show that the capacity of GCP-2 to suppress COL10A1 is specific for osteogenic conditions and therefore it is not a general inhibitory effect on baseline expression or perhaps the lack of downregulation is due to floor effect (difficult to downregulate already low values) at baseline level of osteogenic markers. Yet, within the same experiment in non-osteogenic conditions, GCP-2 upregulated COL2A1 demonstrating the expected biological activity.

2- Figure 5, hypertrophic (COLX), anabolic (ACAN or COL2) and catabolic (MMP13) markers should be evaluated by immunostaining on sections. It would be also interesting to assess neutrophil infiltration, apoptosis and AKT activation (Phospho-AKT) in vivo after GPC-2 and GPC-2-T "treatment".

We thank the reviewer for the suggestion. We have performed and quantified the staining for Collagen type II and type X, for the Aggrecan neo-epitope NITEGE and of the number of apoptotic cells (TUNEL staining) on the MLI experiment (Fig EV4). As for MMP-13, although this staining worked for “positive control” slides decalcified with 10% EDTA, it did not work after 3 attempts with the slides obtained from this experiment which were decalcified with Decal. So, regrettably, we cannot add MMP-13 data, however, we have now added a staining for the aggrecan neo-epitope NITEGE in Fig Extended view 4. In addition, we have performed and quantified the staining for pAKT in mice injected intra-articularly with the adenoviruses and killed after 4 days (Fig EV4A). We chose this early time point in non-osteoarthritic mice for pAKT and for neutrophil infiltration because of the transient nature of this phosphorylation event and the short life of neutrophils. Also, we chose to measure pAKT in mice without osteoarthritis to avoid the confounder of AKT phosphorylation caused by osteoarthritis pathology. As for MMP-13, although this staining worked for “positive control” slides decalcified with 10% EDTA, it did not work after 3 attempts, with the slides obtained from this experiment which were decalcified with Decal. So, regrettably, we cannot add MMP-3 data; Finally, we have added other parameters relevant to inflammation to the data assessing neutrophil infiltration, such as average synovium thickness (Fig EV 2G) and knee swelling (Fig EV 2H) four days after injection of either GCP-2 or GCP-2-T in mice without osteoarthritis to study the effects of GCP-2 and GCP-2-T in isolation.

2.a. It is important to show and quantify cartilage degradation at week 5, prior adenoviral injections. Does GPC-2-T really revert the defects or does it only limit/stop further destructions taking place between week 5 and week 10 in the control GFP group?

Unfortunately, we do not have the 5-week time point, however, GCP-2 increased extracellular matrix production of human cartilage organoids implanted in nude mice as shown in Fig 2F-G, thereby suggesting that GCP-2 has an anabolic effect. This important point has now been added to the discussion. We also believe that a time point before initiating treatment is perhaps not necessarily addressing the point because, despite the “treatment”, joint instability would still persist, and would induce further cartilage breakdown. Therefore, perhaps even if there was genuine regeneration, this may not have resulted in an improvement of the OARSI score.

3- It would be interesting to evaluate whether systemic administration of GPC-2-T could be used to achieve the same effects that are observed after intra-articular injections of adenovirus. What is the half-life of this molecule? Could it be encapsulated in extracellular vesicles? The possibility of using this molecule in clinic should be better discussed in the manuscript.

This would be interesting, but before these experiments can be done, we would need to generate a recombinant molecule stabilized to optimize pharmacokinetics and a series of experiments should be performed to optimize dose and frequency of administration. While we have already generated constructs with modifications aiming to prolong half-life, these experiments exceed the time allowed for revisions. Taking on board the suggestion of the reviewer, we have now improved the discussion of this point and the steps needed to take this molecule to clinical fruition.

Referee #2 (Remarks for Author):

We thank the reviewer for praising the novelty and quality of this study.

1. Abstract:
a. In the last line, it is unclear what is meant by "without inducing synovitis". Did you mean "without reducing synovitis" in the case that the model induced synovitis and GCP-2-T had no effect? In the Results section, it is stated that there was "no difference in synovial thickness, but it is unclear without looking at the Fig. S3C whether or not the model increased synovial thickness. In fact, Fig. S3C is not help in this regard because there is no comparison between the MLI joints and the sham controls.

Since GCP-2 is a chemokine with pro-inflammatory properties, we feared that its intra-articular injection would have caused synovitis. Therefore, the fact that GCP-2-T did not *per se* induce synovitis was reassuring.

We were not suggesting that GCP-2-T reduced synovitis because overt synovitis is not a feature of the MLI model at this late time point and it was not possible therefore to reach this conclusion. The sentence referred to the data in figure S3C (now EV 3C). The sentence in results has been modified accordingly to make it clearer and the one in the abstract has been removed to avoid confusion.

b. In the last sentence, it would be more accurate to state that GCP-2 reduced "cartilage loss", rather than "cartilage breakdown", since its effects are shown here to be anabolic.

We agree with the reviewer and we have modified the text accordingly.

c. ***Is there a concluding take-home message that could be stated here?***

Thank you. We have added a concluding sentence

2. Introduction: In the last sentence, the capacity of GCP-2 to "improve the outcome of experimental osteoarthritis in mice" is vague. Could it be more specifically stated as "abrogate pain, as well as cartilage loss, osteophyte formation and bone changes experimental osteoarthritis in mice"?

Thank you for the suggestion. We have changed the sentence accordingly.

3. Results:
a. Figure 1: It is unclear why dark field images are not shown for immunolocalization of GCP- in all panels in 1B and 1D. Except In the high-power magnification in 1B, it is difficult to see the cellular localization in relation to DAPi. It looks more nuclear in the low-power images.

1B is immunohistochemistry, not immunofluorescence and therefore there is no DAPI. For C, unfortunately we did not take dark field images, however, in the revised manuscript, we have added dotted lines to separate cartilage from bone, labelling for the different anatomical structures, and an inset to show a higher magnification of the composite to show the localization in relation to DAPI. For D, which is human cartilage, we do not have DAPI, but, we believe, the dark field image sufficiently details the morphological aspects.

b. The authors might consider making more succinct the introductory sentences and concluding interpretations in each section of the Results, as this information may be better used to enhance the Discussion (unless this is the journal style).

We believe that the reviewer refers to the aspects pertaining to developmental biology. We agree with the suggestion, and we have modified the text accordingly.

c. Figure 2: The text indicates that human C28/I2 cells were used in Fig. 2B, but the figure is labeled with murine C3H10T1/2 cells and the legend indicates that they were cultured in micromass. Please correct and clarify.

Thank you for pointing out this error for which we apologise. Primary chondrocytes and C28I/2 micromasses are more differentiated and therefore are best suited to test loss of function, whereas C3H10T1/2 are very undifferentiated (unless treated with BMP-2) and therefore best suited for gain of function but underestimate the loss of function because they start from very low levels of differentiation genes and extracellular matrix. Therefore, we decided to use primary chondrocytes and C28I/2 for the siRNA experiments and C3H10T1/2 for the gain of function studies. Unfortunately, when putting together the figures, we accidentally used data obtained with C28I/2 instead of the ones with C3H10T1/2. We have now corrected the mistake and a much bigger difference is shown in C28I/2 cells in micromass.

d. In the section of the Results describing Figure 3, there are two numbered references cited, (24) and (29), which must have been left from a previous version, since the reference list is by Author and Date.

We apologize for the mistake, which has been corrected. Thank you for pointing it out.

e. Please indicate that Safranin O-stained sections were used for OARSI scoring and determination of osteophyte size in Figure 5. Was osteophyte maturation also examined? This would provide evidence of effects on ossification in the osteophytes. Note that the differences in the representative sections in 5F and 5H are difficult to see. Why was Fast green not included in the staining protocol?

Thank you for pointing this out. We have now mentioned that we used Safranin O for OARSI scoring. We do not routinely add fast green because it adds considerable variability in the washing steps and sometimes even artefacts. The representative sections used for display are the ones that are closest to the median in the graph. Because of the nature of the OARSI score, the grading is largely dependent on the depth of the lesion with reference to the tidemark and its extension. To facilitate the assessment of the differences between the sections shown we have now indicated the tidemark with open arrows.

Taking on board the suggestion of the reviewer, we have now scored the osteophyte maturity and shown this in Fig 5I.

f. For the mouse OA model, there is a scheme in panel 5A showing the time course, but please indicate in the text that OARSI scoring was done 10 weeks after surgical induction of the OA. Why was this time point chosen? Were time course experiments performed previously to determine that this was the best time to look at structural changes.

Thank you. We have now indicated that the OARSI score was done 10 weeks after MLI in the main text and in the legend.

The 10 week point was chosen based on the report by Sampson et al. (Sampson *et al*, 2011) who originally described the model and from our own experiments as published in (Thorup *et al*, 2020). At the chosen time point (10 weeks), most sections score between 3 and 4, therefore avoiding the ceiling effect of very late time points when hardly any cartilage is left (Sampson *et al*, 2011). In addition, pain – as measured by incapacitance - becomes a feature of this model starting from week 7 as shown in this manuscript and in our previous publication (Thorup *et al*, 2020).

g. Please indicate that bone density was measured by microCT as BV/TV in Fig. 3SA and 3SB.

Thank you for pointing this out. We have modified the figure/legend accordingly.

h. In the sentence describing results in Fig. 5E and 5F, would it be more accurate to state that there was "less cartilage loss", rather than "less cartilage breakdown", because the latter implies an active degradation mechanism?

We agree and have modified the sentence.

4. ***At end of RESULTS, it would be more accurate to state that "we reduced pain and improved cartilage integrity".***

We have taken on board the suggestion and modified the text accordingly. Thank you.

5. Editing: Please check carefully throughout the manuscript for several instances of missing words.

Thank you. We have thoroughly reviewed the revised manuscript to avoid such errors.

Referee #3

We are grateful to the reviewer for praising the relevance of the study and the quality of the manuscript.

1. Is expression of GCP-2 and GCP-2-T the same in vivo? This is critical for comparing their in vivo effects.

Although preliminary data obtained by immunostaining did not show evidence of different expression of GCP-2 and GCP-2-T, this issue cannot be easily addressed because GCP-2 is endogenously expressed by mouse cartilage, there is no tag on the overexpressed GCP-2 construct to distinguish it from the endogenous, and, of course, because it is a soluble molecule. Having said that, the adenoviral constructs for wild-type and mutant GCP-2 are identical except for the mutation introduced (GCP-2-T) and the study is well-powered to account for variability of injection. In addition, the *in vitro* studies, performed with recombinant proteins, support the findings from in vivo experiments.

2. A central conclusion is that while GCP-2 causes inflammation, GCP-2-T does not. This is supported by in vitro data (Fig 4), but not by any in vivo data. Fig S3C shows no difference in synovial thickening between treated groups - is there no thickening relative to unoperated animals or do all groups show increased but indistinguishable synovitis? Differential effects on inflammation in vivo (specifically in the OA model) should be further validated i.e. show that GCP-2 but not GCP-2-T is able to increase cellular invasion into the synovium or cytokine expression in joints (for example by ELISA, qPCR, histology, or RNAScope).

This issue is in fact already addressed in Figure 4I (now) 4K, and EV 2G) in which we injected intraarticularly either GCP-2 or GCP-2-T adenovirus and we counted the number of neutrophils within the intercondylar space four days later. These data showed that wild type GCP-2, but not GCP-2-T induced neutrophil infiltration within the joint. We chose an early time point because neutrophils are short-lived cells. Also, this early time point enables us to avoid potential confounders deriving from the different outcome of osteoarthritis following the injection of GCP-2 and GCP-2-T.

As noted above in our answers to Reviewer #2, instability-induced osteoarthritis, at least in its late time points, is not associated with overt synovitis, although local upregulation of inflammatory molecules has been described as part of the osteoarthritis pathology. Therefore no overt synovitis was expected.

3. Another central conclusion is that GCP-2-T reduces pain but GCP-2 does not. However, the extent of pain in the murine OA model seems minimal, with a change in weightbearing of 2% between "painful" and "non-painful" (Fig 5B). In comparison, Von Loga et al. (DOI: 10.1136/annrheumdis-2018-214489) show a delta of 20% in a similar meniscal surgery model.

Several differences in methodology/analysis explain this apparent discrepancy:

- In our experiments pain is already detectable at week 7 and is measured until the end of the experiment at week 10. In Von Loga's model, which, surgically, is indeed similar but not identical (we also resect the medial collateral ligament) pain is only detectable from week 10. Therefore, if the Von Loga experiment had been stopped like ours at week 10, there would have been no detection of pain at all.
- In Von Loga's data, incapacitance is expressed in % to "normal"; that is, 50% body weight on the affected limb would be expressed as 100%. Therefore, differences appear doubled compared to our data where we show the percent of body weight on the operated limb.
- Small differences in the protocol for taking incapacitance data often result in sizable differences in the final data.
- Mice are extremely sensitive to noise levels, smell (for instance even the odour of male investigators rather than female, inhibits pain behaviour (Sorge *et al*, 2014), husbandry conditions etc. Therefore, it is difficult to compare different experiments performed by different investigators and in different environments.

For all these reasons one cannot compare experiments performed by different investigators, with different protocols, time points and conditions.

Nevertheless, despite the "small" effect size intrinsic to the model the effect size of the injection of GCP-2-T is large because it restores 50% weight bearing.

4. Figure 4G-H confirms that GCP-2-T retains GCP-2's ability to stimulate matrix anabolism and aggrecan expression in C28/I2 micromass cultures, but effects on hypertrophic differentiation are not shown. Experiments such as those shown in Fig 3 for wild-type GCP-2 would address this.

In response to this, we performed PCR in non-osteogenic conditions. In non-osteogenic conditions, however, neither GCP-2 nor GCP-2-T suppress the baseline levels of COL10A1 (now Fig 4J). The lack of downregulation of baseline levels are likely due to the already low baseline levels (floor effect).

5. GCP-2-T activation of Akt phosphorylation should be shown in more detail i.e. qualify n>3 and show effects on target genes. While the text (p10, first line of paragraph 3) states that "GCP-2-T was still able to induce AKT phosphorylation in chondrocytes", Fig 4F shows Akt phosphorylation in C28/I2 cells. It would be good to verify these effects in primary HAC or murine chondrocytes, since this central to the mechanism proposed.

We agree with the reviewer and we have now quantified data from 5 independent experiments *in vitro* (Fig 4F). In fact, we have gone further and confirmed AKT phosphorylation also *in vivo* (Fig. EV 4A). For this experiment we performed quantification 4 days after intra-articular injection of GFP, GCP-2 or GCP-2-T adenovirus. This experiment confirmed increased number of cells with phosphorylation of AKT *in vivo* following injection of either GCP-2 or GCP-2-T, compared to GFP.

Minor suggestions

1. page 4: Is it fair to say "cartilage has a very low turnover'? Some elements, such as aggrecan, can be turned over quite rapidly, especially in OA. I think the sentence reads fine without this statement.

We agree and we have removed the sentence.

2. page 5: Define abbreviation of GCP-2.

Thank you – have done

3. page 7 and Figure 2: The text on Figure 2 indicates that C3H10T1/2 were used for this experiment, while the text and figure legend indicates that C28/I2 cells were used - which were used? If C28/I2 cells were used in Fig 2B, why did the authors switch to using C3H10T1/2 from Fig 2C?

This is an error in figure 2B which for which we do apologise and which we have now corrected (as described in our response to reviewer #2).

How long were micromasses cultured before analysis?
Three days. Thank you, this has been added to methods and figure legends.

How much GCP-2 was added in Fig 2C-D?
100ng/ml. This has been added to methods and figure legends.

Fig 2F should read "toluidine blue" not "toluiding blue".
We apologise for the mistake which we have now corrected.

4. page 7: The data support a role for GCP-2 in promoting cartilage anabolism, but effects on catabolism are not shown, so it would be better to rephrase the conclusion to "Taken together these data suggest that the increase in ECM induced by GCP-2 is due to an anabolic effect".

We believe the referee refers to the statement following the reference to Fig 2E. This panel shows that when adding GCP-2 to mature chondrocytes there is no difference in the release of GAGs into the medium. Therefore, the increase of the GAG retained within the micromasses in Figure 2C-D cannot be attributed to reduced catabolism, because, in that case, there would have been reduced release of GAGs into the medium. Instead, in Fig 2E, if anything, there is a trend towards an increase, which is in keeping with an increase in the substrate. We hope that this has clarifies the sentence, which we have now changed as suggested.

5. page 8: How much GCP-2 was added in Fig 3A-H, and how much thyroid hormone in Fig 3C-E?

Both 100 ng/ml. We have updated the figures accordingly. Thank you.

Fig 3F-H: Include control (non-osteogenic) medium as well, to enable assessment of the effect size for inhibition of osteogenesis. Thus is especially important for G and H where Y axis doesn't start near 0.

Unfortunately, we did not include the non-osteogenic medium as a control at the time the experiment was performed, however, previous experiments in our laboratory (Dell'Accio et. al 2003) showed that mineralization and alizarin staining in normal conditions is negligible. Additionally, of course it would have been ideal to have such a control. However, what really is essential in this experiment is the effect of GCP-2 on mineralization. If chondrocytes or chondrogenic cell lines calcified spontaneously without osteogenic medium, the experiment would still have been valid.

7***. In Fig 4C, please add NaCl concentration to the elution profile of GPC-2 proteins from heparin Sepharose.***

We have amended the figure as indicated.

8. page 10: Please justify why pre B cells (300-19) were selected to analyse effects on chemotaxis (Fig 4D) and transendothelial migration (Fig 4E) in vitro. I appreciate that neutrophils are not a tractable in vitro model, but in the context of OA, migration of CXCR2-expressing monocytes into the synovium would have a clearer mechanistic relevance.

This is a stable cell line engineered to overexpress CXCR2. Therefore, it affords higher consistency of results and reproducibility than primary cells. Relevance was subsequently shown *in vivo* with migration of neutrophils within the joint (Fig 4K and EV 2F).

9. In Fig 4F-I, which Akt antibody was used? How much GCP-2 (WT and mutant) was added and for how long? It is interesting that GCP-2-T looks to activate pAkt more than the WT - is this statistically significant?
Phospho-Akt (Ser473) Antibody #9271 (CST) 1:200. IgG2b Dako 1:200. One hundred ng/ml GCP-2 was added. *In vitro,* there was no statistically significant difference between GCP-2 and GCP-2-T (Fig 4F). *In vivo*, however, indeed GCP-2-T activated AKT phosphorylation to a higher extent than wild type GCP-2 (Fig EV 4A). This is most likely due to the fact that GCP-2-T, being unable to bind to GAGs – highly abundant in cartilage and synovial fluid – might be more readily bioavailable. We decided not to make a big point out of it because a lot more data (including time course, proteoglycan depletion etc.) would be required to make this point convincing enough, but, following the lead of this reviewer, we have added a comment in discussion.

10. page 17: Folding of recombinant GCP-2 was assessed by 1D NMR. Given that CXCL6 has 2 disulfide bonds (Uniprot annotation), was disulfide bond formation also confirmed on reducing/non-reducing SDS-PAGE?

We have analysed the WT and mutants under reducing (R) and non-reducing (NR) conditions on SDS-PAGE. There is a difference in mobility on SDS-PAGE for the R and NR samples of ~2-kDa, where this is identical for WT and all mutants, i.e., being consistent with the presence of disulfide bonds. Moreover, the 1D NMR (Fig S2 B) indicates that the mutants have a WT structure, which would not be the case if disulfide bonds were absent or differently organised.

11. In Fig S2: Please add units (nM?) to the X-axis of chemotaxis and TEM figures.

It is nM. We apologise for the oversight and have corrected accordingly.

References

Sampson ER, Beck CA, Ketz J, Canary KL, Hilton MJ, Awad H, Schwarz EM, Chen D, O'Keefe RJ, Rosier RN, *et al* (2011) Establishment of an index with increased sensitivity for assessing murine arthritis. *J Orthop Res* 29: 1145–1151

Sorge RE, Martin LJ, Isbester KA, Sotocinal SG, Rosen S, Tuttle AH, Wieskopf JS, Acland EL, Dokova A, Kadoura B, *et al* (2014) Olfactory exposure to males, including men, causes stress and related analgesia in rodents. *Nat Methods* 11: 629–632

Thorup A-S, Strachan D, Caxaria S, Poulet B, Thomas BL, Eldridge SE, Nalesso G, Whiteford JR, Pitzalis C, Aigner T, *et al* (2020) ROR2 blockade as a therapy for osteoarthritis. *Science Translational Medicine* 12: eaax3063

23rd Sep 2022

Dear Prof. Dell'Accio,

Thank you for the submission of your manuscript to EMBO Molecular Medicine. We have now received the enclosed reports from referees #1 and #3. As you will see, while referee #3 is satisfied with the revisions, referee #1 recognizes the significant work that has been done, but also raises some remaining issues that should be addressed before further consideration.
I further cross-commented with referee #1, and we concluded that the most important point would be to demonstrate that GCP-2-T induces an anabolic response in vivo.
Referee #1 suggested:
"The most straightforward experiment the authors could do is to purchase a batch of wildtype mice of the right age, perform meniscus-ligament injury (MLI) and treat with GCP-2-T for a short time (shorter than in their initial protocol), and look at COL2 and COLX (any anabolic and catabolic chondrogenic markers would be OK) by immunostaining or in situ hybridization. The issue is the time required to do this. The authors wait for 5 weeks after MLI before they start treating their mice. This means the actual in vivo experiment and analysis would require maybe ~7-8 weeks in total. Hence this would delay the publication process significantly. The alternative would be to use wildtype mice and not do MLI, but simply treat them with GCP-2-T for a couple of weeks (or whatever the authors think might work) to show that the compound stimulates cartilage anabolism in vivo. This may require only ~3-4 weeks total."

We would therefore like you to revise the manuscript further and address this point. As EMBO Press usually encourages one single round of revisions, please be aware that this will be the last chance for you to address the referee's concerns.

Moreover, please address the following editorial issues:

1/ Manuscript text:
- Please accept the changes and only keep in track changes mode any new modifications.
- We note that there are currently 3 first authors and 3 last authors on the manuscript. Is that correct? In particular, for senior authors, do you confirm equal contribution of these 3 people, able to take full responsibility for the paper and its content? While there is no limit per se, 3 is rare, and may not reflect as intended to the community.
- Keywords: please remove CXCR2 from the keywords as indicated in your email.
- Material and methods:
o We note that you indicate in several instances "as previously described". Please make sure to nevertheless provide a brief description of the methods, particularly if the references are not publicly available.
o Please indicate the origin of the cells, the culture conditions, whether they were authenticated and tested for mycoplasma contamination.

2/ Figures:
- Please check again the number of data points in the figures vs. the n= indicated in the legends (i.e. Fig. 2B, 2E, Fig. 4D,E, I, J, etc)
- Please complete the legend of Fig. 2G (right panel not mentioned).

3/ Every published paper now includes a 'Synopsis' to further enhance discoverability. Synopses are displayed on the journal webpage and are freely accessible to all readers. They include a short stand first (maximum of 300 characters, including space) as well as 2-5 one-sentences bullet points that summarizes the paper. Please write the bullet points to summarize the key NEW findings. They should be designed to be complementary to the abstract - i.e. not repeat the same text. We encourage inclusion of key acronyms and quantitative information (maximum of 30 words / bullet point). Please use the passive voice. Please attach these in a separate file or send them by email, we will incorporate them accordingly.

4/ As part of the EMBO Publications transparent editorial process initiative (see our Editorial at http://embomolmed.embopress.org/content/2/9/329), EMBO Molecular Medicine will publish online a Review Process File (RPF) to accompany accepted manuscripts.
This file will be published in conjunction with your paper and will include the anonymous referee reports, your point-by-point response and all pertinent correspondence relating to the manuscript. Let us know whether you agree with the publication of the RPF and as here, if you want to remove or not any figures from it prior to publication.
Please note that the Authors checklist will be published at the end of the RPF.

I look forward to receiving your revised manuscript.

Yours sincerely,

Lise Roth

Lise Roth, PhD
Senior Editor
EMBO Molecular Medicine

***** Reviewer's comments *****

Referee #1 (Remarks for Author):

Caxaria and colleagues have addressed in their revised manuscript some, not all, of the comments and concerns raised previously. The lack of clear evidence of the anabolic action of GCP-2-T in vivo constitutes one important limitation in this study. One would have expected to observe increased anabolic markers (and decreased catabolic markers) in knees treated with GCP-2-T after meniscus-ligament injury (MLI), compared to (GFP) controls. The authors seem to argue that week 10 (the end time-point) may be too late to see different expression of these markers, yet cartilage damage is prevented after GCP-2-T "treatment" at this time point, and more Col2-expressing chondrocytes (and maybe fewer hypertrophic chondrocytes) would be expected. Looking at these markers at an earlier time-points could have perhaps demonstrated more easily that GCP-2-T can be used in vivo to promote cartilage anabolism in osteoarthritis (OA), but the data are missing. A second important question that remains is to know whether GCP-2-T can induce cartilage regeneration in OA, or only prevent cartilage damage. This point has obviously important clinical implications. The authors had the experimental tools to address this point, but the data are also missing. Overall, this study is both novel an important. However, the lack of evidence that GCP-2-T promotes cartilage anabolism in vivo, or more broadly the lack of clear molecular and cellular effects (beside AKT phosphorylation at day 4) observed after GCP-2-T treatment in MLI knees, weakens to some extend the conclusion that GCP-2-T-induced cartilage anabolism protects against cartilage damage in OA.

Referee #3 (Comments on Novelty/Model System for Author):

Gold standard murine model of osteoarthritis used.

Referee #3 (Remarks for Author):

The authors have addressed many of the points raised in the initial review, and explained thoroughly where they have not been able to amend.

I suggest the following minor amendments in response to comments by Referee 3:

1. Point 1 in the initial review queried whether GCP-2 and GCP-2-T are expressed at the same levels in vivo. I take the authors' point that this is difficult to address, but suggest they add the points raised in their rebuttal to the manuscript Discussion for readers to consider.

2. Point 5 in the initial review queried whether experiments were also conducted in non-osteogenic media, and the authors responding by citing a previous study of theirs (Dell'Accio et. al 2003). Please add this reference and that statement that "mineralization and alizarin staining in normal conditions is negligible" to the manuscript Results section.

We thank the Editorial board and the reviewers for appraising our manuscript and for their constructive criticism.

Referee 1.

1. ***The reviewer comments that since at the endpoint we did not detect statistically significant differences in terms of collagen type II or collagen type X there is not convincing evidence that GCP-2 is anabolic or anti-catabolic in vivo and this decreases the value of the study. To address this point the reviewer suggests to perform short term studies to circumvent the problem of extensive cartilage breakdown affecting the detection of any anabolic effect of the treatment.***

In reply to this concern, we would make the subsequent points:

- We feel that we have already shown an anabolic effect of GCP-2 *in vivo*: in the model in which human cartilage organoids are implanted in nude mice we showed that GCP-2 treatment increased collagen type II expression at the protein level (Fig 2G) and also proteoglycan synthesis (Fig 2F). Moreover, in experiments using chondrocyte cell lines in 3D culture we showed that the GCP-2 and GCP-2-T had equivalent effects on anabolic markers, i.e., pAKT, proteoglycan deposition (Alcian Blue) and aggrecan gene expression (Fig 4F-H). We also would like to point out that this human cartilage formation model in nude mice is highly predictive of clinical outcomes in clinical trials: for instance, chondrocyte biomarkers correlated with this model (Dell'Accio *et al*, 2001) predicted the clinical outcome of patients treated with autologous chondrocyte implantation at 3 year follow up (Saris *et al*, 2009). Therefore, we feel that we have demonstrated the anabolic function of GCP-2 on cartilage in a highly clinically relevant *in vivo* model in human organoids.
- In osteoarthritis, collagen type II and aggrecan are strongly upregulated by homeostatic responses but simultaneously degraded through enzymatic degradation by metalloproteinases and aggrecanases respectively. The rate of degradation is driven by the abundance of the enzymes (upregulated in osteoarthritis) but also by the abundance of the substrate (collagen type II and Aggrecan). This was clearly demonstrated, for instance, in a prospective study where the Aggrecan degradation marker ARGS was paradoxically reduced in patients who had the highest rate of osteoarthritis progression (Larsson *et al*, 2012). Therefore, in such disease models characterized by rapid extracellular matrix turnover, any additional upregulation driven by GCP-2 is going to be difficult to detect also because it will be likely to increase the rate of degradation. In naïve mice (no osteoarthritis), we suspect that the experiment suggested will be less informative than the *in vivo* data supplied in Fig 2G and 2F. In part, because in native mouse cartilage (in the absence of osteoarthritis) the expression of type II collagen and aggrecan are already maximal and unlikely to be upregulated by GCP-2 (the ceiling effect).
- The choice of the time point is also going to be crucial and would need to be determined experimentally. Therefore, the experiments suggested would have to use a large number of experimental animals, large amounts of time and resources: expansion of the adenovirus, its validation and titration, the animal experiment itself, the laborious sample processing, sectioning and immunostaining, realistically, would require several months, high costs and dedicated personnel. In our opinion, the experiment would have limited chances of success and whatever the outcome, is going to supply information difficult to interpret and not superior to what is already available in Fig 2F and 2G.

- Finally, under the 3Rs, we would also question whether it is ethical to use additional animals for further experiments with uncertain outcome value.

In summary, having already supplied evidence of an anabolic activity for GPC-2 *in vivo* in Fig 2F-G and *in vitro* for GPC-2 and GPC-2-T (Fig 4F-H), we feel that it is not reasonable to ask us to perform a very large and expensive experiment with no guarantee that the data obtained would provide anything additional to what is already present.

2. ***In the initial review queried whether GCP-2 and GCP-2-T are expressed at the same levels in vivo. I take the authors' point that this is difficult to address, but suggest they add the points raised in their rebuttal to the manuscript Discussion for readers to consider*.**

We agree with the reviewer and we have added a paragraph in the discussion mentioning that as adenoviral GCP-2 was not tagged, it was not possible to distinguish it from endogenous GCP-2 and to verify that the expression levels were equal for GCP-2 and GCP-2-T. However, apart from the three mutated lysine residues, the adenoviral vectors were identical, produced and titrated at the same time, and the treatment was randomized within each cage. In addition, the outcomes of these in vivo studies paralleled our in vitro studies which were made with recombinant proteins at known concentration.

3. ***"Point 5 in the initial review queried whether experiments were also conducted in non-osteogenic media, and the authors responding by citing a previous study of theirs (Dell'Accio et. al 2003). Please add this reference and that statement that "mineralization and alizarin staining in normal conditions is negligible" to the manuscript Results section."***

Many thanks for the suggestion. We have added the sentence and the reference in the results section.

Additional instructions from the Editor

- Keywords: please remove CXCR2 from the keywords as indicated in your email.

Thank you. This has now been done.

- Material and methods: We note that you indicate in several instances "as previously described". Please make sure to nevertheless provide a brief description of the methods, particularly if the references are not publicly available.

We have added a brief description of the methods, particularly when at least one reference is not open access.

- Please indicate the origin of the cells, the culture conditions, whether they were authenticated and tested for mycoplasma contamination.

Thank you. This has now been indicated.

2/ Figures:
- Please check again the number of data points in the figures vs. the n= indicated in the legends (i.e. Fig. 2B, 2E, Fig. 4D,E, I, J, etc)

We apologise for the imprecisions. The numbers have now been corrected.

- Please complete the legend of Fig. 2G (right panel not mentioned).

The legend to Fig 2G reads "G Collagen 2 (Col2) immunostaining (left) and quantification (right) as % of total area". Therefore, the right panel is indicated. We spotted, however, that in Fig **4** panel H had the letter H missing. We believe that you were referring to Fig 4. We apologise for the mistake and have now corrected the figure.

3/ Every published paper now includes a 'Synopsis' to further enhance discoverability. Synopses are displayed on the journal webpage and are freely accessible to all readers. They include a short stand first (maximum of 300 characters, including space) as well as 2-5 one-sentences bullet points that summarizes the paper. Please write the bullet points to summarize the key NEW findings. They should be designed to be complementary to the abstract - i.e. not repeat the same text. We encourage inclusion of key acronyms and quantitative information (maximum of 30 words / bullet point). Please use the passive voice. Please attach these in a separate file or send them by email, we will incorporate them accordingly.

We have now supplied a synopsis.

4/ As part of the EMBO Publications transparent editorial process initiative (see our Editorial at http://embomolmed.embopress.org/content/2/9/329), EMBO Molecular Medicine will publish online a Review Process File (RPF) to accompany accepted manuscripts.
This file will be published in conjunction with your paper and will include the anonymous referee reports, your point-by-point response and all pertinent correspondence relating to the manuscript. Let us know whether you agree with the publication of the RPF and as here, if you want to remove or not any figures from it prior to publication.

We welcome the publication of the RPF. Thank you.

26th Oct 2022

Dear Prof. Dell'Accio,

Thank you for the submission of your revised manuscript to EMBO Molecular Medicine. We have now received the report from referee #1 who re-reviewed your manuscript. As you will see below, this referee is now supportive of publication, and I am therefore pleased to inform you that we will be able to accept your manuscript once the following editorial points will be addressed:

- Please discuss the points mentioned by the referee in your manuscript (last paragraph of the report).
- Please make sure you address all queries from our data editor in the Data edited MS file in track changes mode. Accept other changes and only keep in track changes mode any new modification.
- We note that you wish to keep 3 corresponding authors. Do you also confirm that there are 3 first authors, with equal contribution?

We note that you agree with the publication of the Review Process File (RPF). Please note that the Authors checklist will be published at the end of the RPF.

I look forward to receiving your revised manuscript.

With kind regards,

Lise

Lise Roth, PhD
Senior Editor
EMBO Molecular Medicine

***** Reviewer's comments *****

Referee #1 (Remarks for Author):

Caxaria and colleagues are submitting a third version of their manuscript, which presents novel and important data on the role of GPC-2-T in preventing cartilage damage, in an experimental model of osteoarthritis. In their rebuttal letter, the authors respond to the criticism concerning the lack of data demonstrating that GPC-2-T protects from cartilage degradation in vivo after meniscus-ligament injury (MLI), through a cartilage anabolic action. This criticism only concerns the data presented in figure 5 and figure EV4. It would have been indeed important to show molecular and cellular alterations induced upon GPC-2-T therapeutic regimen (other than AKT phosphorylation) that could have explained the protective effect observed on cartilage integrity in mice after MLI. These mechanistic insights (in the MLI mouse model) would have further increased the quality of this study, and would have strengthen the data presented in figure 5E-F, given the limited number of mice analyzed and the variability of the OARSI scores observed within each experimental group.

The authors argue that they have provided sufficient evidence of the anabolic effect of GPC-2 in vivo in figure 2F-G and in vitro for both GPC-2 and GPC-2-T in figure 4F-H. On could argue, however, that the model of human articular chondrocytes mixed with COS7 cells overexpressing GPC-2 (not GPC-2-T) and injected subcutaneously in nude mice differs substantially from the MLI mouse model, in which true knee joints are exposed to a therapeutic regimen of GPC-2-T (using immune-competent mice not nude mice). The authors also explain that they do not have sufficient resources to perform additional time-consuming MLI experiments in mice. It would have been interesting to investigate whether Col2 mRNA and/or protein levels were upregulated in

mice analyzed in figure EV4A (which shows increased phospho-AKT upon GPC-2-T treatment in vivo), without the need of adding more mice. The authors suspect that any up-regulation may be difficult to see in intact knee cartilage expressing already high levels of anabolic markers. This may well be true, but it was worth checking (using adjacent sections to those shown in figure EV4A).

In spite of this remaining limitation, the results collectively appear robust, and the current manuscript is of high quality. Moreover, this study is both novel and important for our scientific community, as mentioned before. The authors could perhaps simply mention in their manuscript that the human cartilage model in nude mice is highly predictive of clinical outcomes in clinical trials (as they did in their responses to the reviewers), and could briefly discuss the lack different expression of Col2, ColX, Nitege neo-epitope and apoptosis observed in figure EV4B-E.

We thank the Editorial board and the reviewers for appraising our manuscript and for their constructive criticism.

Referee 1.

1. ***The reviewer comments that since at the endpoint we did not detect statistically significant differences in terms of collagen type II or collagen type X there is not convincing evidence that GCP-2 is anabolic or anti-catabolic in vivo and this decreases the value of the study. To address this point the reviewer suggests to perform short term studies to circumvent the problem of extensive cartilage breakdown affecting the detection of any anabolic effect of the treatment.***

In reply to this concern, we would make the subsequent points:

- We feel that we have already shown an anabolic effect of GCP-2 *in vivo*: in the model in which human cartilage organoids are implanted in nude mice we showed that GCP-2 treatment increased collagen type II expression at the protein level (Fig 2G) and also proteoglycan synthesis (Fig 2F). Moreover, in experiments using chondrocyte cell lines in 3D culture we showed that the GCP-2 and GCP-2-T had equivalent effects on anabolic markers, i.e., pAKT, proteoglycan deposition (Alcian Blue) and aggrecan gene expression (Fig 4F-H). We also would like to point out that this human cartilage formation model in nude mice is highly predictive of clinical outcomes in clinical trials: for instance, chondrocyte biomarkers correlated with this model (Dell'Accio *et al*, 2001) predicted the clinical outcome of patients treated with autologous chondrocyte implantation at 3 year follow up (Saris *et al*, 2009). Therefore, we feel that we have demonstrated the anabolic function of GCP-2 on cartilage in a highly clinically relevant *in vivo* model in human organoids.
- In osteoarthritis, collagen type II and aggrecan are strongly upregulated by homeostatic responses but simultaneously degraded through enzymatic degradation by metalloproteinases and aggrecanases respectively. The rate of degradation is driven by the abundance of the enzymes (upregulated in osteoarthritis) but also by the abundance of the substrate (collagen type II and Aggrecan). This was clearly demonstrated, for instance, in a prospective study where the Aggrecan degradation marker ARGS was paradoxically reduced in patients who had the highest rate of osteoarthritis progression (Larsson *et al*, 2012). Therefore, in such disease models characterized by rapid extracellular matrix turnover, any additional upregulation driven by GCP-2 is going to be difficult to detect also because it will be likely to increase the rate of degradation. In naïve mice (no osteoarthritis), we suspect that the experiment suggested will be less informative than the *in vivo* data supplied in Fig 2G and 2F. In part, because in native mouse cartilage (in the absence of osteoarthritis) the expression of type II collagen and aggrecan are already maximal and unlikely to be upregulated by GCP-2 (the ceiling effect).
- The choice of the time point is also going to be crucial and would need to be determined experimentally. Therefore, the experiments suggested would have to use a large number of experimental animals, large amounts of time and resources: expansion of the adenovirus, its validation and titration, the animal experiment itself, the laborious sample processing, sectioning and immunostaining, realistically, would require several months, high costs and dedicated personnel. In our opinion, the experiment would have limited chances of success and whatever the outcome, is going to supply information difficult to interpret and not superior to what is already available in Fig 2F and 2G.

- Finally, under the 3Rs, we would also question whether it is ethical to use additional animals for further experiments with uncertain outcome value.

In summary, having already supplied evidence of an anabolic activity for GPC-2 *in vivo* in Fig 2F-G and *in vitro* for GPC-2 and GPC-2-T (Fig 4F-H), we feel that it is not reasonable to ask us to perform a very large and expensive experiment with no guarantee that the data obtained would provide anything additional to what is already present.

2. ***In the initial review queried whether GCP-2 and GCP-2-T are expressed at the same levels in vivo. I take the authors' point that this is difficult to address, but suggest they add the points raised in their rebuttal to the manuscript Discussion for readers to consider*.**

We agree with the reviewer and we have added a paragraph in the discussion mentioning that as adenoviral GCP-2 was not tagged, it was not possible to distinguish it from endogenous GCP-2 and to verify that the expression levels were equal for GCP-2 and GCP-2-T. However, apart from the three mutated lysine residues, the adenoviral vectors were identical, produced and titrated at the same time, and the treatment was randomized within each cage. In addition, the outcomes of these in vivo studies paralleled our in vitro studies which were made with recombinant proteins at known concentration.

3. ***"Point 5 in the initial review queried whether experiments were also conducted in non-osteogenic media, and the authors responding by citing a previous study of theirs (Dell'Accio et. al 2003). Please add this reference and that statement that "mineralization and alizarin staining in normal conditions is negligible" to the manuscript Results section."***

Many thanks for the suggestion. We have added the sentence and the reference in the results section.

11th Nov 2022

Dear Prof. Dell'Accio,

Thank you for providing your revised files. I am pleased to inform you that your manuscript is accepted for publication and is now being sent to our publisher to be included in the next available issue of EMBO Molecular Medicine!

Please note that the keywords were listed twice, and I removed the second occurrence that was after the abstract:
Kept: chemokine/chondrogenesis/CXCL6/ GCP-2/osteoarthritis
Removed: cartilage/chemokines/CXCL6/ GCP-2/osteoarthritis

Please let us know immediately if you do not agree with this choice.

Congratulations on your interesting work!

With kind regards,

Lise Roth

Lise Roth, Ph.D
Senior Editor
EMBO Molecular Medicine

Follow us on Twitter @EmboMolMed
Sign up for eTOCs at embopress.org/alertsfeeds

EMBO Press Author Checklist

Corresponding Author Name: Francesco Dell'Accio	
Journal Submitted to: Embo Molecular Medicine	
Manuscript Number: EMM-2022-16218	

USEFUL LINKS FOR COMPLETING THIS FORM
The EMBO Journal - Author Guidelines
EMBO Reports - Author Guidelines
Molecular Systems Biology - Author Guidelines
EMBO Molecular Medicine - Author Guidelines

Reporting Checklist for Life Science Articles (updated January 2022)
This checklist is adapted from Materials Design Analysis Reporting (MDAR) Checklist for Authors. MDAR establishes a minimum set of requirements in transparent reporting in the life sciences (see Statement of Task: 10.31222/osf.io/9sm4x). Please follow the journal's guidelines in preparing your manuscript.
Please note that a copy of this checklist will be published alongside your article.

Abridged guidelines for figures
1. Data
The data shown in figures should satisfy the following conditions:
- the data were obtained and processed according to the field's best practice and are presented to reflect the results of the experiments in an accurate and unbiased manner.
- ideally, figure panels should include only measurements that are directly comparable to each other and obtained with the same assay.
- plots include clearly labeled error bars for independent experiments and sample sizes. Unless justified, error bars should not be shown for technical replicates.
- if n<5, the individual data points from each experiment should be plotted. Any statistical test employed should be justified.
- Source Data should be included to report the data underlying figures according to the guidelines set out in the authorship guidelines on Data Presentation.

2. Captions
Each figure caption should contain the following information, for each panel where they are relevant:
- a specification of the experimental system investigated (eg cell line, species name).
- the assay(s) and method(s) used to carry out the reported observations and measurements.
- an explicit mention of the biological and chemical entity(ies) that are being measured.
- an explicit mention of the biological and chemical entity(ies) that are altered/varied/perturbed in a controlled manner.
- the exact sample size (n) for each experimental group/condition, given as a number, not a range;
- a description of the sample collection allowing the reader to understand whether the samples represent technical or biological replicates (including how many animals, litters, cultures, etc.).
- a statement of how many times the experiment shown was independently replicated in the laboratory.
- definitions of statistical methods and measures:
  - common tests, such as t-test (please specify whether paired vs. unpaired), simple χ2 tests, Wilcoxon and Mann-Whitney tests, can be unambiguously identified by name only, but more complex techniques should be described in the methods section;
  - are tests one-sided or two-sided?
  - are there adjustments for multiple comparisons?
  - exact statistical test results, e.g., P values = x but not P values < x;
  - definition of 'center values' as median or average;
  - definition of error bars as s.d. or s.e.m.

Please complete ALL of the questions below.
Select "Not Applicable" only when the requested information is not relevant for your study.

Materials

Newly Created Materials	Information included in the manuscript?	In which section is the information available? (Reagents and Tools Table, Materials and Methods, Figures, Data Availability Section)
New materials and reagents need to be available; do any restrictions apply?	Yes	Materials and methods, data availability section

Antibodies	Information included in the manuscript?	In which section is the information available? (Reagents and Tools Table, Materials and Methods, Figures, Data Availability Section)
For **antibodies** provide the following information:<br>- Commercial antibodies: RRID (if possible) or supplier name, catalogue number and or/clone number<br>- Non-commercial: RRID or citation	Yes	Table 2

DNA and RNA sequences	Information included in the manuscript?	In which section is the information available? (Reagents and Tools Table, Materials and Methods, Figures, Data Availability Section)
**Short novel DNA or RNA including primers, probes**: provide the sequences.	Yes	Table 1, Materials and methods

Cell materials	Information included in the manuscript?	In which section is the information available? (Reagents and Tools Table, Materials and Methods, Figures, Data Availability Section)
**Cell lines:** Provide species information, strain. Provide accession number in repository **OR** supplier name, catalog number, clone number, and/**OR** RRID.	Yes	Materials and methods
**Primary cultures:** Provide species, strain, sex of origin, genetic modification status.	Yes	Materials and methods
Report if the cell lines were recently **authenticated** (e.g., by STR profiling) and tested for mycoplasma contamination.	Not Applicable	

Experimental animals	Information included in the manuscript?	In which section is the information available? (Reagents and Tools Table, Materials and Methods, Figures, Data Availability Section)
**Laboratory animals or Model organisms:** Provide species, strain, sex, age, genetic modification status. Provide accession number in repository **OR** supplier name, catalog number, clone number, **OR** RRID.	Yes	Materials and Methods
**Animal observed in or captured from the field:** Provide species, sex, and age where possible.	Not Applicable	
Please detail **housing and husbandry conditions**.	Yes	Materials and Methods

Plants and microbes	Information included in the manuscript?	In which section is the information available? (Reagents and Tools Table, Materials and Methods, Figures, Data Availability Section)
**Plants:** provide species and strain, ecotype and cultivar where relevant, unique accession number if available, and source (including location for collected wild specimens).	Not Applicable	
**Microbes:** provide species and strain, unique accession number if available, and source.	Not Applicable	

Human research participants	Information included in the manuscript?	In which section is the information available? (Reagents and Tools Table, Materials and Methods, Figures, Data Availability Section)
If collected and within the bounds of privacy constraints report on age, sex and gender or ethnicity for all study participants.	Not Applicable	

Core facilities	Information included in the manuscript?	In which section is the information available? (Reagents and Tools Table, Materials and Methods, Figures, Data Availability Section)
If your work benefited from core facilities, was their service mentioned in the acknowledgments section?	Not Applicable	

Design

Study protocol	Information included in the manuscript?	In which section is the information available? (Reagents and Tools Table, Materials and Methods, Figures, Data Availability Section)
If study protocol has been **pre-registered, provide DOI in the manuscript**. For clinical trials, provide the trial registration number **OR** cite DOI.	Not Applicable	
Report the **clinical trial registration number** (at ClinicalTrials.gov or equivalent), where applicable.	Not Applicable	

Laboratory protocol	Information included in the manuscript?	In which section is the information available? (Reagents and Tools Table, Materials and Methods, Figures, Data Availability Section)
Provide DOI OR other citation details if **external detailed step-by-step protocols** are available.	Yes	Materials and methods, refering to Thorup et al., 2022.

Experimental study design and statistics	Information included in the manuscript?	In which section is the information available? (Reagents and Tools Table, Materials and Methods, Figures, Data Availability Section)
Include a statement about **sample size** estimate even if no statistical methods were used.	Yes	Materials and methods
Were any steps taken to minimize the effects of subjective bias when allocating animals/samples to treatment (e.g. **randomization procedure**)? If yes, have they been described?	Yes	Materials and methods
Include a statement about **blinding** even if no blinding was done.	Yes	Materials and methods
Describe **inclusion/exclusion criteria** if samples or animals were excluded from the analysis. Were the criteria pre-established?<br><br>If sample or data points were omitted from analysis, report if this was due to attrition or intentional exclusion and provide justification.	Yes	Materials and methods
For every figure, are **statistical tests** justified as appropriate? Do the data meet the assumptions of the tests (e.g., normal distribution)? Describe any methods used to assess it. Is there an estimate of variation within each group of data? Is the variance similar between the groups that are being statistically compared?	Yes	Figure legends

Sample definition and in-laboratory replication	Information included in the manuscript?	In which section is the information available? (Reagents and Tools Table, Materials and Methods, Figures, Data Availability Section)
In the figure legends: state number of times the experiment was **replicated** in laboratory.	Yes	Figure legends
In the figure legends: define whether data describe **technical or biological replicates**.	Yes	Figure legends

Ethics

Ethics	Information included in the manuscript?	In which section is the information available? (Reagents and Tools Table, Materials and Methods, Figures, Data Availability Section)
Studies involving **human participants**: State details of **authority granting ethics approval** (IRB or equivalent committee(s), provide reference number for approval.	Yes	Materials and methods
Studies involving **human participants**: Include a statement confirming that **informed consent** was obtained from all subjects and that the experiments conformed to the principles set out in the WMA Declaration of Helsinki and the Department of Health and Human Services Belmont Report.	Yes	Materials and methods
Studies involving **human participants:** For publication of **patient photos**, include a statement confirming that consent to publish was obtained.	Not Applicable	
Studies involving experimental **animals**: State details of **authority granting ethics approva**l (IRB or equivalent committee(s), provide reference number for approval. Include a statement of compliance with ethical regulations.	Yes	Materials and methods
Studies involving **specimen and field samples:** State if relevant **permits** obtained, provide details of authority approving study; if none were required, explain why.	Not Applicable	

Dual Use Research of Concern (DURC)	Information included in the manuscript?	In which section is the information available? (Reagents and Tools Table, Materials and Methods, Figures, Data Availability Section)
Could your study fall under dual use research restrictions? Please check biosecurity documents and list of **select agents and toxins** (CDC): https://www.selectagents.gov/sat/list.htm	Not Applicable	
If you used a select agent, is the security level of the lab appropriate and reported in the manuscript?	Not Applicable	
If a study is subject to dual use research of concern regulations, is the name of the **authority granting approval and reference number** for the regulatory approval provided in the manuscript?	Not Applicable	

Reporting

The MDAR framework recommends adoption of discipline-specific guidelines, established and endorsed through community initiatives. Journals have their own policy about requiring specific guidelines and recommendations to complement MDAR.

Adherence to community standards	Information included in the manuscript?	In which section is the information available? (Reagents and Tools Table, Materials and Methods, Figures, Data Availability Section)
State if relevant guidelines or checklists (e.g., **ICMJE, MIBBI, ARRIVE, PRISMA**) have been followed or provided.	Yes	ARRIVE guidelines have been followed; materials and methods
For **tumor marker prognostic studies,** we recommend that you follow the **REMARK** reporting guidelines (see link list at top right). See author guidelines, under 'Reporting Guidelines'. Please confirm you have followed these guidelines.	Not Applicable	
For **phase II and III randomized controlled trials**, please refer to the **CONSORT** flow diagram (see link list at top right) and submit the CONSORT checklist (see link list at top right) with your submission. See author guidelines, under 'Reporting Guidelines'. Please confirm you have submitted this list.	Not Applicable	

Data Availability

Data availability	Information included in the manuscript?	In which section is the information available? (Reagents and Tools Table, Materials and Methods, Figures, Data Availability Section)
Have **primary datasets** been deposited according to the journal's guidelines (see 'Data Deposition' section) and the respective accession numbers provided in the Data Availability Section?	Not Applicable	
Were **human clinical and genomic datasets** deposited in a public access-controlled repository in accordance to ethical obligations to the patients and to the applicable consent agreement?	Not Applicable	
Are **computational models** that are central and integral to a study available without restrictions in a machine-readable form? Were the relevant accession numbers or links provided?	Not Applicable	
If publicly available data were reused, provide the respective **data citations in the reference list**.	Not Applicable	
