# Supplementary material for: Disease modification and symptom relief in osteoarthritis using a mutated GCP‐2/CXCL6 chemokine
Source: EMBO Mol Med. 2022 Dec 12;15(1):e16218. doi: 10.15252/emmm.202216218 (PMC9832835; doi:10.15252/emmm.202216218)

"LinkTSG6 QC" 528 1 /Volumes/MIB-NMR_data/mib500a/data/becky/nmr

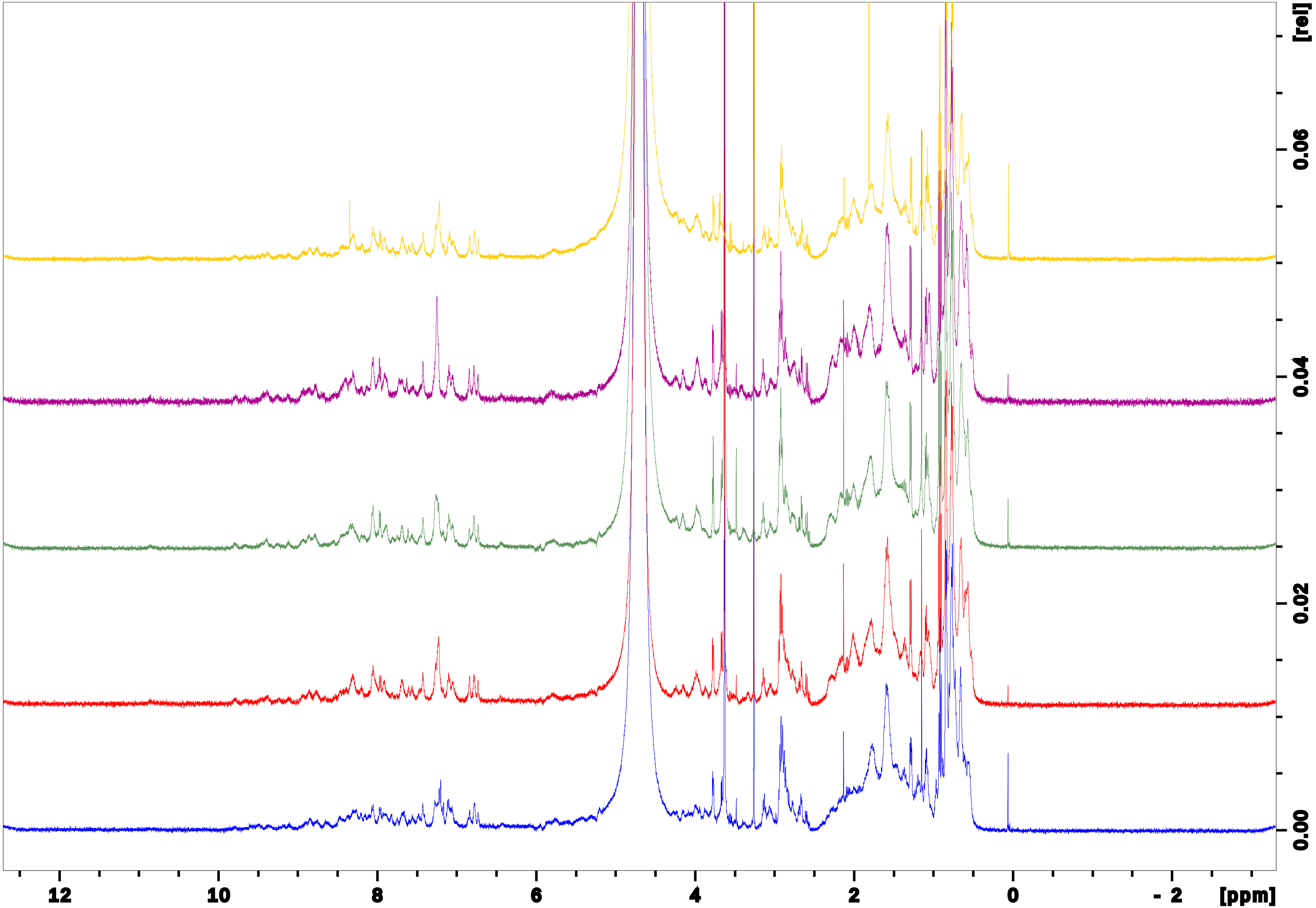

Supplement: Supplementary file 2 — Source Data for Expanded View [file EMMM-15-e16218-s006.zip › EV_2_zip.zip!/EV 2/s2b.pdf]
